# Supplementary material for: Viral Nuclease Inhibitors: Small molecule disruptors of the UL12 alkaline nuclease display broad anti-herpes virus activity
Source: PLoS Pathog. 2026 Aug 17;22(8):e1014531. doi: 10.1371/journal.ppat.1014531 (PMC13521474; doi:10.1371/journal.ppat.1014531)
Supplement: Supplementary file 1 [file ppat.1014531.s007.pdf]

Parameter	Value
Beamline	NSLS-II AMX (17-ID-1)
Wavelength (Å)	0.92010
Space group	P 21 21 21
Unit cell (Å, °)	a = 59.00, b = 80.23, c = 127.57; α = β = γ = 90°
**Data collection statistics**	
Resolution range (Å)	29.64–2.46 (2.56–2.46)
Unique reflections	22,700 (2508)
Completeness (%)	99.9 (99.2)
Multiplicity	1.9
Mean I/σ(I)	12.4 (2.4)
$CC_{1/2}$	0.999 (0.885)
Rmerge	0.037
Rpim / Rmeas	0.037 / 0.052 (0.316 / 0.446)
**MR phasing statistics**	
Top LLG	317
Top TFZ	19.12
**Refinement statistics**	
Reflections (work / free)	21,547 / 1,104 (4.85%)
R_{work} / R_{free}	0.181 / 0.238
CC_{work}	0.9616
CC_{free}	0.9342
Number of non-hydrogen atoms	3400
Macromolecules	3317
Ligands	18
Solvents	65
Average B-factor (Å^2)	62.0
Wilson B	49.4
Macromolecules	62.68
Ligands	79.26
Water	47.73
RMSD bond lengths	0.0107 Å
RMSD bond angles	2.239°
**Ramachandran plot**	
Ramachandran favored	95%
Ramachandran allowed	5%
Ramachandran outliers	0%
Clashscore	3
