# Supplementary material for: Viral Nuclease Inhibitors: Small molecule disruptors of the UL12 alkaline nuclease display broad anti-herpes virus activity
Source: PLoS Pathog. 2026 Aug 17;22(8):e1014531. doi: 10.1371/journal.ppat.1014531 (PMC13521474; doi:10.1371/journal.ppat.1014531)
Supplement: Supplementary file 2 [file ppat.1014531.s009.pdf]

SUPPORTING TEXT

Steady-state kinetics:

PicoGreen alkaline nuclease assay was used to obtain the Michaelis constant (K_m) for UL12 which then enabled the calculation of the equilibrium dissociation constant (K_i) of the UL12-inhibitor complex using the Cheng-Prusoff equation. The reactions were carried out in 96-well black, flat-bottom microplates at room temperature in a final volume of 200 µL. Linearized pUC119 served as the double-stranded DNA (dsDNA) substrate for the assays, with concentrations ranging from 0.25 nM to 1.875 nM, and UL12 was held at a fixed concentration of 5 nM. Prior to start of the reaction, the protein was preincubated in the dark with varying concentrations of pUC119 and PicoGreen in nuclease assay buffer containing 20 mM Tris-HCl (pH 8.2), 40 mM NaCl, 1 mM dithiothreitol (DTT). The final PicoGreen dilution was 1:115. The reaction was initiated by adding a final concentration of 1 mM $MgCl_2$. Fluorescence was recorded on a SpectraMax plate reader using Ex/Em = 480/520 nm, with measurements taken every 1 min 30 s for 60 min. A no-enzyme reaction mixture was included as a negative control and measured under identical conditions. K_m values were obtained by plotting initial reaction velocities as a function of DNA substrate concentration and fitting the data to the Michaelis-Menten equation using GraphPad Prism. For inhibition analysis, K_i values were estimated from experimentally determined IC_{50} values using the Cheng-Prusoff relationship:

$$Ki = \mathrm{IC50}/(1 + \frac{[S]}{Km})$$

which corrects the IC_{50} for the substrate concentration [S] used in the assay and the measured K_m of UL12 for the DNA substrate.

Synthesis:

Chemicals and solvents were purchased from commercial suppliers (Sigma Aldrich, Acros Organics, TCI Chemicals, Fisher Scientific, and AAblocks Inc) and were used as received unless otherwise stated. Compound 1 was purchased from Sigma Aldrich. Air and moisture-sensitive reactions, when required, were carried out under an inert atmosphere of argon in oven-dried glassware that was allowed to cool under argon. TLC analyses were performed on Sorbent Technologies silica gel HL TLC plates with fluorescent indicators (UV254). Developed plates were air-dried, and compounds were visualized under ultraviolet light (254 or 365 nm). Flash column chromatography was carried out using silica gel as the stationary phase. The ^{1}H and ^{13}C NMR spectra were recorded on Bruker instruments at 500 MHz. Chemical shifts are reported in ppm and are referenced to residual solvent signals ($CDCl_3$: δ 7.24 and 77.23 ppm for ^{1}H and ^{13}C; CD_3OD: δ 4.78, 3.31, and 49.15 ppm). Signal splitting patterns are described as singlet (s), doublet (d), triplet (t), quartet (q), multiplet (m), broad (b), or a combination thereof. Coupling constants (J) are quoted to the nearest 0.1 Hz. Liquid chromatography mass spectrometry (LC–MS) analyses were performed using a Waters ACQUITY UPLC system coupled to a Xevo G2-XS q-TOF mass spectrometer. Chromatographic separations were carried out on a Waters HSS T3 column (150 mm). LC-MS analyses were performed at a flow rate of 0.30 mL/min using a linear gradient of mobile phase B in A, where solvent A was water and solvent B was acetonitrile. The gradient was held at 5% B for 0.5 min, increased linearly from 5% to 95% B over 5.5 min, and held at 95% B for 0.5 min, followed by re-equilibration to 5% B until 8.0 min. Purity analyses were performed by reversed-phase high performance liquid chromatography (RP-HPLC) using a Shimadzu Prominence 20 instrument equipped with a Waters XSelect HSS T3 C18 column (5 μm, 4.6 mm × 250 mm) and monitored using a photodiode array (PDA) detector over a wavelength range of 190-800 nm. Mobile phase A consisted of water containing 0.05% trifluoroacetic acid (TFA), and mobile phase B consisted of acetonitrile containing 0.05% TFA. The gradient elution was performed at a flow rate of 2.50 mL/min as follows: 35% A/65% B from 0-2 min, 5% A/95% B

from 2-14 min, held at 5% A/95% B until 17 min, returned to 35% A/65% B by 19 min, and equilibrated at 35% A/65% B until 21 min. The total run time was 22 min. Samples were prepared in HPLC-grade DMSO prior to analysis. The purity of all final compounds tested in biological assays was ≥95%.

Procedure for Aminolysis and Ring Transformation to Dihydropyridines. Piperidinylmethyl-substituted dihydropyridine ester (**2**). To a suspension of 2-amino-2-phenylethan-1-ol (5.15 g, 25.5 mmol) in EtOH (40 mL) and HOAc (20 mL) was added the corresponding pyran starting material (3.0 g, 10.9 mmol). The reaction mixture was stirred at room temperature overnight and subsequently heated at 90 °C for 4 h. Upon completion, as monitored by TLC, the solvents were removed under reduced pressure. The residue was purified by flash column chromatography on silica gel (1-3% MeOH in 1:1 DCM/EtOAc) to afford compound 2 as a light yellow solid (3.5 g, 68%). ^{1}H NMR (500 MHz, $CDCl_3$) δ 8.55 (s, 1H), 8.28 (s, 1H), 7.49-7.24 (m, 5H), 4.98 (s, 2H), 4.26 (q, J = 7.1 Hz, 2H), 3.89-3.25 (m, 6H), 1.68-1.26 (m, 6H), 1.42 (s, 9H), 1.32 (t, J = 7.1 Hz, 3H). ^{13}C NMR (126 MHz, $CDCl_3$) δ 181.5, 163.8, 158.6, 154.8, 146.0, 142.0, 135.1, 128.5, 128.2, 127.2, 125.1, 79.8, 71.2, 62.3, 60.8, 44.1, 35.0, 31.7, 28.4, 14.1. HRMS (ESI) m/z $[M + H]^+$ calcd for $C_{26}H_{35}N_2O_6$ 471.2490; found 471.2565.

Procedure for Ester Hydrolysis to Dihydropyridine. Piperidinylmethyl-substituted dihydropyridine carboxylic acid (**3**). To a solution of compound 2 (3.5 g, 7.43 mmol) in ethanol (20 mL) was added an aqueous solution of NaOH (18.59 mmol). The resulting mixture was heated at 78 °C and stirred overnight. Upon completion, as monitored by TLC, the reaction mixture was cooled to room temperature, and the ethanol was removed under reduced pressure. The residue was diluted with water (20 mL), stirred, and cooled in an ice bath. The solution was acidified to pH 6 by dropwise addition of 1 N HCl (approximately 19 mL), resulting in the precipitation of a white solid. The solid was collected by filtration, washed with cold water, and dried under vacuum to afford compound 3 as a white solid (2.8 g, 85%). ^{1}H NMR (500 MHz, DMSO-d_6) δ 8.59 (s, 1H), 8.02 (s, 1H), 7.52-7.24 (m, 5H), 5.13 (s, 2H), 4.07-3.94 (m, 2H), 3.42-3.25 (m, 4H), 2.11 (m, 1H),

1.68-1.50 (m, 4H), 1.42 (s, 9H). ^{13}C NMR (126 MHz, DMSO-d_6) δ 181.5, 165.9, 158.6, 154.8, 146.0, 142.0, 135.1, 128.5, 128.2, 127.2, 120.4, 79.8, 71.2, 62.3, 44.1, 35.0, 31.7, 28.4. HRMS (ESI) m/z $[M + H]^+$ calcd for $C_{24}H_{31}N_2O_6$, 443.2177; found 443.2196.

Procedure for Amide Coupling of Dihydropyridine Carboxylic Acids. Arylamide-substituted dihydropyridine (**4**). To a solution of compound 3 (2.45 g, 5.53 mmol) in anhydrous DMSO (12 mL) were added DIPEA (2.97 mL, 16.6 mmol) and PyBOP (3.46 g, 6.64 mmol). The mixture was stirred at room temperature for 15 min, after which 4-bromoaniline (1.14 g, 6.64 mmol) and DMAP (0.13 g, 1.1 mmol) were added. The reaction mixture was stirred at room temperature for 8 h. TLC analysis (DCM/EtOAc = 1:1) indicated that approximately 10% of the starting material remained; therefore, an additional portion of PyBOP (0.34 g, 0.65 mmol) was added, and the reaction was stirred overnight. The reaction was quenched with water and extracted with EtOAc. The combined organic layers were washed sequentially with saturated aqueous $NaHCO_3$ solution and brine, dried over anhydrous Na_2SO_4, filtered, and concentrated under reduced pressure. Purification by flash column chromatography on silica gel (15% EtOAc in DCM) afforded compound 6 as a light yellow solid (2.45 g, 74%). ^1H NMR (500 MHz, DMSO-d_6) δ 12.94 (s, 1H), 8.55 (s, 1H), 8.52 (s, 1H), 8.28 (s, 1H), 7.84-7.72 (m, 4H), 7.45-7.24 (m, 5H), 4.98 (s, 2H), 3.89 (d, J = 3.5 Hz, 2H), 3.42-3.25 (m, 4H), 2.10 (m, 1H), 1.69-1.52 (m, 4H), 1.42 (s, 9H). ^{13}C NMR (126 MHz, DMSO-d_6) δ 181.5, 163.8, 158.6, 154.8, 146.0, 142.0, 135.1, 131.9, 128.5, 128.2, 127.4, 127.2, 121.8, 120.4, 116.3, 79.8, 71.2, 62.3, 44.1, 35.0, 31.7, 28.4. HRMS (ESI) m/z $[M + H]^+$ calcd for $C_{30}H_{35}BrN_3O_5$, 596.1735; found 596.1743.

Procedure for Boc Deprotection of Piperidine Derivatives. Secondary piperidine dihydropyridine amide (**5**). To a solution of compound 4 (2.45 g, 4.1 mmol) in dichloromethane (6 mL) was added trifluoroacetic acid (3.14 mL, 41.0 mmol). The reaction mixture was stirred at room temperature for 2 h. Upon completion, the mixture was concentrated under reduced pressure. The residue was suspended in water (30 mL), and the pH was adjusted to >8 by the addition of 1 N aqueous NaOH, resulting in the formation of a white precipitate. The solid was collected by

filtration, washed with water, and dried under vacuum to afford compound 5 as a white solid (2.00 g, 93%). ^{1}H NMR (500 MHz, DMSO-d_6) δ 12.92 (s, 1H), 8.59 (s, 1H), 8.28 (s, 1H), 8.52 (s, 1H), 7.72 (m, 2H), 7.46-7.24 (m, 7H), 5.09 (s, 2H), 4.10-4.09 (m, 2H), 3.88 (d, J = 3.2 Hz, 2H), 2.94-2.64 (m, 4H), 1.99 (m, 1H), 1.84-1.67 (m, 4H). ^{13}C NMR (126 MHz, DMSO-d_6) δ 181.5, 163.8, 158.6, 146.0, 142.0, 135.1, 131.9, 128.5, 128.2, 127.4, 127.2, 121.8, 120.4, 116.3, 71.2, 62.3, 46.6, 35.0, 33.4. HRMS (ESI) m/z: $[M + H]^+$ calcd for $C_{25}H_{27}BrN_3O_3$, 496.1236; found 496.1275.

General Procedure for N-Functionalization of Piperidine Dihydropyridine Amides. To a solution of the piperidine dihydropyridine amide (compound **5**, 1.0 mmol) in anhydrous DMF (5 mL) was added DIPEA (0.52 mL, 3.0 mmol), followed by portionwise addition of the corresponding sulfonyl chloride (1.1 mmol) at room temperature. The reaction mixture was stirred at room temperature overnight. Upon completion, the reaction mixture was poured into water (40 mL), and the resulting precipitate was collected by filtration. The crude solid was dissolved in EtOAc (50 mL) and washed sequentially with 5% aqueous $NaHCO_3$ and brine, dried over anhydrous Na_2SO_4, filtered, and concentrated under reduced pressure. The residue was further purified by trituration with methanol to remove a minor impurity observed by TLC. The purified solid was collected by filtration and dried under vacuum to afford the N-functionalized products (50-80% yield).

N-(4-bromophenyl)-5-(benzyloxy)-1-[4-bromo-3-(methylsulfonyl)phenyl]-3,6-dioxo-1,2,3,6-tetrahydropyridine-4-carboxamide (**6**). To a solution of compound **5** (0.50 g, 1.0 mmol) in anhydrous DMF (5 mL) was added DIPEA (0.52 mL, 3.0 mmol), followed by portionwise addition of methanesulfonyl chloride (0.085 mL, 1.1 mmol) at room temperature. The reaction mixture was stirred at room temperature overnight. Upon completion, the reaction mixture was poured into water (40 mL), and the resulting precipitate was collected by filtration. The crude solid was dissolved in EtOAc (50 mL) and washed sequentially with 5% aqueous $NaHCO_3$ and brine, dried over anhydrous Na_2SO_4, filtered, and concentrated under reduced pressure. The residue was further purified by trituration with methanol to remove a minor impurity observed by TLC. The

purified solid was collected by filtration and dried under vacuum as an off-white solid (517 mg, 82% yield). ^{1}H NMR (500 MHz, DMSO-d_6) δ 8.59 (s, 1H), 8.48 (s, 1H), 8.1-8.00 (m, 2H), 7.86-7.66 (m, 3H), 7.45-7.28 (m, 7H), 4.97 (s, 2H), 3.10 (s, 3H). ^{13}C NMR (126 MHz, DMSO-d_6) δ 181.5, 163.8, 158.6, 144.3, 142.0, 136.2, 135.1, 133.4, 131.9, 128.5, 128.2, 127.4, 127.2, 121.8, 120.7, 120.4, 117.7, 116.3, 116.0, 71.2, 44.2. HRMS (ESI) m/z $[M + H]^+$ calcd for $C_{26}H_{21}Br_2N_2O_5S$, 630.9573; found 630.9565.

N-(4-bromophenyl)-1-[(4-(methylsulfonyl)benzyl)]-3-(benzyloxy)-4-oxo-1,4-dihydropyridine-2-carboxamide (**7**). Yellow solid (67% yield) ^{1}H NMR (500 MHz, DMSO-d6) δ 3.08 (s, 3H), 4.92-5.03 (m, 4H), 7.24-7.50 (m, 9H), 7.59-7.78 (m, 4H), 8.30 (s, 1H), 8.50 (s, 1H). ^{13}C NMR (126 MHz, DMSO-d_6) δ 181.5, 163.8, 158.6, 146.0, 142.0, 137.4, 136.5, 135.1, 131.9, 129.4, 128.5, 128.2, 127.8, 127.4, 127.2, 121.8, 120.4, 116.3, 71.2, 53.5, 44.5. HRMS (ESI) m/z $[M + H]^+$ calcd for $C_{27}H_{24}BrN_2O_5S$ 565.0570; found 565.0576.

N-(4-bromophenyl)-1-[(2-(4-bromophenylsulfonyl)azetidin-1-yl)methyl]-3-(benzyloxy)-4-oxo-1,4-dihydropyridine-2-carboxamide (**8**). Yellow solid; yield 70%. ^{1}H NMR (500 MHz, DMSO-d_6) δ 2.12-2.47 (m, 5H), 3.40 (m, 1H), 3.90 (d, J = 3.2 Hz, 2H), 4.98 (s, 2H), 7.24-7.45 (m, 7H), 7.59 (m, 2H), 7.66-7.79 (m, 4H), 8.28 (s, 1H), 8.52 (s, 1H). ^{13}C NMR (126 MHz, DMSO-d_6) δ 181.5, 163.8, 158.6, 146.0, 142.0, 135.6, 135.1, 133.1, 131.9, 129.5, 128.5, 128.2, 127.8, 127.4, 127.2, 121.8, 120.4, 116.3, 71.2, 48.5, 39.0, 31.8, 29.8. HRMS (ESI) m/z $[M + H]^+$ calcd for $C_{29}H_{26}Br_2N_3O_5S$ 686.40; found 686.46.

N-(4-bromophenyl)-1-[1-(4-bromophenylsulfonyl)pyrrolidin-2-yl]-3-(benzyloxy)-4-oxo-1,4-dihydropyridine-2-carboxamide (**9**). Yellow solid; yield 60%. ^{1}H NMR (500 MHz, DMSO-d_6) δ 1.96-2.29 (m, 2H), 2.60 (m, 1H), 3.17-3.49 (m, 3H), 3.86-4.12 (m, 3H), 4.93-5.03 (m, 2H), 7.24-7.46 (m, 7H), 7.56 (m, 2H), 7.63-7.78 (m, 4H), 8.29 (s, 1H), 8.49 (s, 1H). ^{13}C NMR (126 MHz, DMSO-d_6) δ 181.5, 163.8, 158.6, 146.0, 142.0, 135.1, 133.6, 133.1, 131.9, 129.5, 128.5, 128.2, 127.8, 127.4, 127.2, 121.8, 120.4, 116.3, 71.2, 57.6, 56.5, 47.3, 37.1, 28.9. HRMS (ESI) m/z $[M + H]^+$ calcd for $C_{30}H_{28}Br_2N_3O_5S$ 700.0111; found 700.0124.

N-(4-bromophenyl)-1-[1-(4-bromophenylsulfonyl)pyrrolidin-2-yl]-3-(benzyloxy)-4-oxo-1,4-dihydropyridine-2-carboxamide (**10**). Yellow solid; yield 64%. ^{1}H NMR (500 MHz, DMSO-d_6) δ 1.61-1.79 (m, 4H), 2.10 (m, 1H), 3.22 (m, 2H), 3.59 (m, 2H), 3.89 (d, J = 3.2 Hz, 2H), 4.98 (s, 2H), 7.24-7.46 (m, 7H), 7.56 (m, 2H), 7.63-7.78 (m, 4H), 8.28 (s, 1H), 8.49 (s, 1H). ^{13}C NMR (126 MHz, DMSO-d_6) δ 181.5, 163.8, 158.6, 146.0, 142.0, 135.1, 133.1, 132.1, 131.9, 129.5, 128.5, 128.2, 127.8, 127.4, 127.2, 121.8, 120.4, 116.3, 71.2, 62.3, 44.5, 35.0, 31.7. HRMS (ESI) m/z $[M + H]^+$ calcd for $C_{31}H_{30}Br_2N_3O_5S$ 714.0267; found 714.0271.

N-(4-bromophenyl)-1-[1-(4-methylphenylsulfonyl)pyrrolidin-2-yl]-3-(benzyloxy)-4-oxo-1,4-dihydropyridine-2-carboxamide (**11**). Yellow solid; yield 60%. ^{1}H NMR (500 MHz, DMSO-d_6) δ 1.61-1.79 (m, 4H), 2.12 (m, 1H), 2.32 (s, 3H), 3.22 (m, 2H), 3.59 (m, 2H), 3.88 (d, J = 3.2 Hz, 2H), 7.25-7.43 (m, 4H), 7.63-7.84 (m, 5H), 8.51 (s, 1H). ^{13}C NMR (126 MHz, DMSO-d_6) δ 184.1, 163.8, 147.4, 146.0, 144.9, 142.0, 132.1, 131.9, 129.4, 127.7, 127.4, 121.8, 120.4, 116.3, 62.3, 44.5, 35.0, 31.7, 21.4. HRMS (ESI) m/z $[M + H]^+$ calcd for $C_{32}H_{34}BrN_3O_5S$ 652.1455; found 652.1459.

N-(4-bromophenyl)-1-[1-(4-fluorophenyl)sulfonylpyrrolidin-2-yl]-3-(benzyloxy)-4-oxo-1,4-dihydropyridine-2-carboxamide (**12**). Yellow solid; yield 64%. ^{1}H NMR (500 MHz, DMSO-d_6) δ 1.61-1.79 (m, 4H), 2.10 (m, 1H), 3.22 (m, 2H), 3.59 (m, 2H), 3.89 (d, J = 3.2 Hz, 2H), 4.98 (s, 2H), 7.24-7.46 (m, 7H), 7.56-7.78 (m, 6H), 8.28 (s, 1H), 8.49 (s, 1H). ^{13}C NMR (126 MHz, DMSO-d_6) δ 181.5, 165.7, 163.8, 158.6, 146.0, 142.0, 135.1, 132.1, 131.9, 128.5, 128.2, 127.8, 127.4, 127.2, 121.8, 120.4, 116.7, 116.3, 71.2, 62.3, 44.5, 35.0, 31.7. HRMS (ESI) m/z $[M + H]^+$ calcd for $C_{31}H_{30}BrFN_3O_5S$ 654.1073; found 654.1064.

N-(4-bromophenyl)-1-[1-(2-fluorophenyl)sulfonylpyrrolidin-2-yl]-3-(benzyloxy)-4-oxo-1,4-dihydropyridine-2-carboxamide (**13**). Yellow solid; yield 61%. ^{1}H NMR (500 MHz, DMSO-d_6) δ 1.61-1.79 (m, 4H), 2.10 (m, 1H), 3.22 (m, 2H), 3.59 (m, 2H), 3.89 (d, J = 3.2 Hz, 2H), 4.98 (s, 2H), 7.24-7.57 (m, 8H), 7.58-7.90 (m, 5H), 8.28 (s, 1H), 8.49 (s, 1H). ^{13}C NMR (126 MHz, DMSO-d_6) δ 181.5, 163.8, 159.2, 158.6, 146.0, 142.0, 136.2, 136.0, 135.1, 131.9, 129.5, 128.5, 128.2, 127.5,

127.4, 127.2, 121.8, 120.4, 117.3, 116.3, 71.2, 62.3, 44.5, 35.0, 31.7. HRMS (ESI) m/z $[M + H]^+$ calcd for $C_{31}H_{30}BrFN_3O_5S$ 654.1073; found 654.1075.

N-(4-bromophenyl)-1-[1-(2-nitrophenyl)sulfonylpyrrolidin-2-yl]-3-(benzyloxy)-4-oxo-1,4-dihydropyridine-2-carboxamide (**14**). Yellow solid; yield 55%. ^1H NMR (500 MHz, DMSO-d_6) δ 1.61-1.80 (m, 4H), 2.10 (m, 1H), 3.23 (m, 2H), 3.60 (m, 2H), 3.89 (d, J = 3.2 Hz, 2H), 4.98 (s, 2H), 7.24-7.46 (m, 7H), 7.66-7.92 (m, 4H), 7.96-8.15 (m, 2H), 8.28 (s, 1H), 8.49 (s, 1H). ^{13}C NMR (126 MHz, DMSO-d_6) δ 181.5, 163.8, 158.6, 146.0, 142.0, 137.8, 136.2, 135.1, 131.9, 129.5, 128.7-128.4 (m), 128.2, 127.5, 127.4, 127.2, 126.7, 121.8, 120.4, 116.3, 71.2, 62.3, 44.5, 35.0, 31.7. HRMS (ESI) m/z $[M + H]^+$ calcd for $C_{31}H_{30}BrN_4O_7S$ 682.0712; found 682.0720.

N-(4-bromophenyl)-1-[1-(4-methoxyphenyl)sulfonylpyrrolidin-2-yl]-3-(benzyloxy)-4-oxo-1,4-dihydropyridine-2-carboxamide (**15**). Yellow solid; yield 64%. ^1H NMR (500 MHz, DMSO-d_6) δ 1.61-1.79 (m, 4H), 2.10 (m, 1H), 3.22 (m, 2H), 3.59 (m, 2H), 3.89 (m, 2H), 3.84 (s, 3H), 4.98 (s, 2H), 6.97 (m, 2H), 7.24-7.46 (m, 7H), 7.59 (m, 2H), 7.72 (m, 2H), 8.28 (s, 1H), 8.49 (s, 1H). ^{13}C NMR (126 MHz, DMSO-d_6) δ 181.5, 163.9, 163.7, 158.6, 146.0, 142.0, 135.1, 132.1, 131.9, 128.5, 128.2, 127.8, 127.4, 127.2, 121.8, 120.4, 116.3, 114.5, 71.2, 62.3, 55.3, 44.5, 35.0, 31.7. HRMS (ESI) m/z $[M + H]^+$ calcd for $C_{32}H_{33}BrN_3O_6S$ 666.1273; found 666.1277.

N-(4-bromophenyl)-1-{[1-((2,3-dichlorophenyl)sulfonyl)piperidin-4-yl]methyl}-3-(benzyloxy)-4-oxo-1,4-dihydropyridine-2-carboxamide (**16**). Yellow solid (yield 60%). ^1H NMR (500 MHz, DMSO-d_6) δ 12.8-12.6 (br s, 1H), 8.45-8.20 (s, 1H), 7.90-7.20 (m, 12H, Ar-H), 5.00 (s, 2H, OCH_2Ph), 4.10-3.90 (m, 2H, NCH_2), 3.30-3.10 (m, 2H), 2.85-2.65 (m, 2H), 2.20-1.90 (m, 2H), 1.80-1.40 (m, 4H). ^{13}C NMR (126 MHz, DMSO-d_6) δ 181.5, 163.8, 158.6, 146.0, 142.0, 136.2, 135.1, 133.0, 131.9, 130.4, 129.6, 129.4, 128.9, 128.5, 128.2, 127.4, 127.2, 121.8, 120.4, 116.3, 71.2, 62.3, 44.5, 35.0, 31.7. HRMS (ESI) m/z: $[M + H]^+$ calcd for $C_{31}H_{29}BrCl_2N_3O_5S$, 704.0368; found 704.0360.

N-(4-bromophenyl)-1-[1-(isopropylsulfonyl)piperidin-4-yl]-3-(benzyloxy)-4-oxo-1,4-dihydropyridine-2-carboxamide (**17**). Yellow solid; yield 55%. ^1H NMR (500 MHz, $CDCl_3$) δ 8.52

(s, 1H), 8.28 (s, 1H), 7.72 (d, J = 8.4 Hz, 2H), 7.46-7.24 (m, 7H), 4.98 (s, 2H), 3.89 (d, J = 3.2 Hz, 2H), 3.83-3.52 (m, 3H), 3.24 (ddd, J = 14.8, 6.8, 6.8 Hz, 2H), 2.10 (m, 1H), 1.79-1.60 (m, 4H), 1.35 (d, J = 7.0 Hz, 6H). ^{13}C NMR ($CDCl_3$) δ 181.5, 163.8, 158.6, 146.0, 142.0, 135.1, 131.9, 128.5, 128.2, 127.4, 127.2, 121.8, 120.4, 116.3, 71.2, 62.3, 55.0, 44.5, 35.0, 31.7, 16.1. HRMS (ESI) m/z $[M + H]^+$ calcd for $C_{28}H_{33}BrN_3O_5S$ 602.1298; found 602.1306.

N-(4-fluorophenyl)-1-[1-(4-methylphenylsulfonyl)piperidin-4-yl]-3-(benzyloxy)-4-oxo-1,4-dihydropyridine-2-carboxamide (**18**). Yellow solid; yield 61%. ^{1}H NMR (500 MHz, $CDCl_3$) δ 8.52 (s, 1H), 8.28 (s, 1H), 7.79-7.63 (m, 4H), 7.46-7.24 (m, 7H), 7.02 (d, J = 8.5 Hz, 2H), 4.98 (s, 2H), 3.89 (d, J = 3.2 Hz, 2H), 3.59 (ddd, J = 14.8, 2.8, 2.8 Hz, 2H), 3.22 (ddd, J = 14.8, 6.8, 6.8 Hz, 2H), 2.32 (s, 3H), 2.09 (m, 1H), 1.79-1.61 (m, 4H). ^{13}C NMR ($CDCl_3$) δ 181.5, 163.8, 158.6, 157.8, 146.0, 144.9, 142.0, 135.1, 132.1, 129.4, 128.5, 128.2, 127.7, 127.4, 127.2, 120.6, 120.4, 115.1, 71.2, 62.3, 44.5, 35.0, 31.7, 21.4. HRMS (ESI) m/z $[M + H]^+$ calcd for $C_{32}H_{33}FN_3O_5S$ 590.2120; found 590.2128.

N-(4-chlorophenyl)-1-[1-(4-methylphenylsulfonyl)piperidin-4-yl]-3-(benzyloxy)-4-oxo-1,4-dihydropyridine-2-carboxamide (**19**). Yellow solid; yield 60%. ^{1}H NMR (500 MHz, $CDCl_3$) δ 8.52 (s, 1H), 8.28 (s, 1H), 7.78-7.62 (m, 4H), 7.46-7.24 (m, 9H), 4.98 (s, 2H), 3.89 (d, J = 3.2 Hz, 2H), 3.59 (ddd, J = 14.8, 2.8, 2.8 Hz, 2H), 3.22 (ddd, J = 14.8, 6.8, 6.8 Hz, 2H), 2.32 (s, 3H), 2.09 (m, 1H), 1.79–1.61 (m, 4H). ^{13}C NMR ($CDCl_3$) δ 181.5, 163.8, 158.6, 146.0, 144.9, 142.0, 135.1, 132.1, 129.4, 129.0, 128.5, 128.2, 127.7, 127.4, 127.2, 127.0, 121.6, 120.4, 71.2, 62.3, 44.5, 35.0, 31.7, 21.4. HRMS (ESI) m/z $[M + H]^+$ calcd for $C_{32}H_{33}ClN_3O_5S$ 606.1824; found 606.1830.

N-(benzothiophen-2-yl)-1-[1-(4-methylphenylsulfonyl)piperidin-4-yl]-3-(benzyloxy)-4-oxo-1,4-dihydropyridine-2-carboxamide (**20**). Yellow solid; yield 45%. ^1H NMR (500 MHz, $CDCl_3$) δ 8.50 (s, 1H), 8.29 (s, 1H), 8.05 (ddd, J = 8.1, 1.5, 0.4 Hz, 1H), 7.84-7.24 (m, 15H), 4.98 (s, 2H), 3.89 (d, J = 3.2 Hz, 2H), 3.59 (ddd, J = 14.8, 2.8, 2.8 Hz, 2H), 3.22 (ddd, J = 14.8, 6.8, 6.8 Hz, 2H), 2.32 (s, 3H), 2.09 (m, 1H), 1.79–1.61 (m, 4H). ^{13}C NMR (126 MHz, $CDCl_3$) δ 181.5, 163.8, 158.6, 146.0, 144.9, 142.0, 139.6, 136.8, 135.9, 135.7, 135.1, 134.9, 132.1, 129.4, 128.5, 128.2,

127.7, 127.2, 127.0, 124.5, 122.9, 121.9, 121.2, 120.4, 118.1, 111.3, 71.2, 62.3, 44.5, 35.0, 31.7, 21.4. HRMS (ESI) m/z $[M + H]^+$ calcd for $C_{38}H_{36}N_3O_5S_2$ 678.2091; found 678.2083.

N-(dibenzofuran-2-yl)-1-[1-(4-methylphenylsulfonyl)piperidin-4-yl]-3-(benzyloxy)-4-oxo-1,4-dihydropyridine-2-carboxamide (**21**). Yellow solid; yield 46%. ^1H NMR (500 MHz, $CDCl_3$) δ 8.50 (s, 1H), 8.29 (s, 1H), 7.96-7.61 (m, 7H), 7.55-7.16 (m, 9H), 4.98 (s, 2H), 3.89 (d, J = 3.2 Hz, 2H), 3.59 (ddd, J = 14.8, 2.8, 2.8 Hz, 2H), 3.22 (ddd, J = 14.8, 6.8, 6.8 Hz, 2H), 2.32 (s, 3H), 2.09 (m, 1H), 1.79–1.61 (m, 4H). ^{13}C NMR (126 MHz, $CDCl_3$) δ 181.5, 163.8, 158.6, 156.1, 155.2, 146.0, 144.9, 142.0, 136.8, 135.1, 132.1, 129.4, 128.5, 128.2, 127.7, 127.5, 127.2, 124.3, 124.2, 122.6, 122.4, 121.2, 120.4, 118.1, 111.6, 110.3, 71.2, 62.3, 44.5, 35.0, 31.7, 21.4. HRMS (ESI) m/z $[M + H]^+$ calcd for $C_{38}H_{37}N_3O_6S$ 662.2319; found 662.2325.

General Procedure for O-Deprotection of Benzyloxy Dihydropyridines. Hydroxy-substituted dihydropyridine amides. To (e.g., compound **6**, 0.5 mmol scale) was added trifluoroacetic acid (2 mL), and the reaction mixture was heated at 90 °C for 2 h. Upon completion, as confirmed by TLC, the mixture was cooled to room temperature and concentrated under reduced pressure. The crude off-white solid was suspended in methanol (5 mL) and neutralized with 30% aqueous ammonia (0.1 mL). The resulting precipitate was collected by filtration. The material was further purified by suspension in dichloromethane (40 mL), stirring at room temperature for 30 min, and standing overnight. The purified solids were collected by filtration and dried under vacuum.

N-(4-bromophenyl)-5-hydroxy-1-[4-bromo-3-(methylsulfonyl)phenyl]-3,6-dioxo-1,2,3,6-tetrahydropyridine-4-carboxamide **VNI-5158**. To compound **6** (0.50 g, 0.83 mmol) was added trifluoroacetic acid (2 mL), and the reaction mixture was heated at 90 °C for 2 h. Upon completion, as confirmed by TLC, the mixture was cooled to room temperature and concentrated under reduced pressure. The crude off-white solid was suspended in methanol (5 mL) and neutralized with 30% aqueous ammonia (0.1 mL). The resulting precipitate was collected by filtration. The material was further purified by suspension in dichloromethane (40 mL), stirring at room

temperature for 30 min, and standing overnight. The purified solid was collected by filtration and dried under vacuum to afford the final product as an off-white solid (250 mg, 62%). ^{1}H NMR (500 MHz, DMSO-d_6) δ 12.61 (s, 1H), 8.58 (s, 1H), 8.13 (d, J = 8.2 Hz, 2H), 7.94 (s, 1H), 7.79 (d, J = 8.2 Hz, 2H), 7.72 (d, J = 8.3 Hz, 2H), 7.37 (d, J = 8.3 Hz, 2H), 3.10 (s, 3H). ^{13}C NMR (126 MHz, DMSO-d_6) δ 171.01, 162.61, 149.77, 142.66, 141.16, 139.80, 138.28, 137.37, 132.40, 130.62, 126.73, 122.90, 122.04, 120.39, 116.25, 115.82, 42.37. HRMS (ESI) m/z $[M + H]^+$ calcd for $C_{19}H_{15}Br_2N_2O_5S$, 540.9068; found 540.9054.

N-(4-bromophenyl)-1-[(4-(methylsulfonyl)benzyl)]-3-hydroxy-4-oxo-1,4-dihydropyridine-2-carboxamide (**VNI-5159**). Yellow solid; yield 60%. ^{1}H NMR (500 MHz, DMSO-d_6) δ 12.71 (s, 1H), 8.60 (s, 1H), 7.89 (d, J = 8.3 Hz, 2H), 7.68 (s, 1H), 7.56 (t, J = 7.5 Hz, 4H), 7.46 (d, J = 8.6 Hz, 2H), 5.39 (s, 2H), 3.13 (s, 3H). ^{13}C NMR (126 MHz, DMSO-d_6) δ 184.1, 163.8, 147.4, 146.0, 142.0, 137.4, 136.5, 131.9, 129.4, 127.8, 127.4, 121.8, 120.4, 116.3, 53.5, 44.5. HRMS (ESI) m/z $[M + H]^+$ calcd for $C_{20}H_{18}BrN_2O_5S$ 477.0120; found 477.0117.

N-(4-bromophenyl)-1-[2-(4-bromophenylsulfonyl)azetidin-1-ylmethyl]-3-hydroxy-4-oxo-1,4-dihydropyridine-2-carboxamide (**VNI-5160**). Yellow solid; yield 66%. ^{1}H NMR (500 MHz, DMSO-d_6) δ 12.77 (s, 2H), 8.37 (d, J = 2.3 Hz, 2H), 7.81 (d, J = 8.5 Hz, 3H), 7.69-7.59 (m, 7H), 7.52 (d, J = 8.9 Hz, 4H), 4.16 (d, J = 6.6 Hz, 4H), 3.74 (t, J = 8.4 Hz, 4H), 3.51 (t, J = 7.3 Hz, 3H), 2.97-2.88 (m, 1H). ^{13}C NMR (126 MHz, DMSO-d_6) δ 169.51, 162.50, 149.20, 139.85, 138.02, 132.99, 132.56, 131.87, 129.99, 128.59, 127.73, 122.89, 121.50, 115.05, 114.78, 57.76, 52.99, 28.86. HRMS (ESI) m/z $[M + H]^+$ calcd for $C_{22}H_{20}Br_2N_3O_5S$ 597.9530; found 597.9464.

N-(4-bromophenyl)-1-[1-(4-bromophenylsulfonyl)pyrrolidin-2-yl]-3-hydroxy-4-oxo-1,4-dihydropyridine-2-carboxamide (**VNI-5161**). Yellow solid; yield 58%. ^{1}H NMR (500 MHz, DMSO-d_6) δ 12.80 (s, 1H), 8.40 (d, J = 2.2 Hz, 1H), 7.81 (d, J = 8.6 Hz, 1H), 7.71 (d, J = 8.6 Hz, 2H), 7.65-7.60 (m, 2H), 7.51 (d, J = 8.9 Hz, 2H), 4.11-3.97 (m, 1H), 3.27-3.20 (m, 1H), 3.13-3.05 (m, 1H), 2.92-2.84 (m, 1H), 2.55 (p, J = 7.3 Hz, 1H), 2.48 (dt, J = 3.7, 1.9 Hz, 2H), 1.79-1.70 (m, 1H), 1.47 (dq, J = 12.3, 8.5 Hz, 1H). ^{13}C NMR (126 MHz, DMSO-d_6) δ 169.55, 162.58, 149.76, 139.26,

138.78, 135.30, 133.57, 132.90, 130.30, 129.90, 129.30, 127.85, 122.35, 121.80, 114.80, 114.57, 58.26, 50.12, 45.15, 27.95. HRMS (ESI) m/z $[M + H]^+$ calcd for $C_{23}H_{22}Br_2N_3O_5S$ 609.9641; found 609.9672.

N-(4-bromophenyl)-1-[1-(4-bromophenylsulfonyl)pyrrolidin-2-yl]-3-hydroxy-4-oxo-1,4-dihydropyridine-2-carboxamide (**VNI-5162**). Off-white solid; yield 58%. ^{1}H NMR (500 MHz, DMSO-d_6) δ 12.84 (s, 1H), 7.85 (d, J = 8.5 Hz, 2H), 7.64 (t, J = 8.0 Hz, 3H), 7.54 (d, J = 8.9 Hz, 1H), 3.99 (d, J = 7.5 Hz, 2H), 3.64 (d, J = 11.7 Hz, 3H), 2.22 (t, J = 10.9 Hz, 1H), 1.53 (d, J = 10.8 Hz, 2H), 1.32-1.21 (m, 1H). ^{13}C NMR (126 MHz, DMSO-d_6) δ 169.94, 163.10, 149.64, 140.35, 138.48, 135.30, 132.93, 132.36, 129.84, 127.56, 123.58, 121.92, 115.53, 115.07, 61.71, 45.88, 40.51, 35.91, 28.25. HRMS (ESI) m/z $[M + H]^+$ calcd for $C_{24}H_{24}Br_2N_3O_5S$ 624.9835; found 624.9811.

N-(4-bromophenyl)-1-[1-(4-methylphenylsulfonyl)pyrrolidin-2-yl]-3-hydroxy-4-oxo-1,4-dihydropyridine-2-carboxamide (**VNI-5163**). Yellow solid; yield 66%. ^{1}H NMR (500 MHz, DMSO-d_6) δ 12.77 (s, 1H), 8.31 (s, 1H), 7.57-7.35 (m, 8H), 3.91 (m, 1H), 3.57 (m, 1H), 2.33 (s, 3H), 2.11-2.03 (m, 1H), 1.72 (m, 1H), 1.45 (m, 1H), 1.23-1.15 (m, 4H). ^{13}C NMR (126 MHz, DMSO-d_6) δ 169.93, 163.10, 149.63, 143.92, 140.35, 138.49, 133.03, 132.35, 130.24, 129.06, 127.90, 123.58, 121.91, 115.52, 115.06, 61.78, 55.38, 45.96, 36.03, 28.24, 21.46. HRMS (ESI) m/z $[M + H]^+$ calcd for $C_{25}H_{27}BrN_3O_5S$ 561.0928; found 561.0948.

N-(4-bromophenyl)-1-[1-(4-fluorophenyl)sulfonylpyrrolidin-2-yl]-3-hydroxy-4-oxo-1,4-dihydropyridine-2-carboxamide (**VNI-5164**). Yellow solid; yield 57%. ^{1}H NMR (500 MHz, $CDCl_3$) δ 12.03 (s, 1H), 8.32 (d, J = 2.3 Hz, 1H), 7.79-7.73 (m, 2H), 7.61 (d, J = 8.5 Hz, 2H), 7.46 (d, J = 8.4 Hz, 2H), 7.22-7.19 (m, 2H), 3.85 (dd, J = 20.6, 9.6 Hz, 4H), 2.30-2.21 (m, 2H), 1.68 (d, J = 13.1 Hz, 2H), 1.45 (tt, J = 13.9, 7.0 Hz, 2H). ^{13}C NMR (126 MHz, DMSO-d_6) δ 169.45, 162.61, 149.15, 139.86, 138.00, 131.86, 130.46, 130.38, 123.10, 121.43, 116.62, 116.44, 61.26, 45.43, 35.49, 27.73. HRMS (ESI) m/z $[M + H]^+$ calcd for $C_{24}H_{24}BrFN_3O_5S$ 564.0605; found 564.0642.

N-(4-bromophenyl)-1-[1-(2-fluorophenyl)sulfonylpyrrolidin-2-yl]-3-hydroxy-4-oxo-1,4-dihydropyridine-2-carboxamide (**VNI-5165**). Yellow solid; yield 58%. ^{1}H NMR (500 MHz, $CDCl_3$) δ 12.03 (s, 1H), 8.34 (d, J = 2.2 Hz, 1H), 7.84 (t, J = 6.9 Hz, 1H), 7.60 (dd, J = 14.0, 7.5 Hz, 3H), 7.49-7.42 (m, 2H), 7.24-7.19 (m, 2H), 3.97 (d, J = 12.7 Hz, 2H), 3.84 (d, J = 7.4 Hz, 2H), 2.56 (t, J = 12.3 Hz, 2H), 1.69 (d, J = 13.2 Hz, 2H), 1.43 (tt, J = 14.2, 7.1 Hz, 2H). ^{13}C NMR (126 MHz, DMSO-d_6) δ 169.46, 162.62, 149.14, 139.88, 138.00, 131.86, 130.73, 125.09, 123.14, 121.44, 117.62, 117.44, 115.04, 114.59, 61.24, 45.00, 35.55, 27.95. HRMS (ESI) m/z $[M + H]^+$ calcd for $C_{24}H_{24}BrFN_3O_5S$ 564.0605; found 564.0652.

N-(4-bromophenyl)-1-[1-(2-nitrophenyl)sulfonylpyrrolidin-2-yl]-3-hydroxy-4-oxo-1,4-dihydropyridine-2-carboxamide (**VNI-5166**). Yellow solid; yield 55%. ^{1}H NMR (500 MHz, DMSO-d_6) δ 12.85 (s, 1H), 8.41 (d, J = 2.2 Hz, 1H), 7.97 (ddd, J = 7.2, 5.4, 1.4 Hz, 2H), 7.86 (dtd, J = 26.1, 7.6, 1.4 Hz, 2H), 7.67-.59 (m, 3H), 7.53 (d, J = 8.5 Hz, 2H), 4.02 (d, J = 7.3 Hz, 2H), 2.70 (td, J = 12.4, 2.5 Hz, 2H), 1.95 (m, 1H), 1.61-1.54 (m, 2H), 1.25 (qd, J = 12.4, 4.1 Hz, 2H). ^{13}C NMR (126 MHz, DMSO-d_6) δ 169.47, 162.62, 149.16, 147.78, 139.89, 138.00, 134.63, 132.26, 131.85, 130.17, 129.80, 124.14, 123.14, 121.44, 115.04, 61.19, 45.15, 35.54, 28.03. HRMS (ESI) m/z $[M + H]^+$ calcd for $C_{24}H_{24}BrN_4O_7S$ 593.0477; found 593.0502.

N-(4-bromophenyl)-1-[1-(4-methoxyphenyl)sulfonylpyrrolidin-2-yl]-3-hydroxy-4-oxo-1,4-dihydropyridine-2-carboxamide (**VNI-5167**). Off-white solid; yield 62%. ^{1}H NMR (500 MHz, DMSO-d_6) δ 12.82 (s, 1H), 8.36 (s, 1H), 7.61 (m, 4H), 7.52 (d, J = 8.4 Hz, 2H), 7.11 (d, J = 8.4 Hz, 2H), 3.96 (d, J = 7.2 Hz, 2H), 3.82 (s, 3H), 3.60 (d, J = 11.4 Hz, 2H), 2.11 (t, J = 11.7 Hz, 2H), 1.50 (d, J = 12.7 Hz, 2H), 1.24 (d, J = 13.2 Hz, 2H). ^{13}C NMR (126 MHz, DMSO-d_6) δ 169.94, 163.12, 149.64, 140.35, 138.49, 132.35, 130.08, 127.42, 123.58, 121.91, 115.53, 115.07, 114.94, 61.80, 56.17, 45.98, 36.07, 28.24. HRMS (ESI) m/z $[M + H]^+$ calcd for $C_{25}H_{27}BrN_3O_6S$ 576.0798; found 576.0800.

N-(4-bromophenyl)-1-{[1-((2,3-dichlorophenyl)sulfonyl)piperidin-4-yl]methyl}-3-hydroxy-4-oxo-1,4-dihydropyridine-2-carboxamide (**VNI-5168**). Off-white solid; yield 64%. ^{1}H NMR (500

MHz, DMSO-d_6) δ 12.83 (s, 1H), 8.41 (d, J = 2.2 Hz, 1H), 7.94 (dd, J = 8.1, 4.1 Hz, 2H), 7.67-7.50 (m, 7H), 4.01 (d, J = 7.3 Hz, 2H), 3.71 (d, J = 12.5 Hz, 2H), 2.78 (t, J = 12.2 Hz, 2H), 1.53 (d, J = 12.7 Hz, 2H), 1.27-1.15 (m, 2H). ^{13}C NMR (126 MHz, DMSO-d_6) δ 169.98, 163.14, 149.68, 140.42, 138.99, 138.52, 135.25, 135.08, 132.38, 130.60, 129.62, 129.42, 129.08, 123.68, 121.95, 115.55, 115.13, 61.72, 45.41, 40.54, 36.28, 28.81. HRMS (ESI) m/z $[M + H]^+$ calcd for $C_{24}H_{23}BrCl_2N_3O_5S$ 613.9898; found 613.9943.

N-(4-bromophenyl)-1-[1-(isopropylsulfonyl)piperidin-4-yl]-3-hydroxy-4-oxo-1,4-dihydropyridine-2-carboxamide (**VNI-5170**). Yellow solid; yield 52%. ^{1}H NMR (500 MHz, DMSO-d_6) δ 12.88 (s, 1H), 8.47 (d, J = 2.2 Hz, 1H), 7.72 (d, J = 2.1 Hz, 1H), 7.66 (d, J = 8.5 Hz, 2H), 7.56 (d, J = 8.6 Hz, 2H), 4.06 (d, J = 7.5 Hz, 1H), 3.67 (d, J = 12.6 Hz, 1H), 3.30 (q, J = 6.8 Hz, 1H), 2.89-2.81 (m, 2H), 2.00 (s, 1H), 1.54 (d, J = 13.0 Hz, 1H), 1.22 (d, J = 6.8 Hz, 6H). ^{13}C NMR (126 MHz, DMSO-d_6) δ 169.96, 163.14, 149.67, 140.40, 138.51, 132.36, 123.69, 121.94, 115.54, 115.10, 61.90, 52.32, 45.59, 36.56, 29.27, 16.90. HRMS (ESI) m/z $[M + H]^+$ calcd for $C_{21}H_{27}BrN_3O_5S$ 512.0879; found 512.0854.

N-(4-fluorophenyl)-1-[1-(4-methylphenylsulfonyl)piperidin-4-yl]-3-hydroxy-4-oxo-1,4-dihydropyridine-2-carboxamide (**VNI-5171**). Yellow solid; yield 60%. ^{1}H NMR (500 MHz, DMSO-d_6) δ 12.75 (s, 1H), 8.38 (d, J = 2.2 Hz, 1H), 7.67 (dd, J = 8.9, 5.1 Hz, 2H), 7.62-7.57 (m, 3H), 7.42 (d, J = 8.1 Hz, 2H), 7.24-7.15 (m, 2H), 3.98 (d, J = 7.4 Hz, 2H), 3.63 (d, J = 11.6 Hz, 2H), 2.30 (s, 3H), 2.13 (td, J = 12.1, 2.5 Hz, 2H), 1.55-1.45 (m, 2H), 1.25 (qd, J = 12.4, 4.3 Hz, 2H). ^{13}C NMR (126 MHz, DMSO-d_6) δ 169.92, 162.89, 149.59, 143.92, 140.29, 135.58, 133.07, 130.25, 127.90, 123.52, 121.72, 116.22, 116.04, 61.77, 45.96, 36.04, 28.26, 21.46. HRMS (ESI) m/z $[M + H]^+$ calcd for $C_{25}H_{27}FN_3O_5S$ 500.1650; found 500.1689.

N-(4-chlorophenyl)-1-[1-(4-methylphenylsulfonyl)piperidin-4-yl]-3-hydroxy-4-oxo-1,4-dihydropyridine-2-carboxamide (**VNI-5172**). Yellow solid; yield 53%. ^{1}H NMR (500 MHz, $CDCl_3$) δ 11.99 (s, 1H), 8.31 (d, J = 6.8 Hz, 1H), 7.68-7.60 (m, 2H), 7.32 (dd, J = 8.3, 5.6 Hz, 2H), 7.19 (d, J = 3.6 Hz, 1H), 3.86 (d, J = 12.3 Hz, 1H), 3.81 (d, J = 7.3 Hz, 1H), 2.43 (s, 3H), 2.27-2.19 (m,

1H), 1.76 (d, J = 3.8 Hz, 1H), 1.68 (s, 1H), 1.44 (qd, J = 12.4, 4.2 Hz, 1H). ^{13}C NMR (126 MHz, DMSO-d_6) δ 169.61, 162.75, 149.29, 143.58, 140.01, 137.75, 132.72, 129.91, 129.11, 127.56, 127.19, 123.24, 121.20, 114.72, 61.45, 45.63, 35.69, 27.91, 21.13. HRMS (ESI) m/z $[M + H]^+$ calcd for $C_{25}H_{27}ClN_3O_5S$ 516.1354; found 516.1383.

N-(benzothiophen-2-yl)-1-[1-(4-methylphenylsulfonyl)piperidin-4-yl]-3-hydroxy-4-oxo-1,4-dihydropyridine-2-carboxamide (**VNI-5173**). Yellow solid; yield 46%. ^{1}H NMR (500 MHz, $CDCl_3$) δ 12.7 (s, 1H), 8.52 (s, 1H), 8.05 (ddd, J = 8.1, 1.5, 0.4 Hz, 1H), 7.85-7.25 (m, 11H), 3.88 (d, J = 3.2 Hz, 2H), 3.59 (ddd, J = 14.8, 2.8, 2.8 Hz, 2H), 3.22 (ddd, J = 14.8, 6.8, 6.8 Hz, 2H), 2.32 (s, 3H), 2.12 (m, 1H), 1.79-1.61 (m, 4H). ^{13}C NMR (126 MHz, $CDCl_3$) δ 184.1, 163.8, 147.4, 146.0, 144.9, 142.0, 139.6, 136.8, 135.9, 135.7, 134.9, 132.1, 129.4, 127.7, 127.0, 124.5, 122.9, 121.9, 121.2, 120.4, 118.1, 111.3, 62.3, 44.5, 35.0, 31.7, 21.4. HRMS (ESI) m/z $[M + H]^+$ calcd for $C_{31}H_{30}N_3O_5S_2$ 588.1508; found 588.1542.

N-(dibenzofuran-2-yl)-1-[1-(4-methylphenylsulfonyl)piperidin-4-yl]-3-hydroxy-4-oxo-1,4-dihydropyridine-2-carboxamide (**VNI-5174**). Yellow solid; yield 42%. ^{1}H NMR (500 MHz, $CDCl_3$) δ 12.7 (s, 1H), 8.52 (s, 1H), 8.05 (ddd, J = 8.1, 1.5, 0.4 Hz, 1H), 7.95-7.15 (m, 11H), 3.89 (d, J = 3.2 Hz, 2H), 3.59 (ddd, J = 14.8, 2.8, 2.8 Hz, 2H), 3.22 (ddd, J = 14.8, 6.8, 6.8 Hz, 2H), 2.32 (s, 3H), 2.09 (m, 1H), 1.79-1.61 (m, 4H). ^{13}C NMR (126 MHz, $CDCl_3$) δ 184.1, 163.8, 156.1, 155.2, 147.4, 146.0, 144.9, 142.0, 136.8, 132.1, 129.4, 127.7, 127.5, 124.3, 124.2, 122.6, 122.4, 121.2, 120.4, 118.1, 111.6, 110.3, 62.3, 44.5, 35.0, 31.7, 21.4. HRMS (ESI) m/z $[M + H]^+$ calcd for $C_{31}H_{30}N_3O_6S$ 572.1845; found 572.1848.
