# Supplementary material for: Viral Nuclease Inhibitors: Small molecule disruptors of the UL12 alkaline nuclease display broad anti-herpes virus activity
Source: PLoS Pathog. 2026 Aug 17;22(8):e1014531. doi: 10.1371/journal.ppat.1014531 (PMC13521474; doi:10.1371/journal.ppat.1014531)
Supplement: Supplementary file 3 [file ppat.1014531.s010.pdf]

Mass spectra for compounds

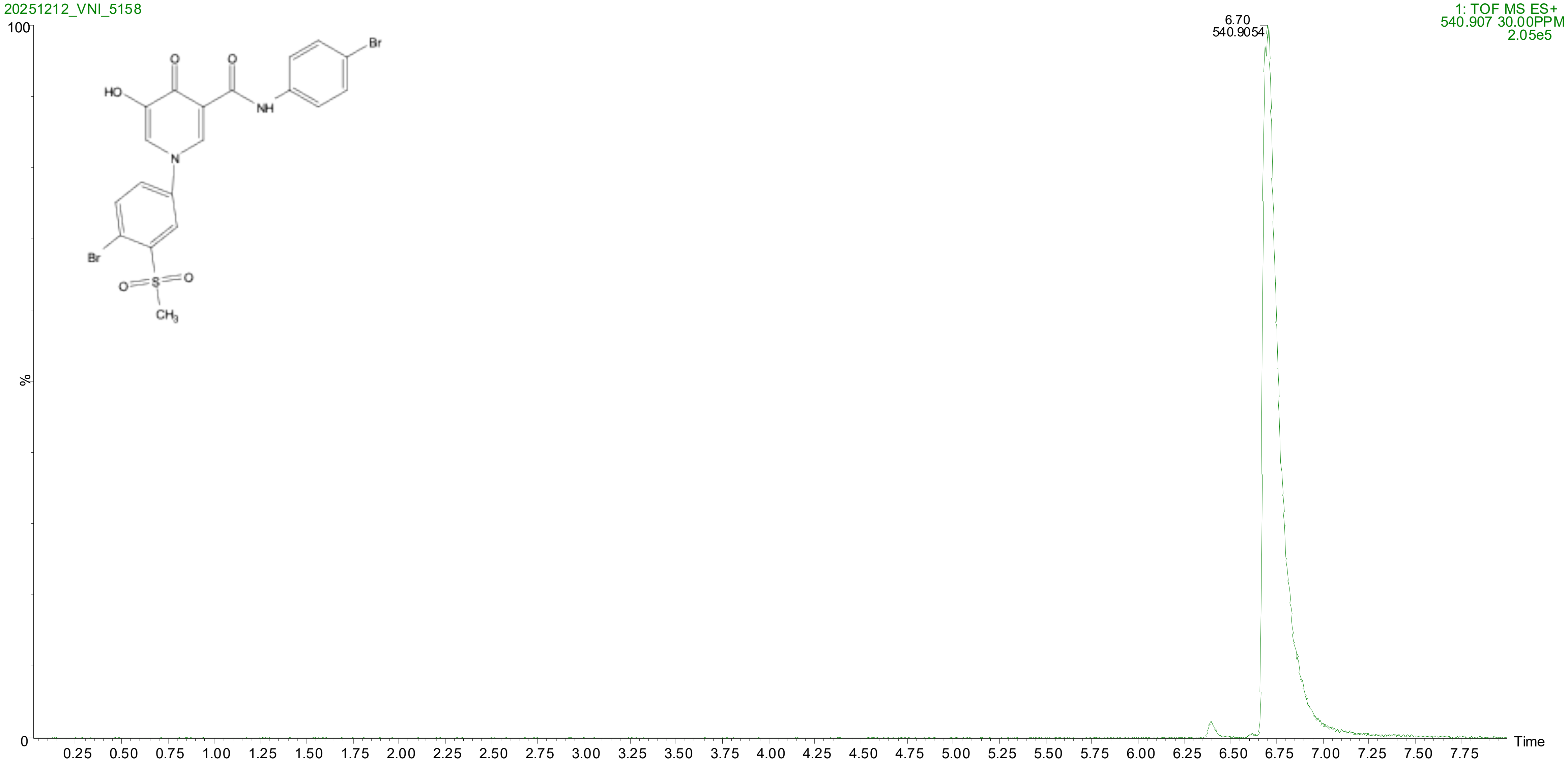
20251212_VNI_5158
1: TOF MS ES+
540.907 30.00PPM
2.05e5
6.70
540.9054
100
%
0
Time
0.25 0.50 0.75 1.00 1.25 1.50 1.75 2.00 2.25 2.50 2.75 3.00 3.25 3.50 3.75 4.00 4.25 4.50 4.75 5.00 5.25 5.50 5.75 6.00 6.25 6.50 6.75 7.00 7.25 7.50 7.75
HO
O
O
NH
Br
N
Br
O
S
O
CH3

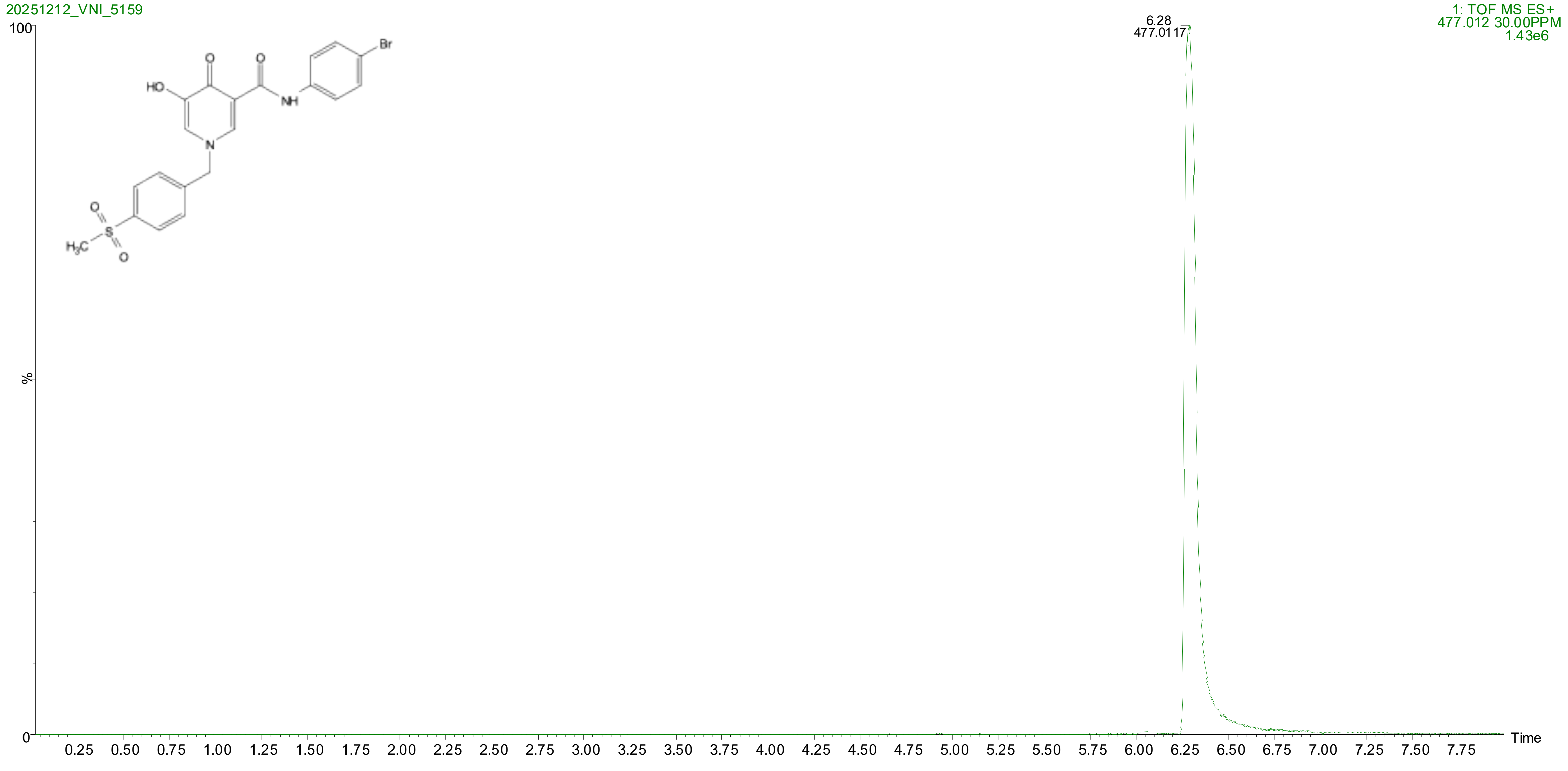

20251212_VNI_5159
1: TOF MS ES+
477.012 30.00PPM
1.43e6
6.28
477.0117
100
%
0
Time
0.25 0.50 0.75 1.00 1.25 1.50 1.75 2.00 2.25 2.50 2.75 3.00 3.25 3.50 3.75 4.00 4.25 4.50 4.75 5.00 5.25 5.50 5.75 6.00 6.25 6.50 6.75 7.00 7.25 7.50 7.75
HO
O
O
NH
Br
N
O
S
O
H3C

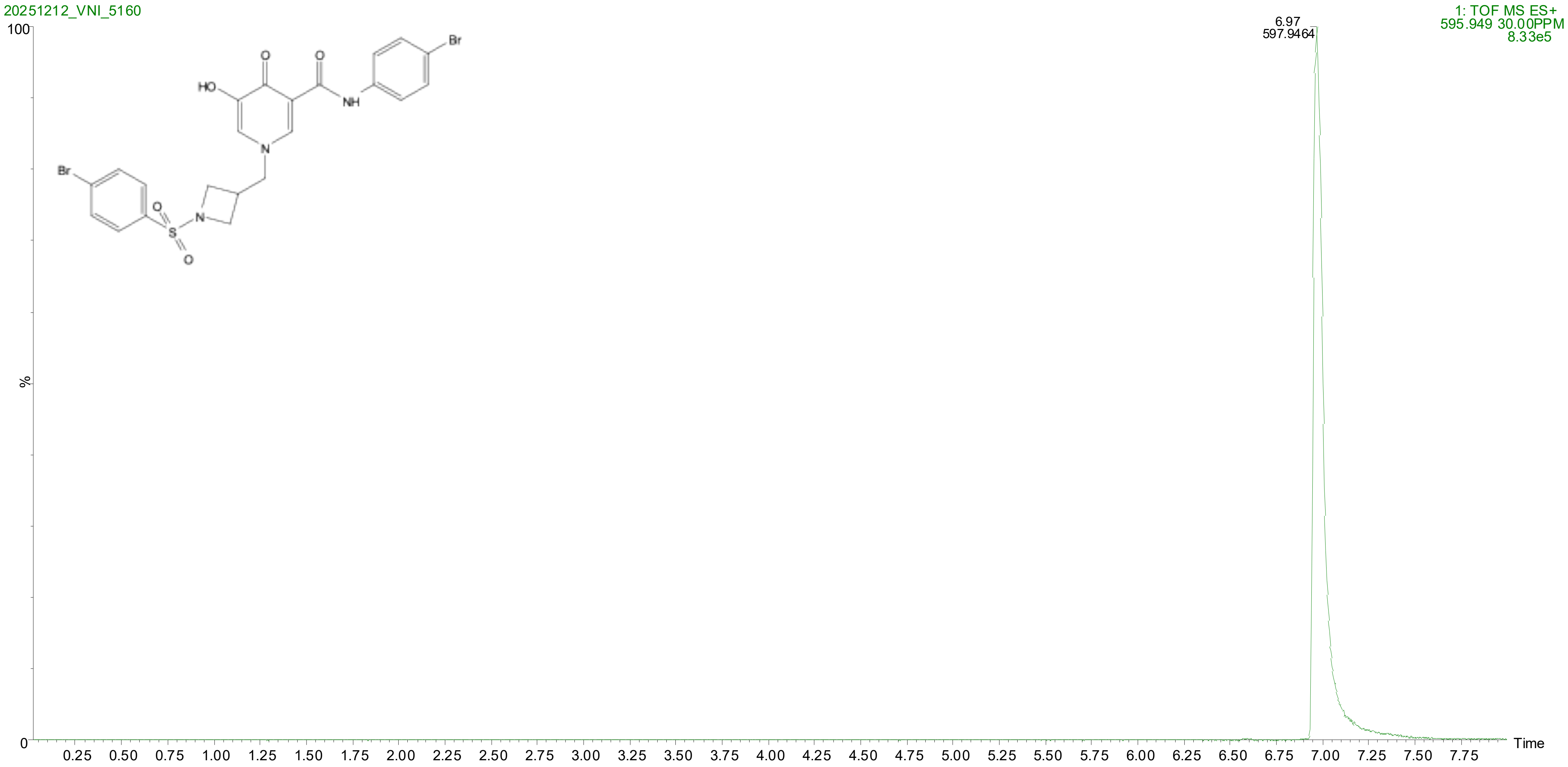

20251212_VNI_5160
1: TOF MS ES+
595.949 30.00PPM
8.33e5
6.97
597.9464
100
%
0
Time
0.25 0.50 0.75 1.00 1.25 1.50 1.75 2.00 2.25 2.50 2.75 3.00 3.25 3.50 3.75 4.00 4.25 4.50 4.75 5.00 5.25 5.50 5.75 6.00 6.25 6.50 6.75 7.00 7.25 7.50 7.75
HO
O
O
NH
Br
N
Br
O
S
O
N

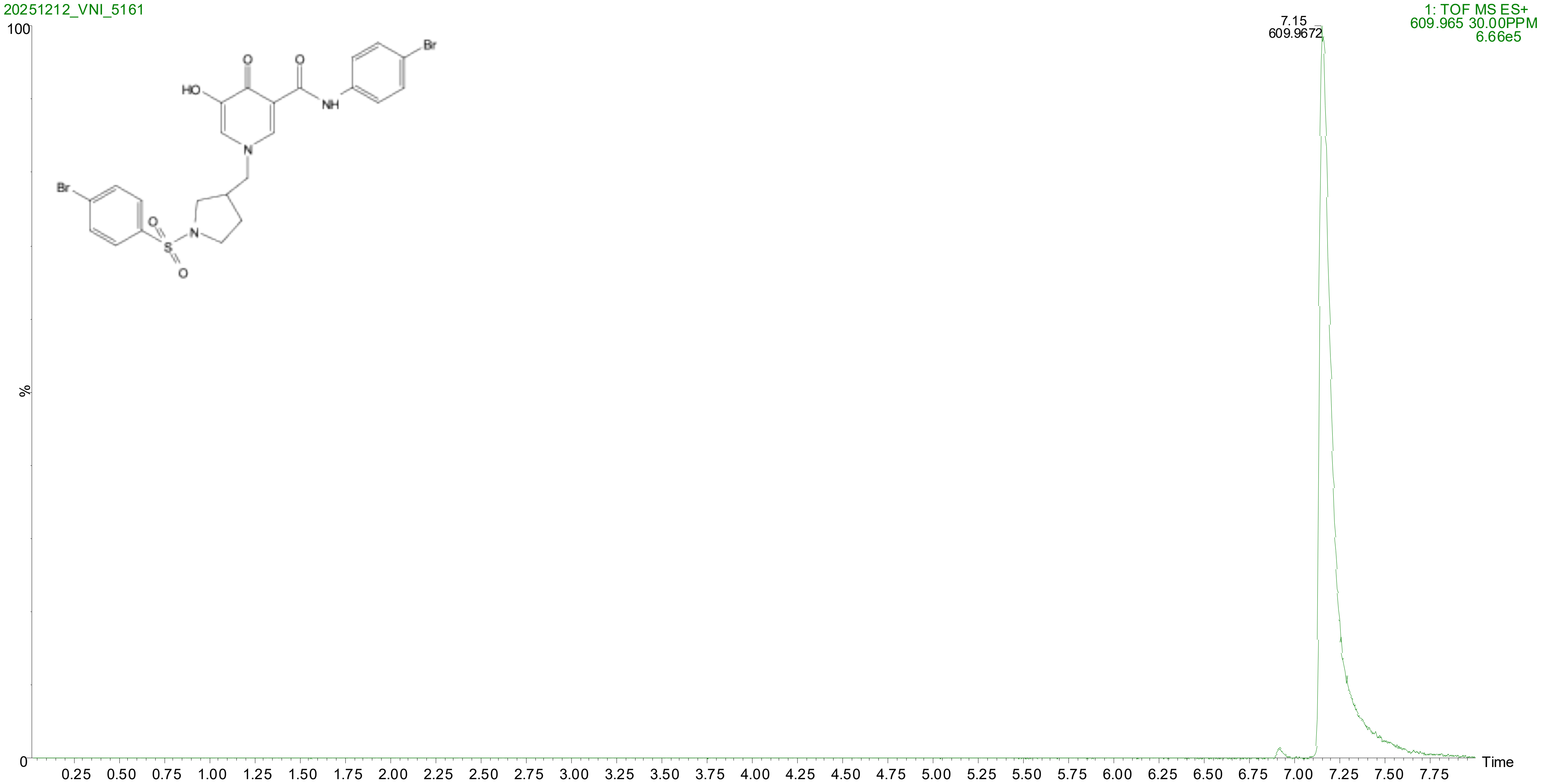
20251212_VNI_5161
1: TOF MS ES+
609.965 30.00PPM
6.66e5
7.15
609.9672
100
%
0
Time
0.25 0.50 0.75 1.00 1.25 1.50 1.75 2.00 2.25 2.50 2.75 3.00 3.25 3.50 3.75 4.00 4.25 4.50 4.75 5.00 5.25 5.50 5.75 6.00 6.25 6.50 6.75 7.00 7.25 7.50 7.75
HO
O
O
NH
Br
N
Br
O
S
O
N

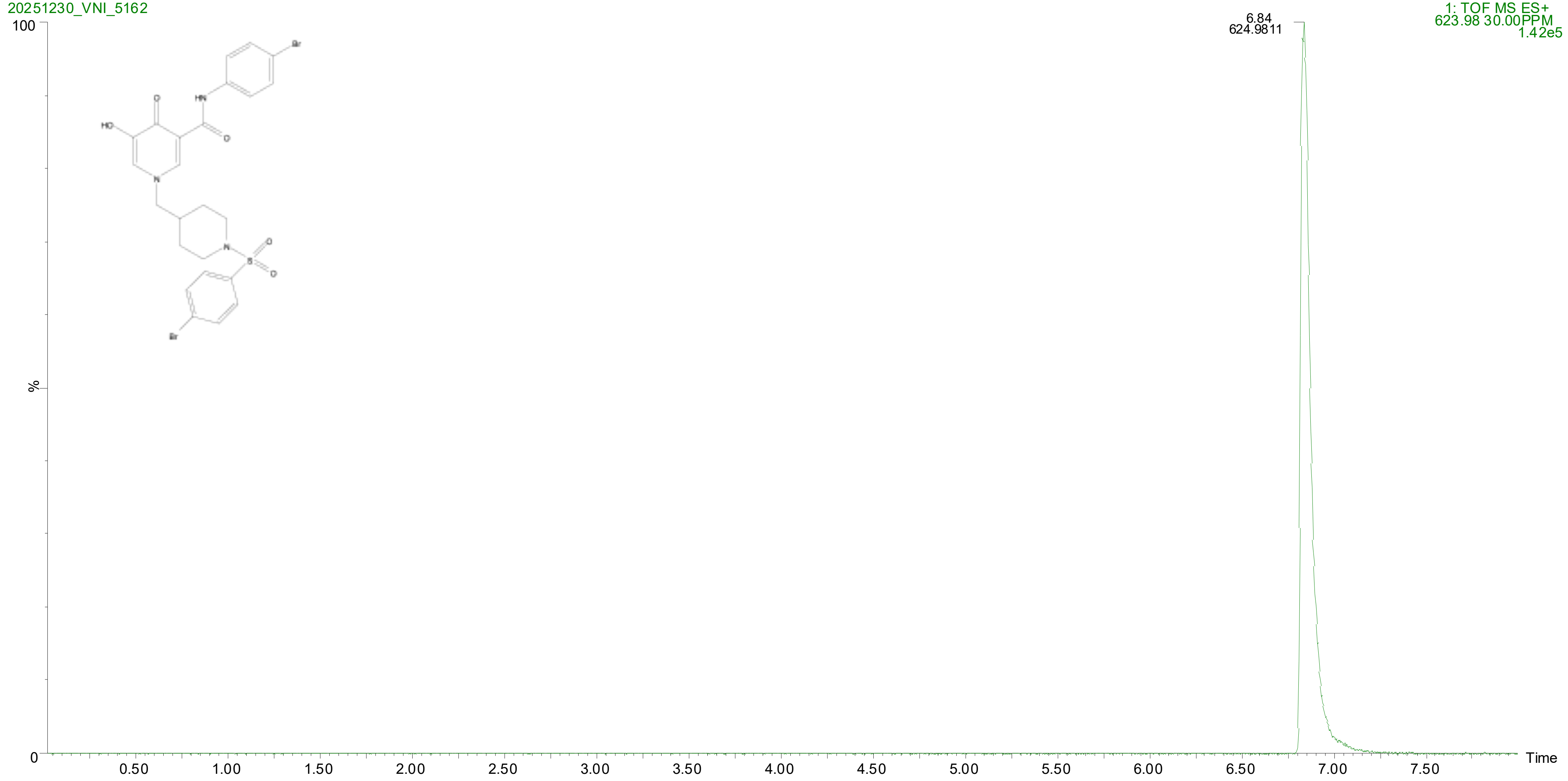
20251230_VNI_5162
1: TOF MS ES+
623.98 30.00PPM
1.42e5
6.84
624.9811
100
%
0
0.50
1.00
1.50
2.00
2.50
3.00
3.50
4.00
4.50
5.00
5.50
6.00
6.50
7.00
7.50
Time

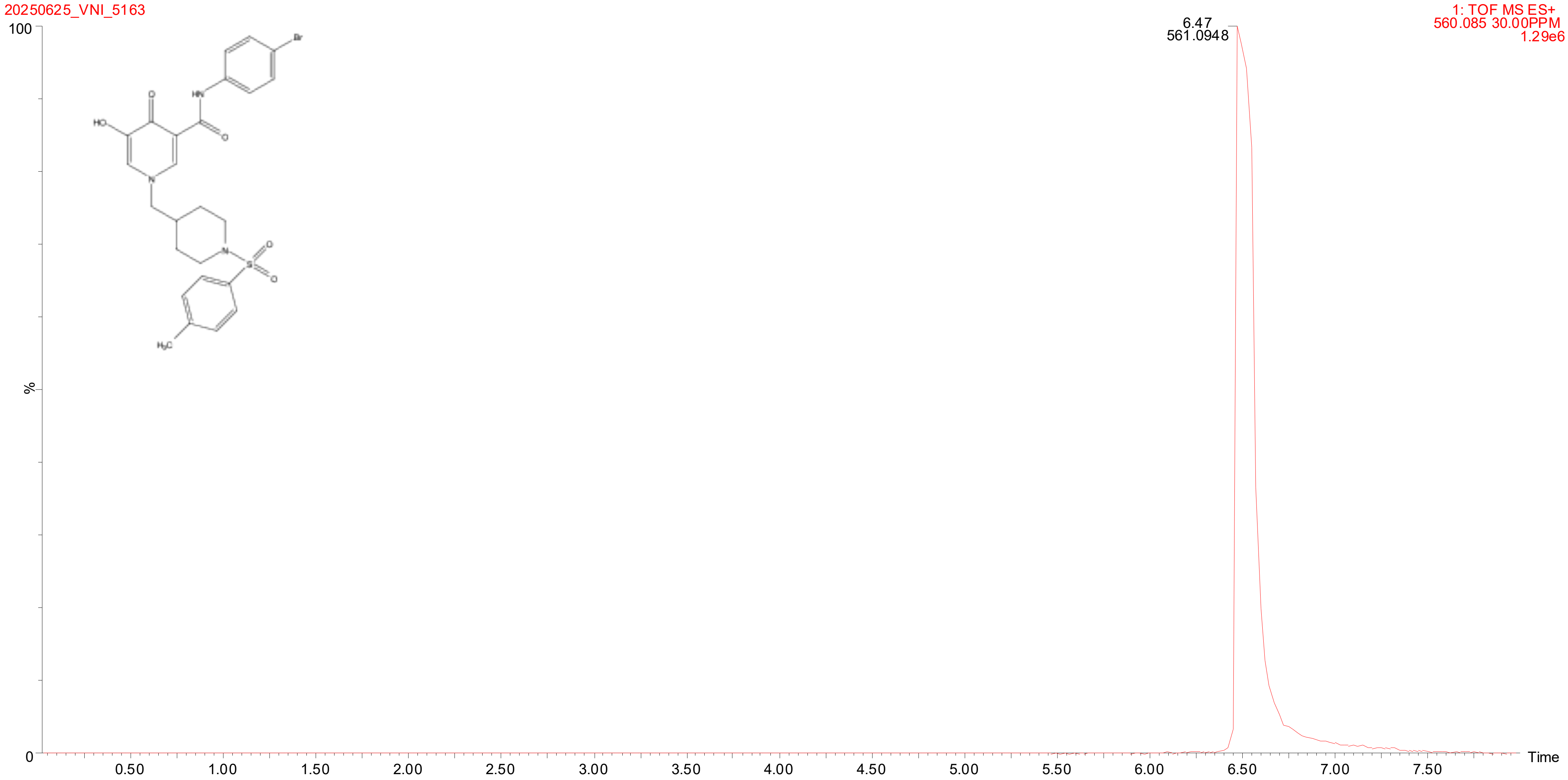
20250625_VNI_5163
1: TOF MS ES+
560.085 30.00PPM
1.29e6
6.47
561.0948
100
%
0
0.50
1.00
1.50
2.00
2.50
3.00
3.50
4.00
4.50
5.00
5.50
6.00
6.50
7.00
7.50
Time
Br
HN
O
HO
O
N
N
S
O
O
H3C

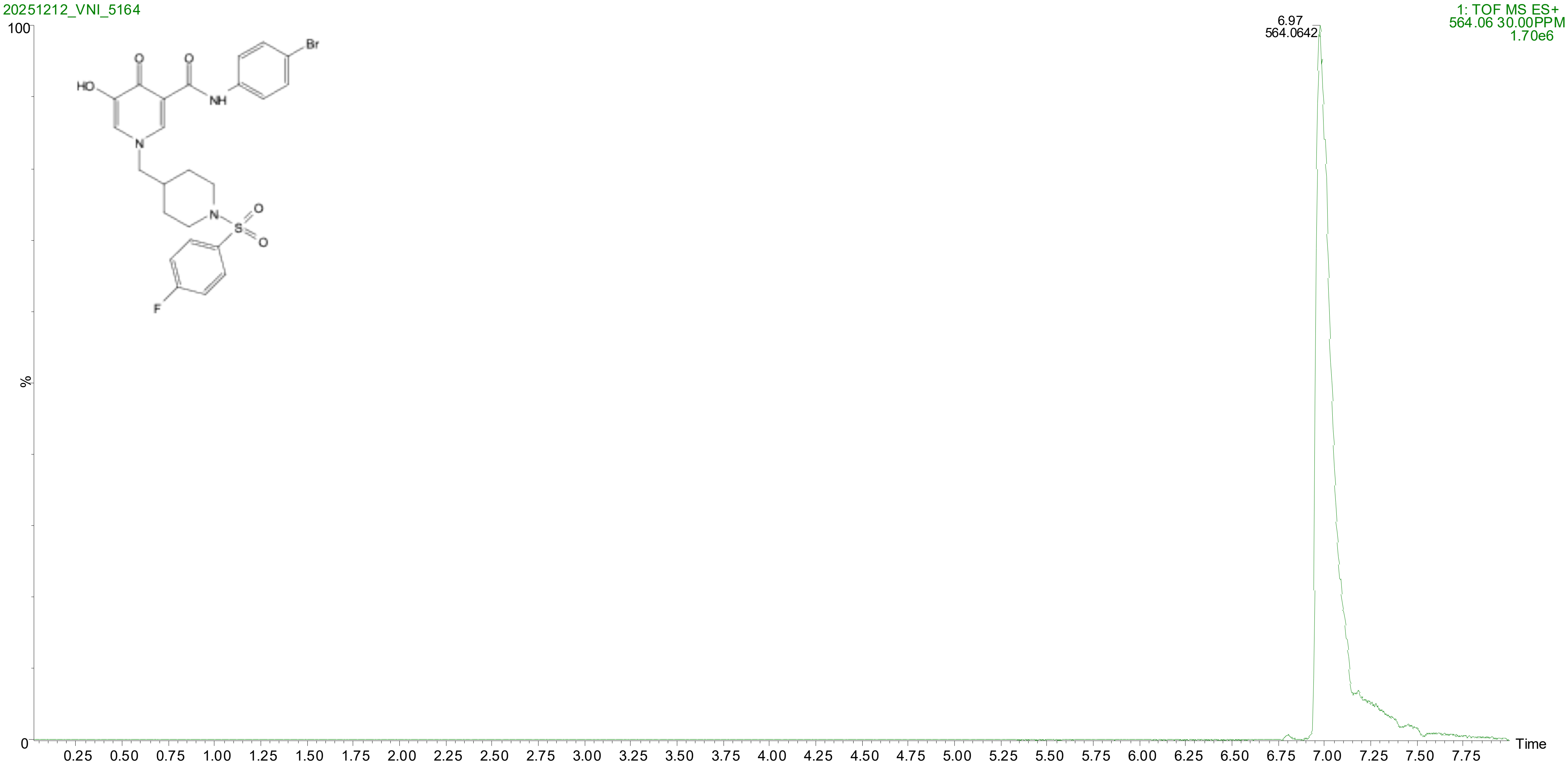
20251212_VNI_5164
1: TOF MS ES+
564.06 30.00PPM
1.70e6
6.97
564.0642
100
%
0
Time
0.25 0.50 0.75 1.00 1.25 1.50 1.75 2.00 2.25 2.50 2.75 3.00 3.25 3.50 3.75 4.00 4.25 4.50 4.75 5.00 5.25 5.50 5.75 6.00 6.25 6.50 6.75 7.00 7.25 7.50 7.75

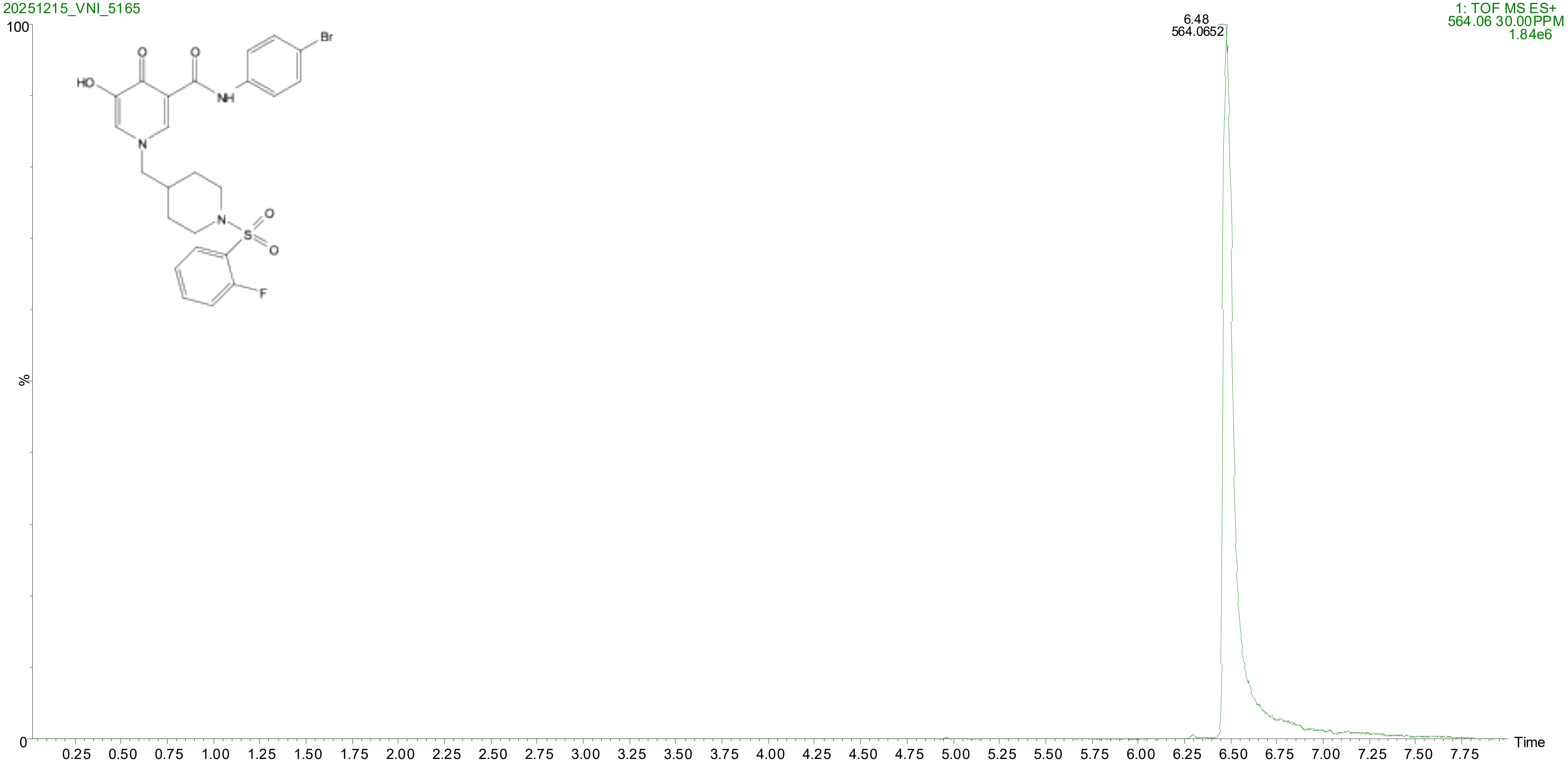
20251215_VNI_5165
1: TOF MS ES+
564.06 30.00PPM
1.84e6
6.48
564.0652
100
%
0
0.25 0.50 0.75 1.00 1.25 1.50 1.75 2.00 2.25 2.50 2.75 3.00 3.25 3.50 3.75 4.00 4.25 4.50 4.75 5.00 5.25 5.50 5.75 6.00 6.25 6.50 6.75 7.00 7.25 7.50 7.75
Time
HO
O
O
NH
Br
N
N
S
O
O
F

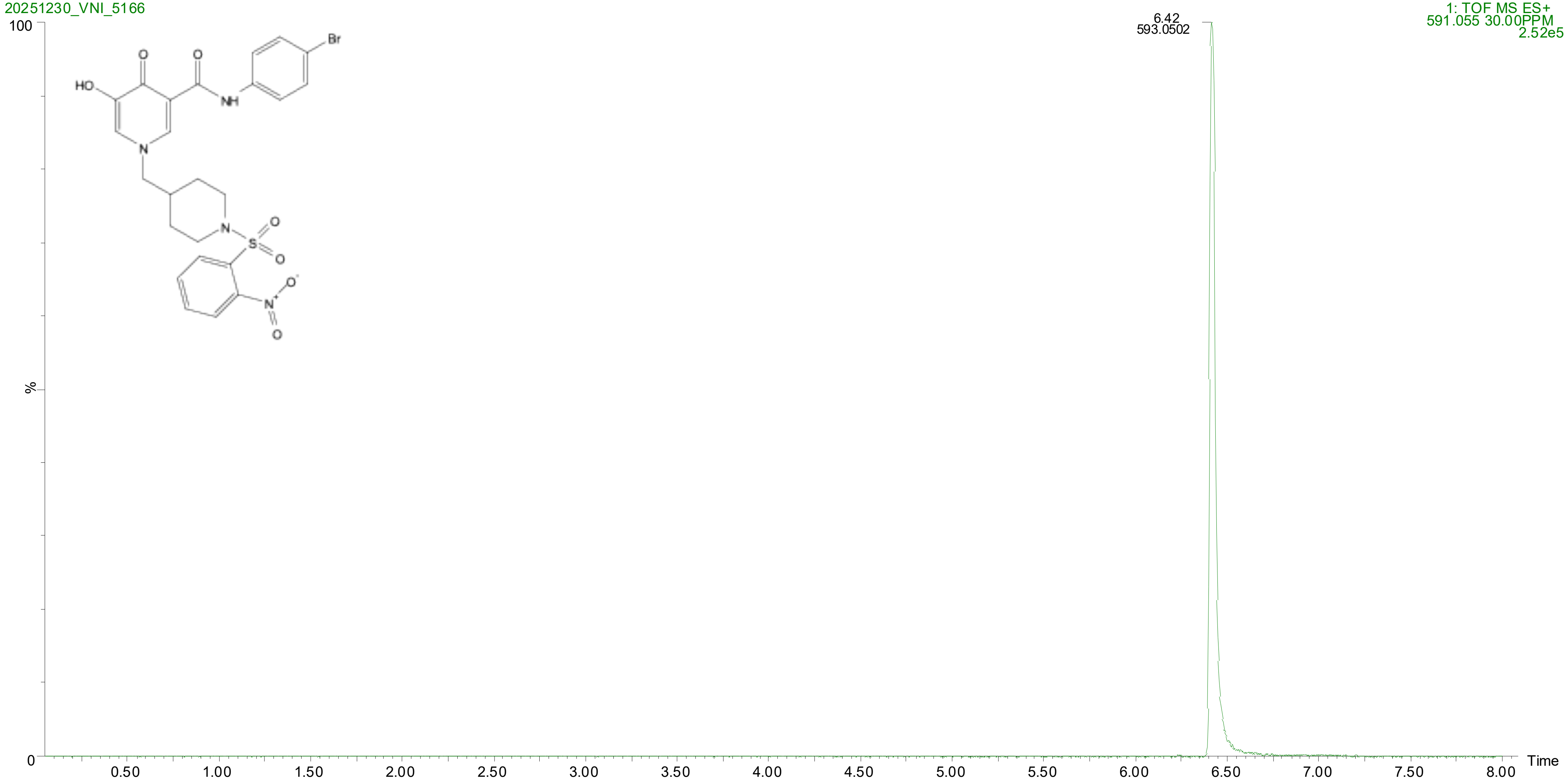
20251230_VNI_5166
1: TOF MS ES+
591.055 30.00PPM
2.52e5
6.42
593.0502
100
%
0
0.50
1.00
1.50
2.00
2.50
3.00
3.50
4.00
4.50
5.00
5.50
6.00
6.50
7.00
7.50
8.00
Time
HO
O
O
NH
Br
N
N
S
O
O
N
O
O

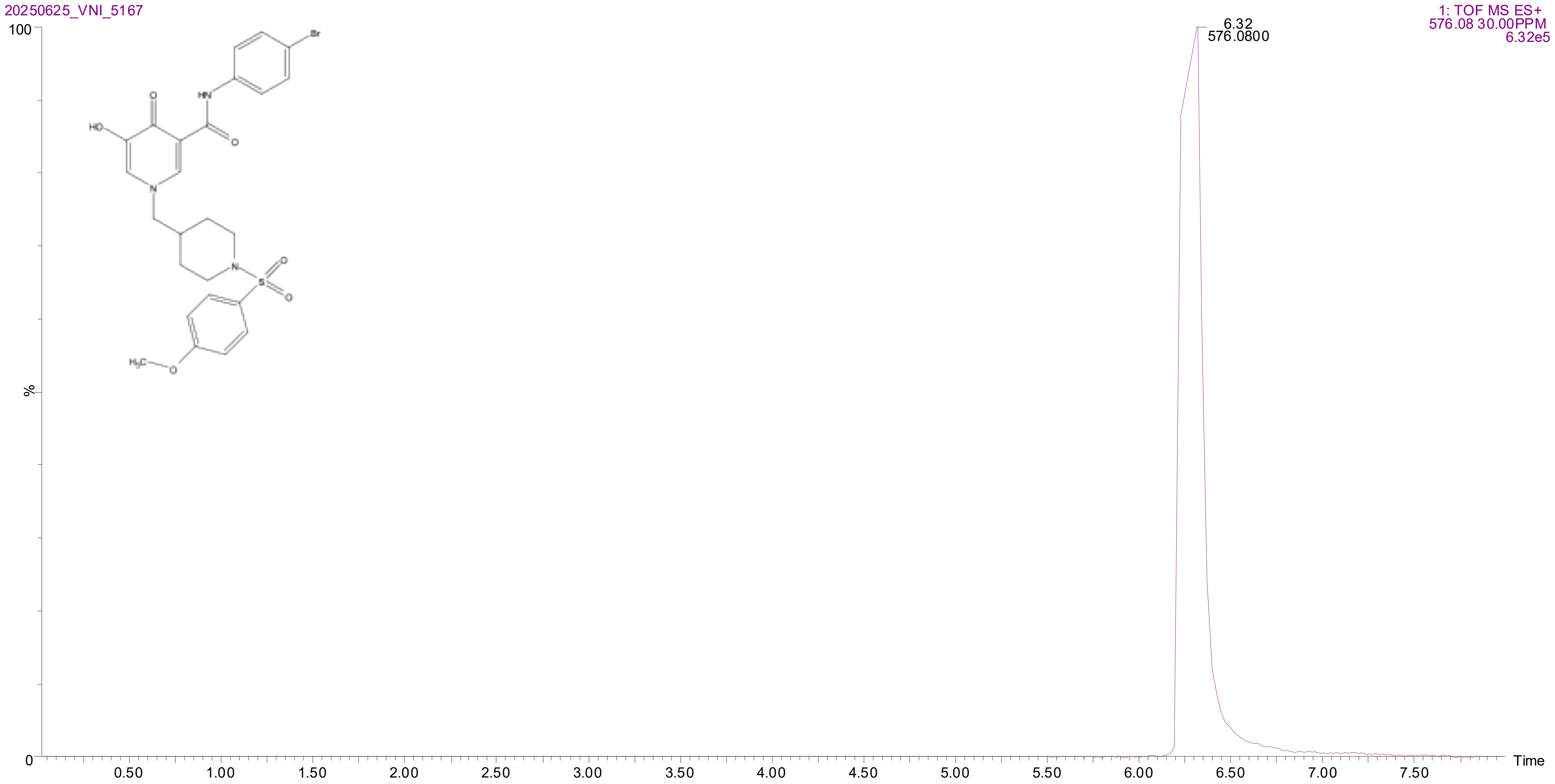
20250625_VNI_5167
1: TOF MS ES+
576.08 30.00PPM
6.32e5
6.32
576.0800
100
%
0
0.50
1.00
1.50
2.00
2.50
3.00
3.50
4.00
4.50
5.00
5.50
6.00
6.50
7.00
7.50
Time
Br
HN
O
HO
N
S
H3C

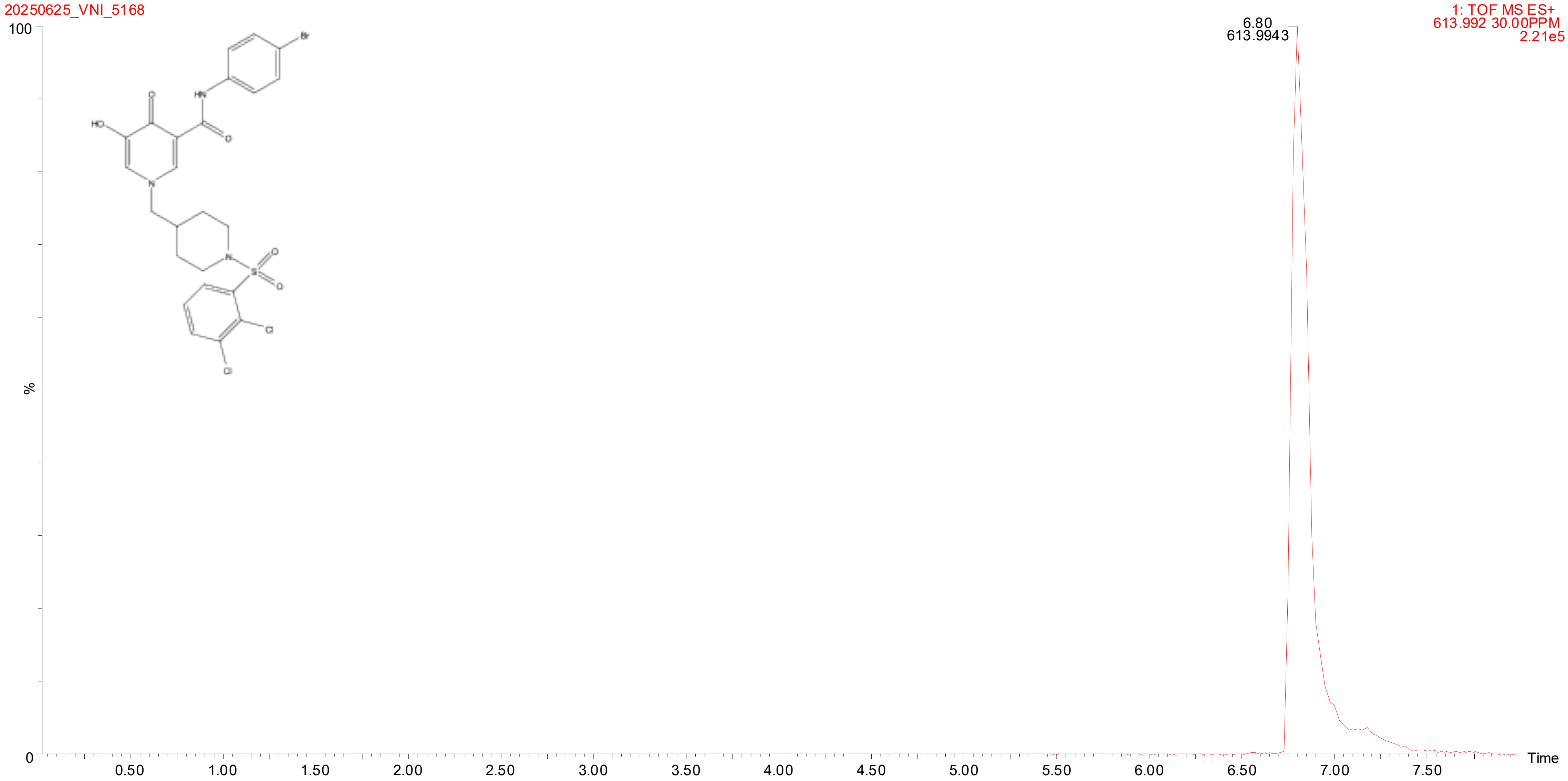
20250625_VNI_5168
1: TOF MS ES+
613.992 30.00PPM
2.21e5
6.80
613.9943
100
%
0
Time
0.50
1.00
1.50
2.00
2.50
3.00
3.50
4.00
4.50
5.00
5.50
6.00
6.50
7.00
7.50
Br
HN
O
HO
N
S
Cl
Cl

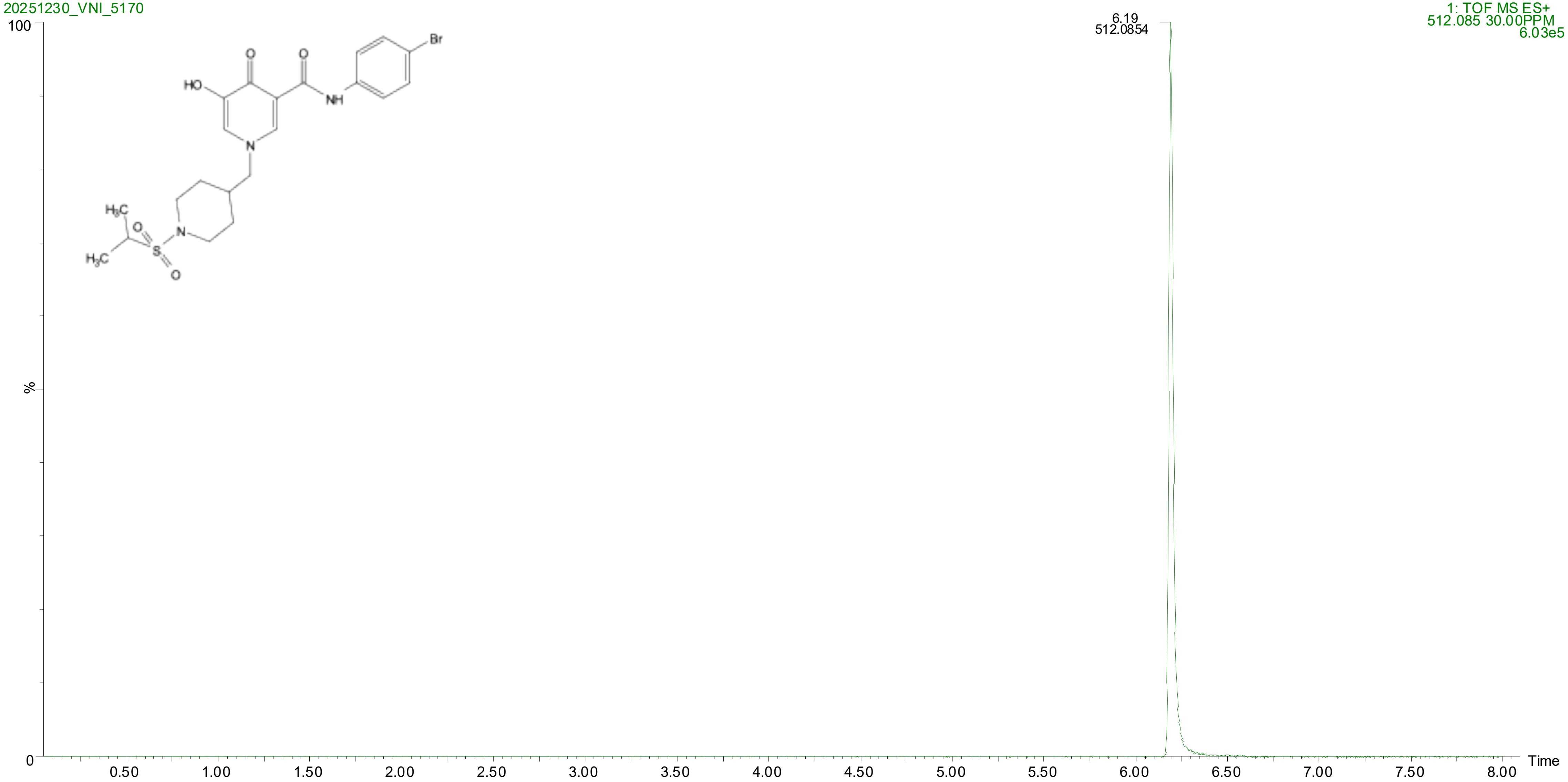
20251230_VNI_5170
1: TOF MS ES+
512.085 30.00PPM
6.03e5
6.19
512.0854
100
%
0
Time
0.50
1.00
1.50
2.00
2.50
3.00
3.50
4.00
4.50
5.00
5.50
6.00
6.50
7.00
7.50
8.00
HO
O
O
NH
Br
N
N
S
O
O
H3C
H3C

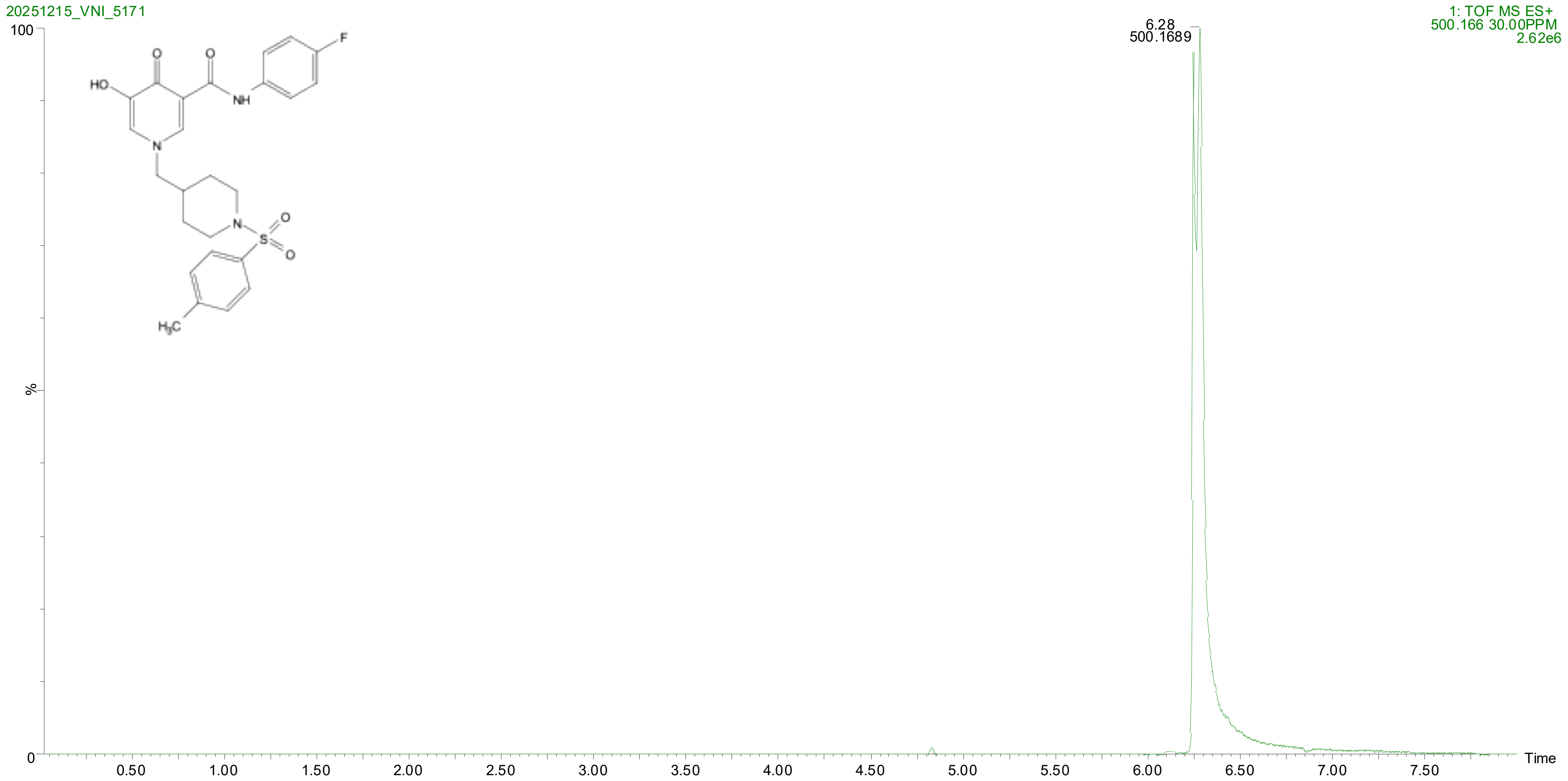
20251215_VNI_5171
1: TOF MS ES+
500.166 30.00PPM
2.62e6
6.28
500.1689
100
%
0
Time
0.50
1.00
1.50
2.00
2.50
3.00
3.50
4.00
4.50
5.00
5.50
6.00
6.50
7.00
7.50
HO
O
O
NH
F
N
N
S
O
O
H_3C

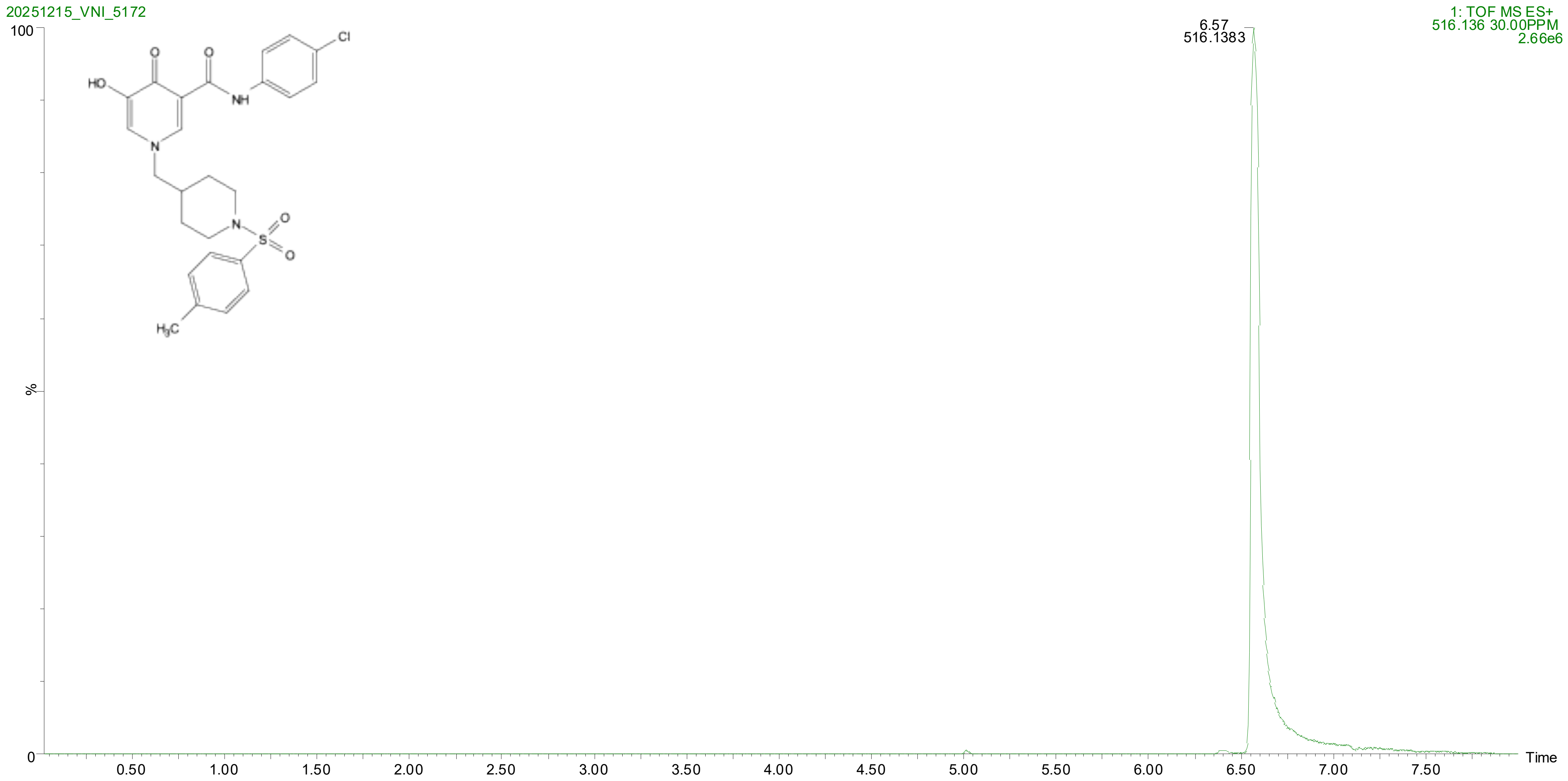
20251215_VNI_5172
1: TOF MS ES+
516.136 30.00PPM
2.66e6
6.57
516.1383
100
%
0
Time
0.50
1.00
1.50
2.00
2.50
3.00
3.50
4.00
4.50
5.00
5.50
6.00
6.50
7.00
7.50
HO
O
O
NH
Cl
N
N
S
O
O
H3C

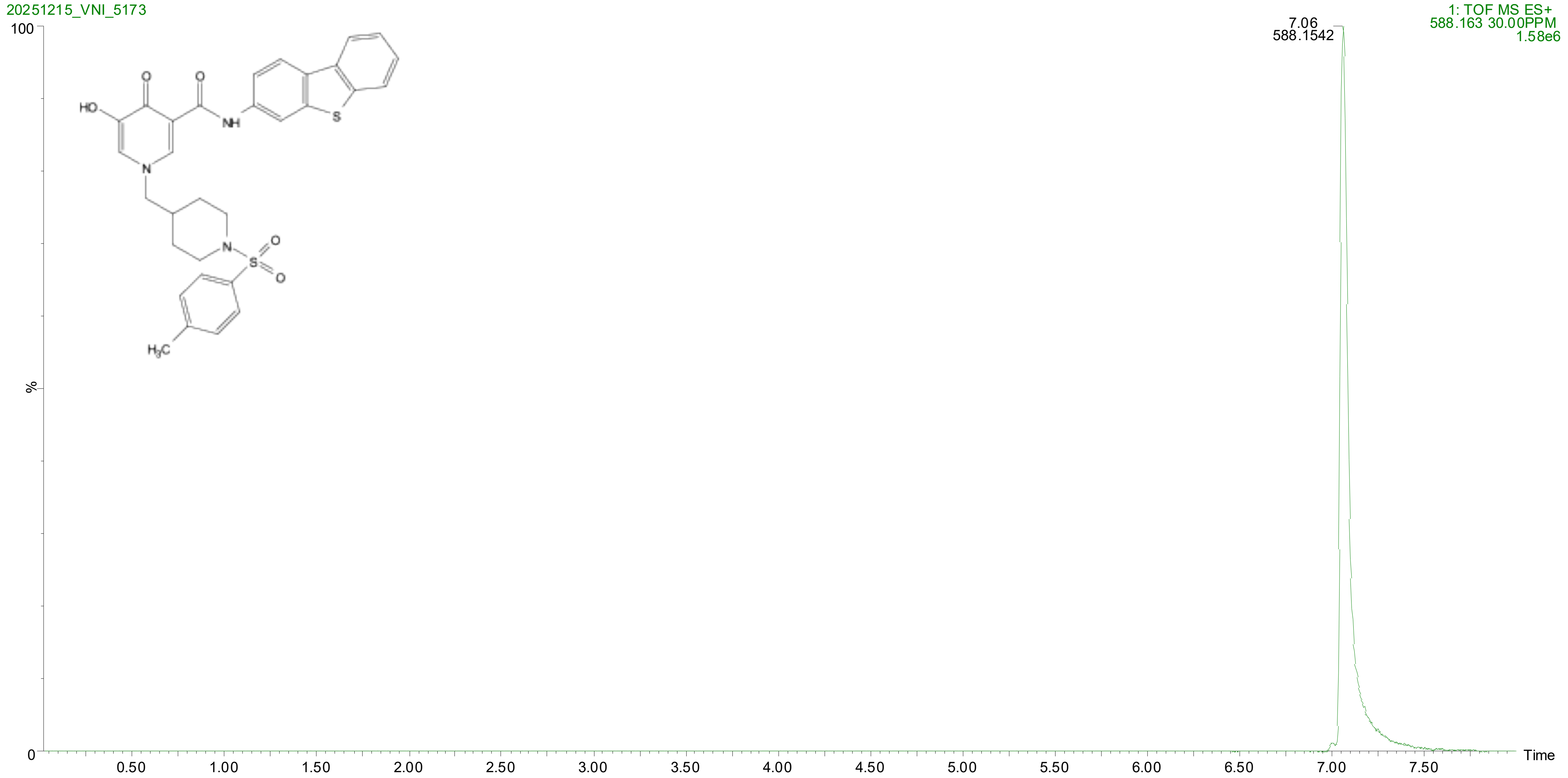

20251215_VNI_5173
1: TOF MS ES+
588.163 30.00PPM
1.58e6
7.06
588.1542
100
%
0
0.50
1.00
1.50
2.00
2.50
3.00
3.50
4.00
4.50
5.00
5.50
6.00
6.50
7.00
7.50
Time

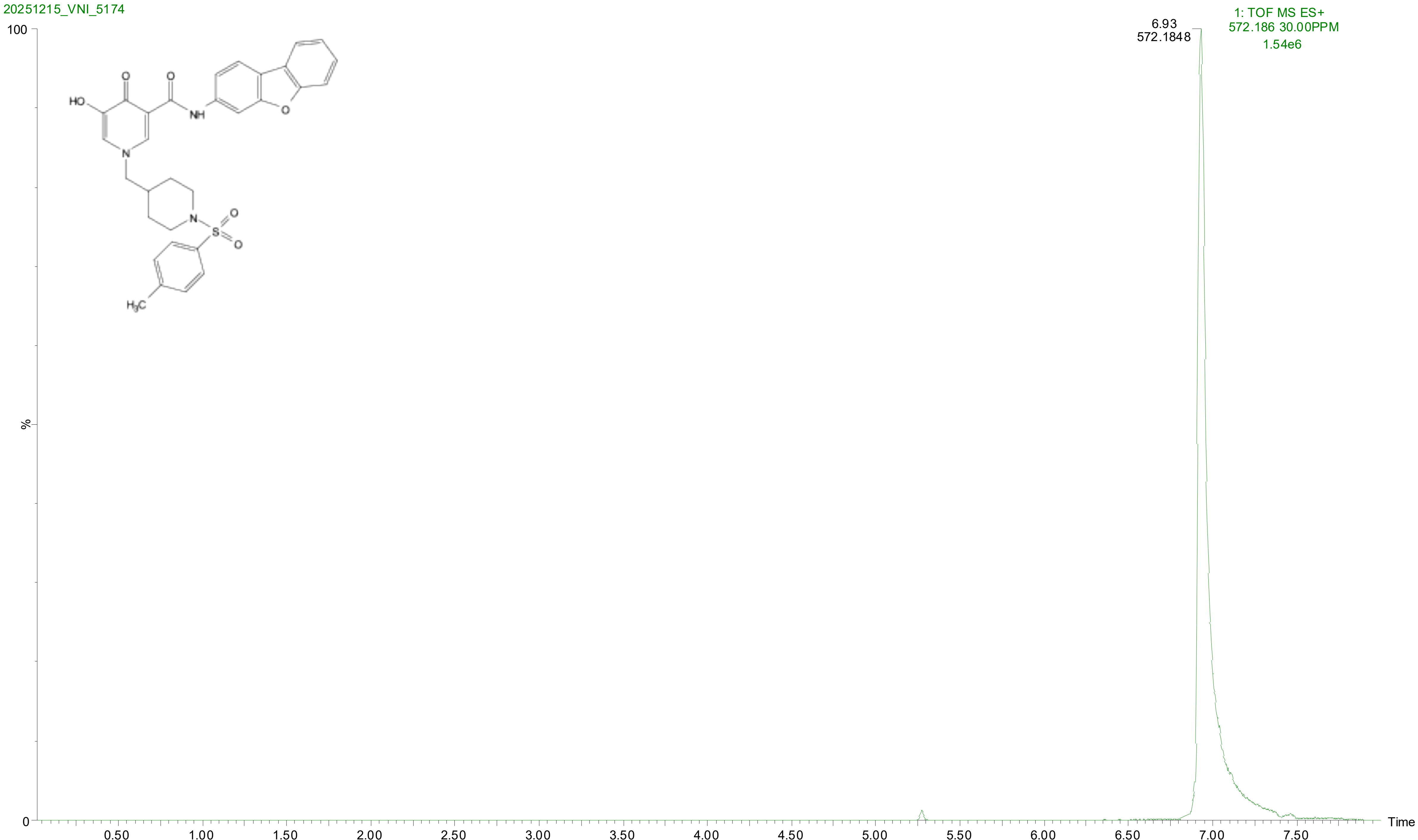
20251215_VNI_5174
1: TOF MS ES+
572.186 30.00PPM
1.54e6
6.93
572.1848
100
%
0
HO
O
O
NH
O
N
N
S
O
O
H3C
0.50
1.00
1.50
2.00
2.50
3.00
3.50
4.00
4.50
5.00
5.50
6.00
6.50
7.00
7.50
Time

HPLC data for compounds

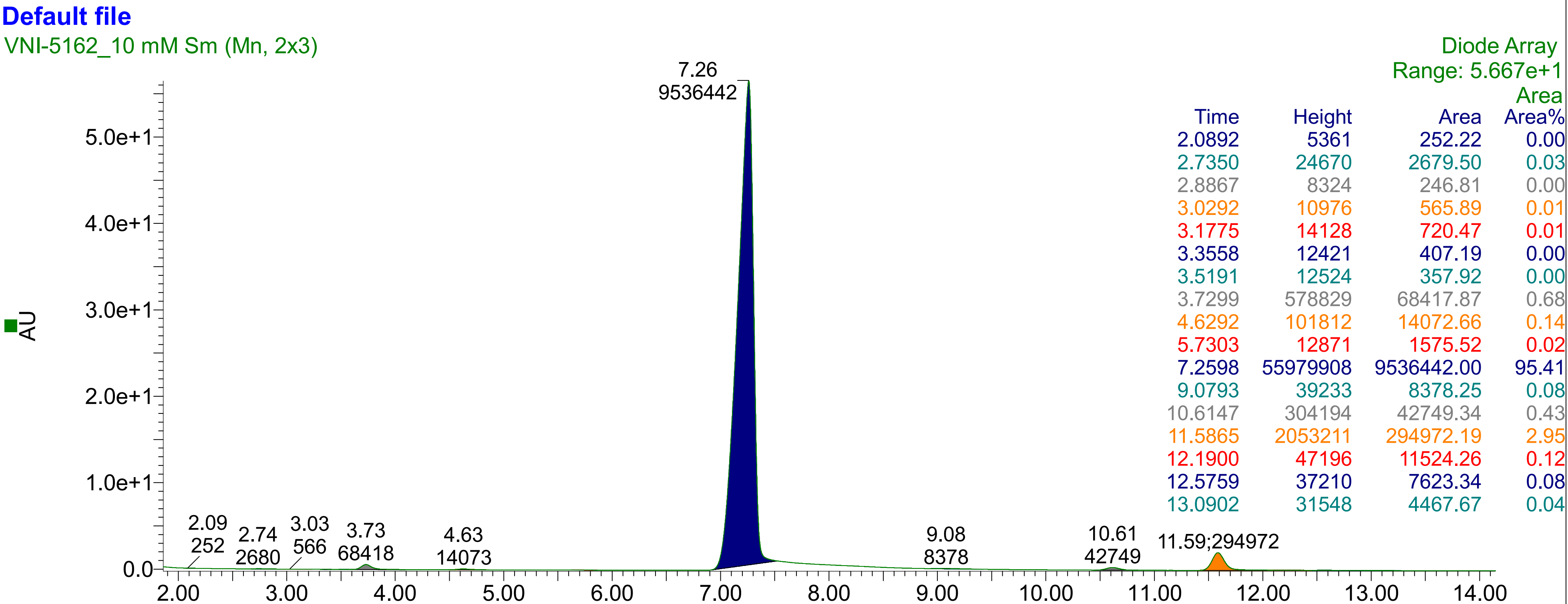

Time	Height	Area	Area Area%
2.0892	5361	252.22	0.00
2.7350	24670	2679.50	0.03
2.8867	8324	246.81	0.00
3.0292	10976	565.89	0.01
3.1775	14128	720.47	0.01
3.3558	12421	407.19	0.00
3.5191	12524	357.92	0.00
3.7299	578829	68417.87	0.68
4.6292	101812	14072.66	0.14
5.7303	12871	1575.52	0.02
7.2598	55979908	9536442.00	95.41
9.0793	39233	8378.25	0.08
10.6147	304194	42749.34	0.43
11.5865	2053211	294972.19	2.95
12.1900	47196	11524.26	0.12
12.5759	37210	7623.34	0.08
13.0902	31548	4467.67	0.04

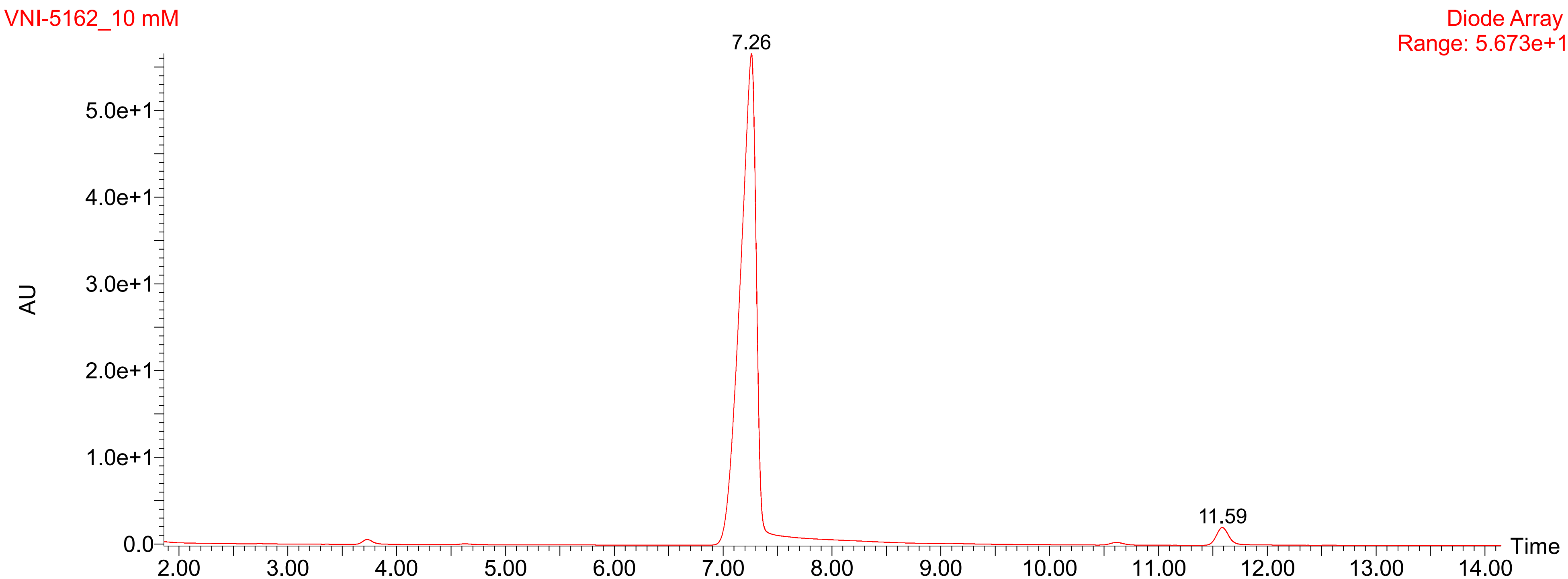

Default file

VNI-5163_10 mM Sm (Mn, 2x3)

Diode Array
Range: 5.245e+1

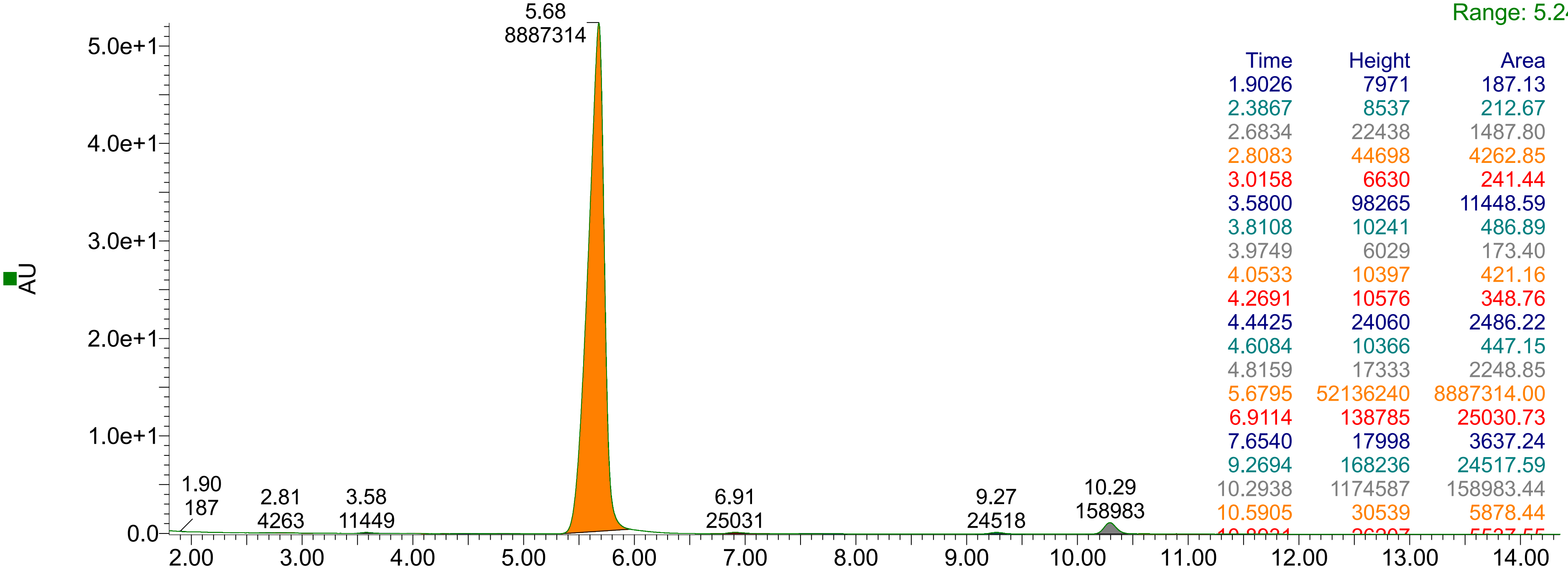

Time	Height	Area	Area Area%
1.9026	7971	187.13	0.00
2.3867	8537	212.67	0.00
2.6834	22438	1487.80	0.02
2.8083	44698	4262.85	0.05
3.0158	6630	241.44	0.00
3.5800	98265	11448.59	0.13
3.8108	10241	486.89	0.01
3.9749	6029	173.40	0.00
4.0533	10397	421.16	0.00
4.2691	10576	348.76	0.00
4.4425	24060	2486.22	0.03
4.6084	10366	447.15	0.00
4.8159	17333	2248.85	0.02
5.6795	52136240	8887314.00	97.27
6.9114	138785	25030.73	0.27
7.6540	17998	3637.24	0.04
9.2694	168236	24517.59	0.27
10.2938	1174587	158983.44	1.74
10.5905	30539	5878.44	0.06
[illegible]	[illegible]	[illegible]	[illegible]

VNI-5163_10 mM

Diode Array
Range: 5.25e+1

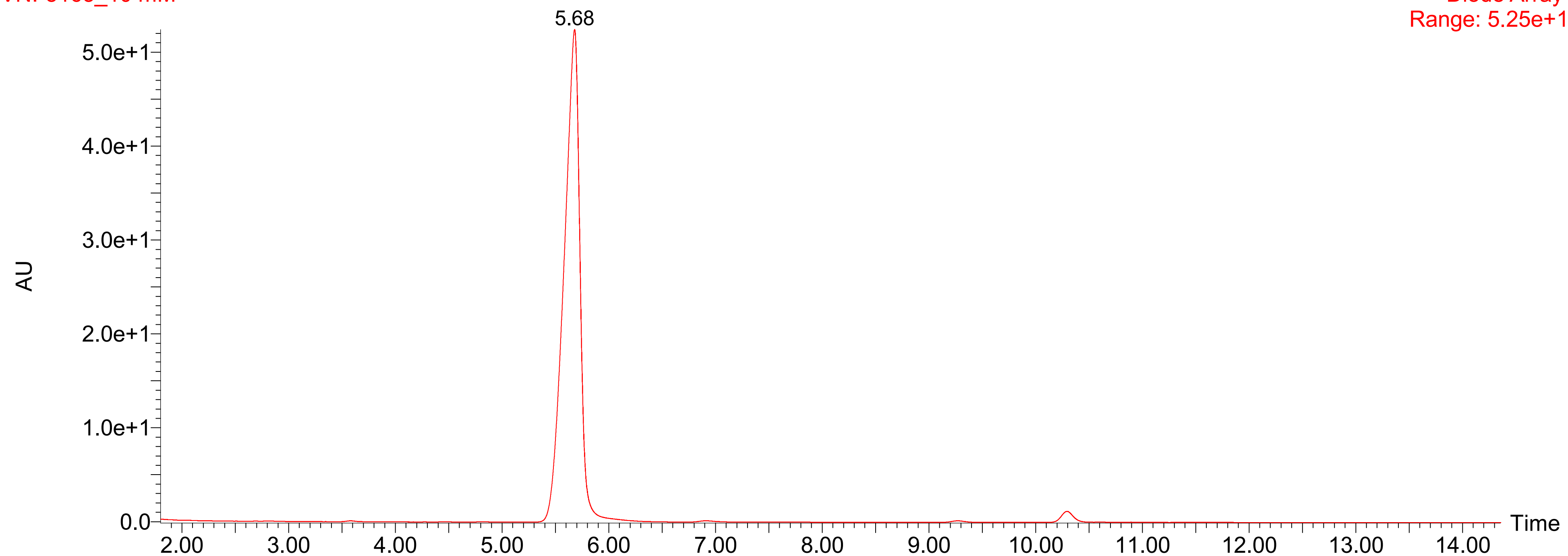

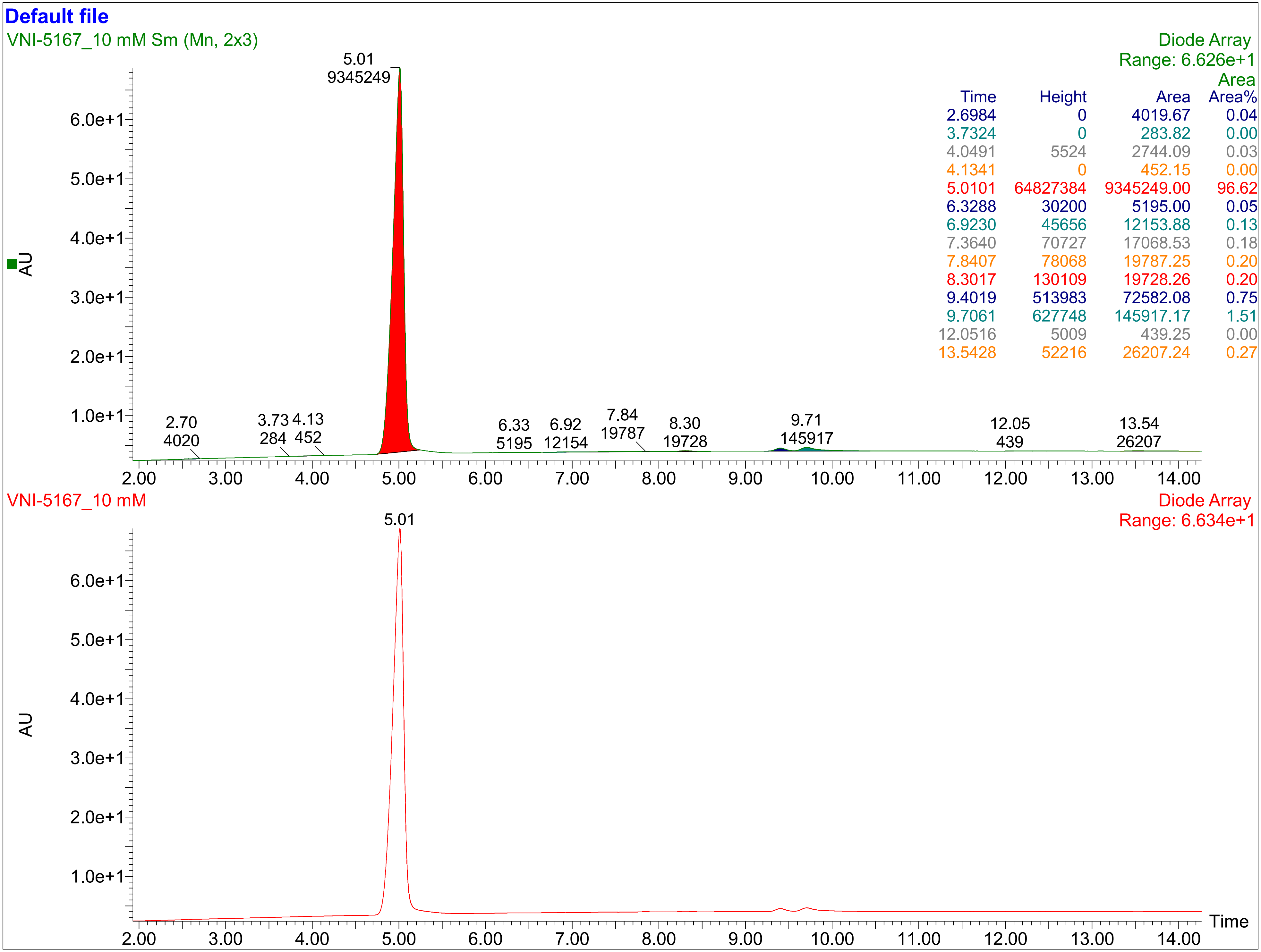

Time	Height	Area	Area%
2.6984	0	4019.67	0.04
3.7324	0	283.82	0.00
4.0491	5524	2744.09	0.03
4.1341	0	452.15	0.00
5.0101	64827384	9345249.00	96.62
6.3288	30200	5195.00	0.05
6.9230	45656	12153.88	0.13
7.3640	70727	17068.53	0.18
7.8407	78068	19787.25	0.20
8.3017	130109	19728.26	0.20
9.4019	513983	72582.08	0.75
9.7061	627748	145917.17	1.51
12.0516	5009	439.25	0.00
13.5428	52216	26207.24	0.27

Default file

VNI-5168_10 mM Sm (Mn, 2x3)

Diode Array
Range: 3.933e+1

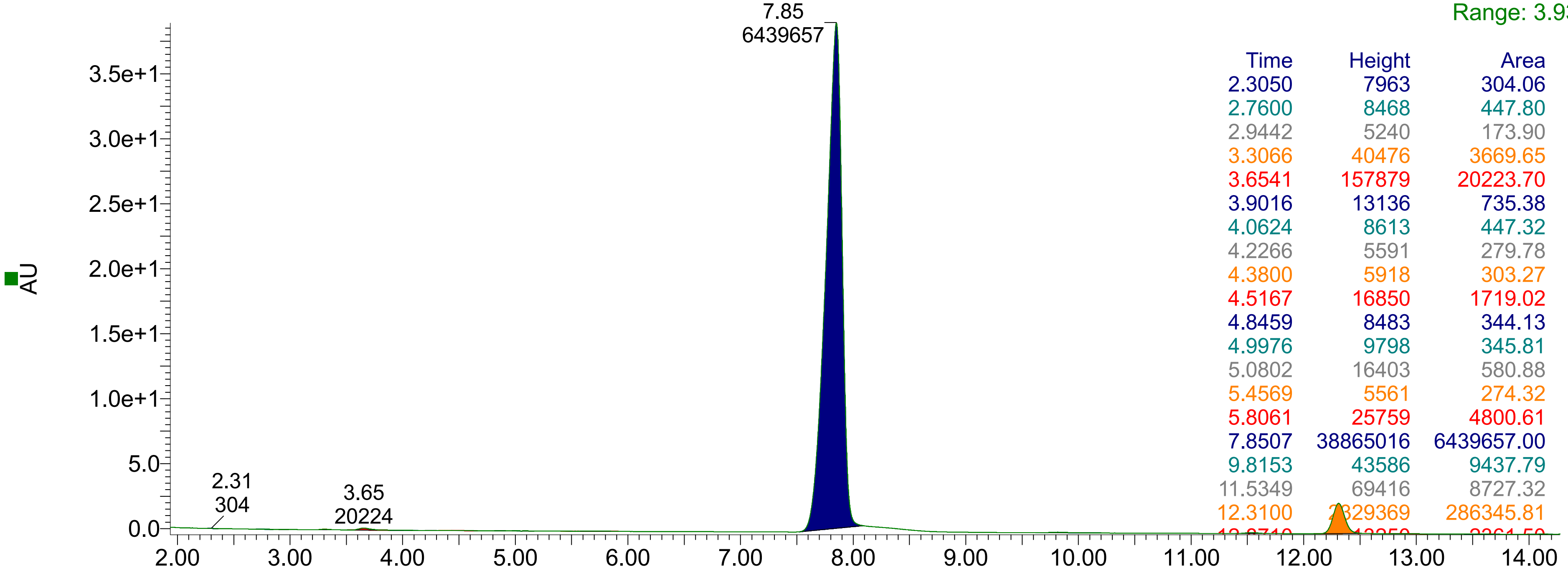

Time	Height	Area	Area Area%
2.3050	7963	304.06	0.00
2.7600	8468	447.80	0.01
2.9442	5240	173.90	0.00
3.3066	40476	3669.65	0.05
3.6541	157879	20223.70	0.30
3.9016	13136	735.38	0.01
4.0624	8613	447.32	0.01
4.2266	5591	279.78	0.00
4.3800	5918	303.27	0.00
4.5167	16850	1719.02	0.03
4.8459	8483	344.13	0.01
4.9976	9798	345.81	0.01
5.0802	16403	580.88	0.01
5.4569	5561	274.32	0.00
5.8061	25759	4800.61	0.07
7.8507	38865016	6439657.00	94.91
9.8153	43586	9437.79	0.14
11.5349	69416	8727.32	0.13
12.3100	2329369	286345.81	4.22
[illegible]	[illegible]	[illegible]	[illegible]

VNI-5168_10 mM

Diode Array
Range: 3.937e+1

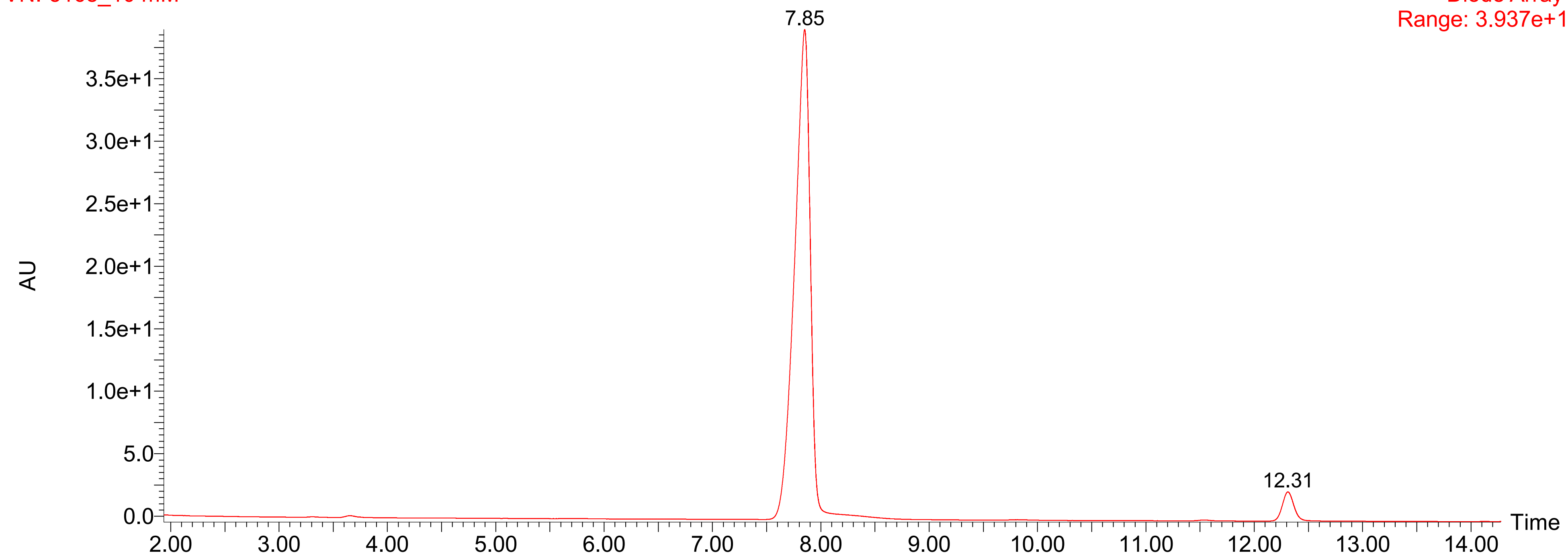

^{1}H NMR and ^{13}C NMR spectra

2

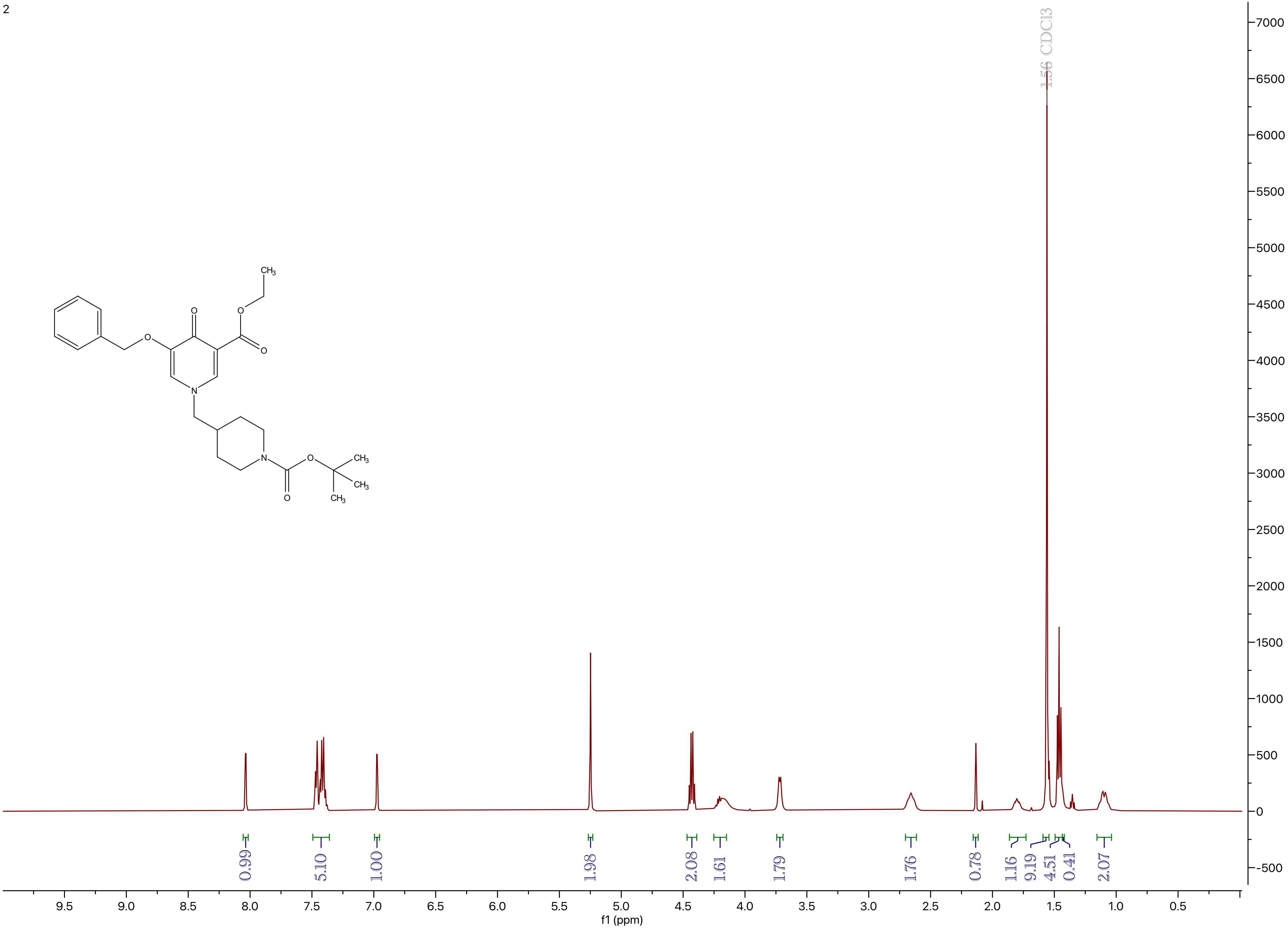

1.56 CDCl3
0.99
5.10
1.00
1.98
2.08
1.61
1.79
1.76
0.78
1.16
9.19
4.51
0.41
2.07
9.5
9.0
8.5
8.0
7.5
7.0
6.5
6.0
5.5
5.0
4.5
4.0
3.5
3.0
2.5
2.0
1.5
1.0
0.5
f1 (ppm)
7000
6500
6000
5500
5000
4500
4000
3500
3000
2500
2000
1500
1000
500
0
-500
CH3
O
O
O
O
N
N
O
O
CH3
CH3
CH3

3

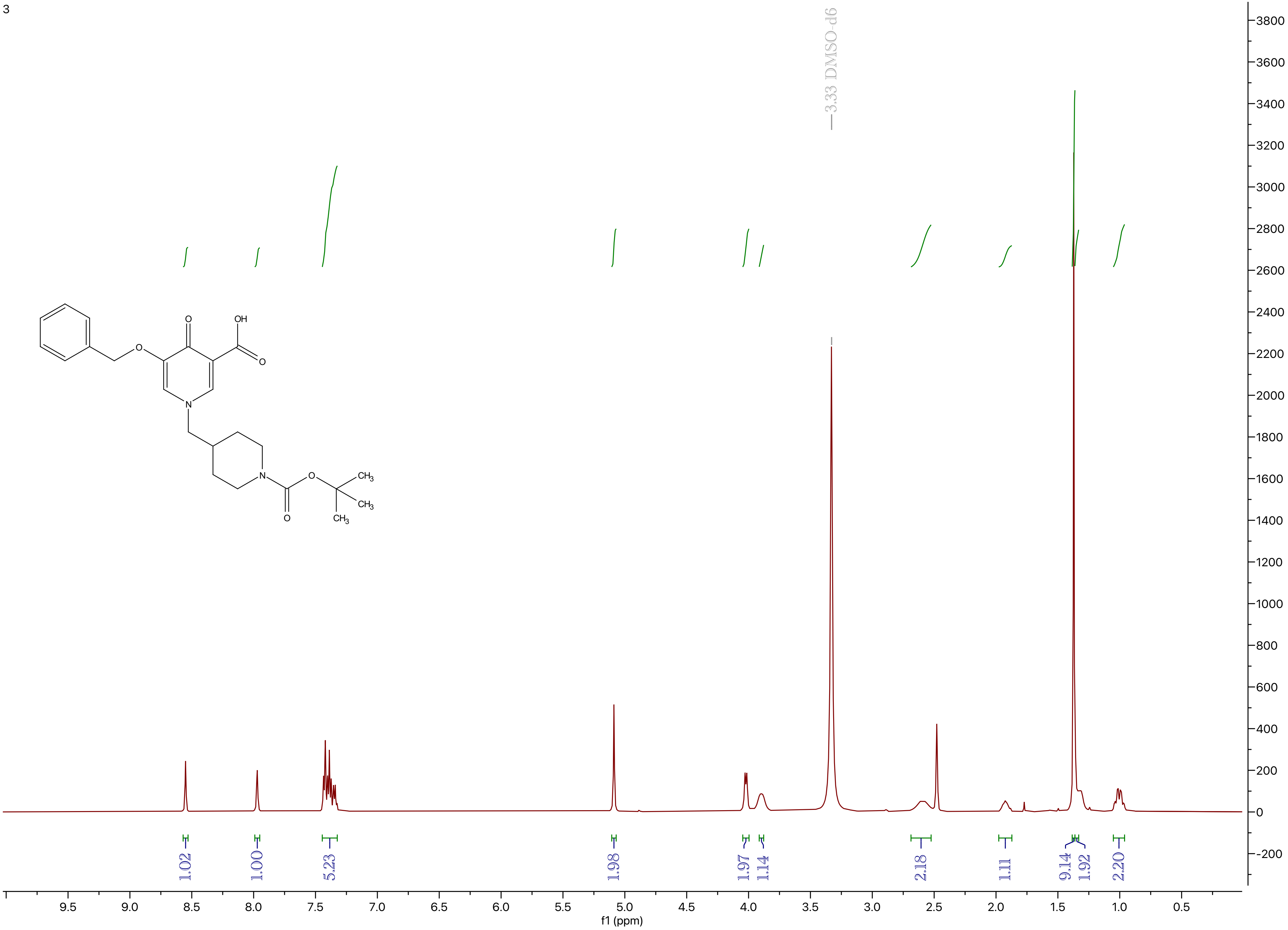

3.33 DMSO-d6
1.02
1.00
5.23
1.98
1.97
1.14
2.18
1.11
9.14
1.92
2.20
9.5
9.0
8.5
8.0
7.5
7.0
6.5
6.0
5.5
5.0
4.5
4.0
3.5
3.0
2.5
2.0
1.5
1.0
0.5
f1 (ppm)
3800
3600
3400
3200
3000
2800
2600
2400
2200
2000
1800
1600
1400
1200
1000
800
600
400
200
0
-200
O
OH
O
O
N
N
O
O
CH3
CH3
CH3

4

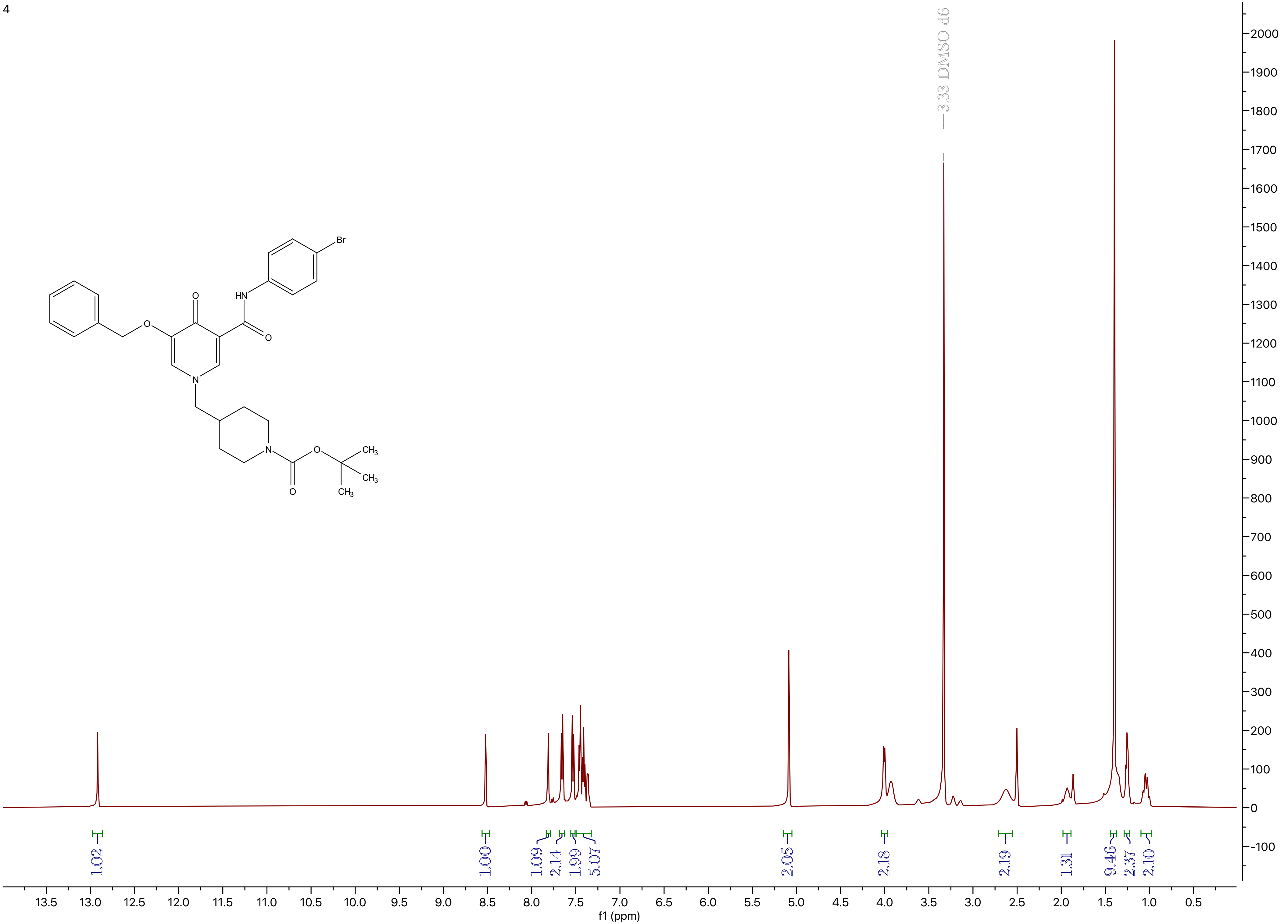
3.33 DMSO-d6
Br
HN
O
O
O
N
N
O
O
CH3
CH3
CH3
1.02
1.00
1.09
2.14
1.99
5.07
2.05
2.18
2.19
1.31
9.46
2.37
2.10
13.5
13.0
12.5
12.0
11.5
11.0
10.5
10.0
9.5
9.0
8.5
8.0
7.5
7.0
6.5
6.0
5.5
5.0
4.5
4.0
3.5
3.0
2.5
2.0
1.5
1.0
0.5
f1 (ppm)
2000
1900
1800
1700
1600
1500
1400
1300
1200
1100
1000
900
800
700
600
500
400
300
200
100
0
-100

5

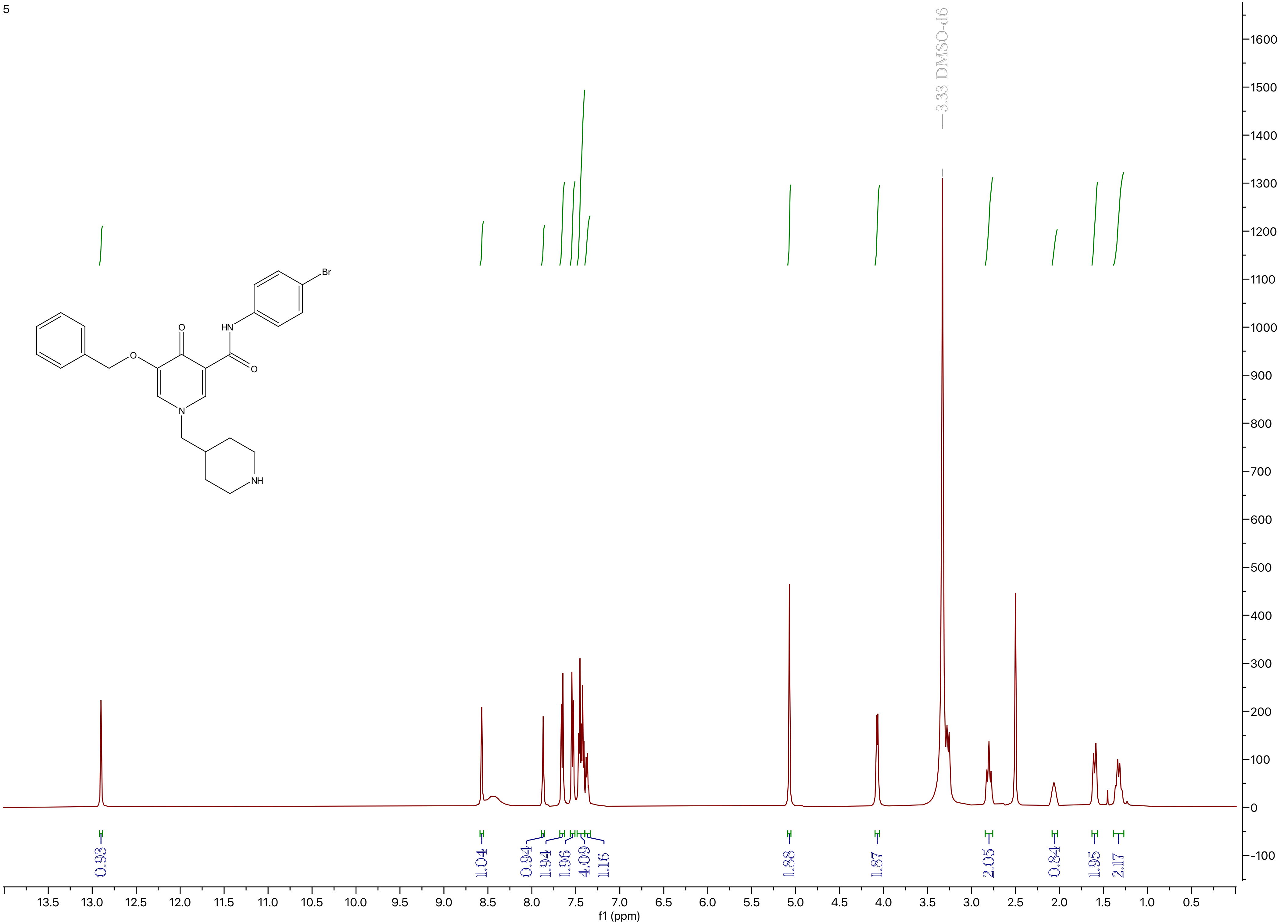
3.33 DMSO-d6
Br
HN
O
O
O
N
NH
0.93
1.04
0.94
1.94
1.96
4.09
1.16
1.88
1.87
2.05
0.84
1.95
2.17
f1 (ppm)

VNI-5158

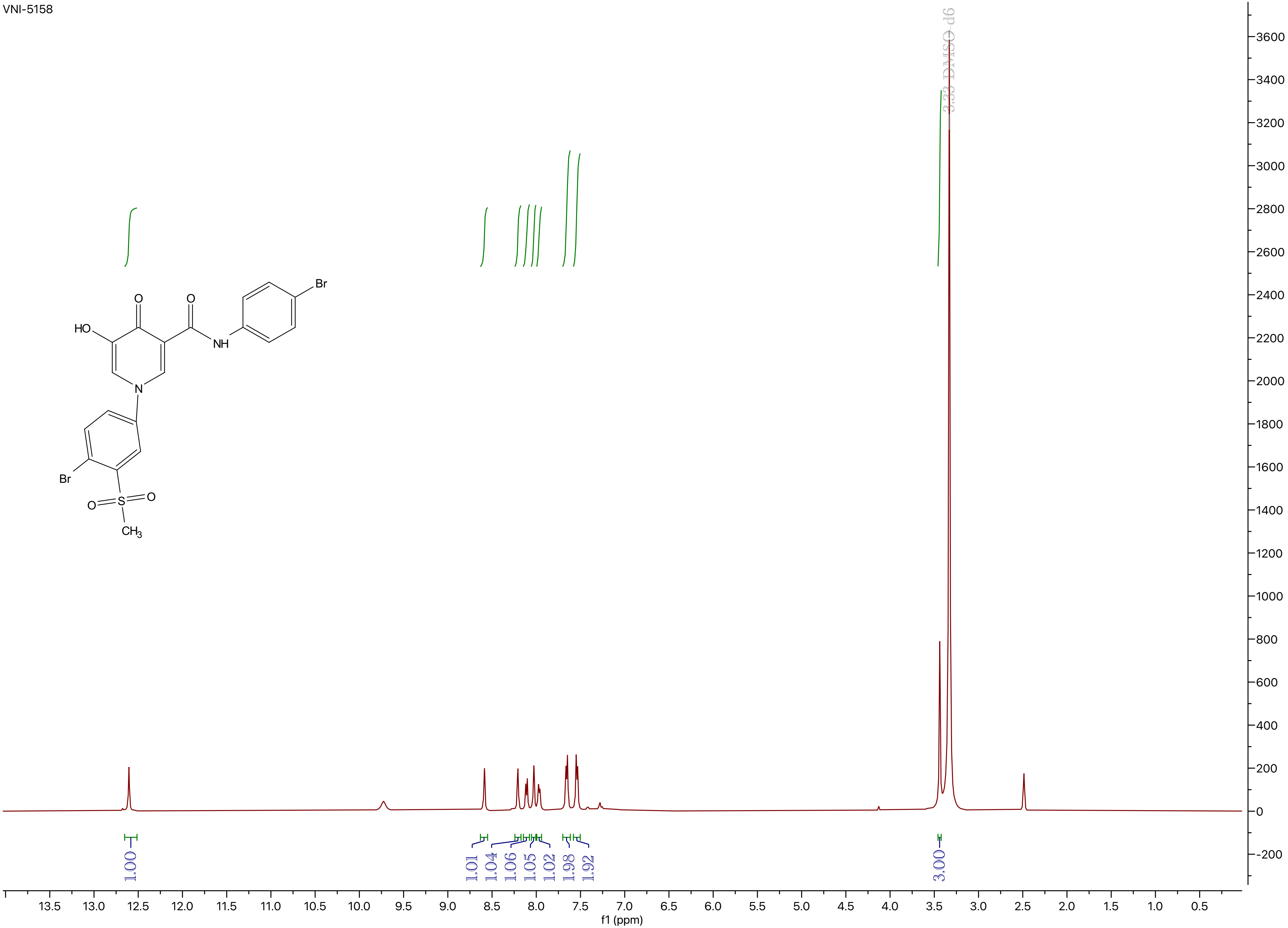

3.33 DMSO-d6
HO
O
O
NH
Br
N
Br
O
S
O
CH3
1.00
1.01
1.04
1.06
1.05
1.02
1.98
1.92
3.00
13.5 13.0 12.5 12.0 11.5 11.0 10.5 10.0 9.5 9.0 8.5 8.0 7.5 7.0 6.5 6.0 5.5 5.0 4.5 4.0 3.5 3.0 2.5 2.0 1.5 1.0 0.5
f1 (ppm)
3600 3400 3200 3000 2800 2600 2400 2200 2000 1800 1600 1400 1200 1000 800 600 400 200 0 -200

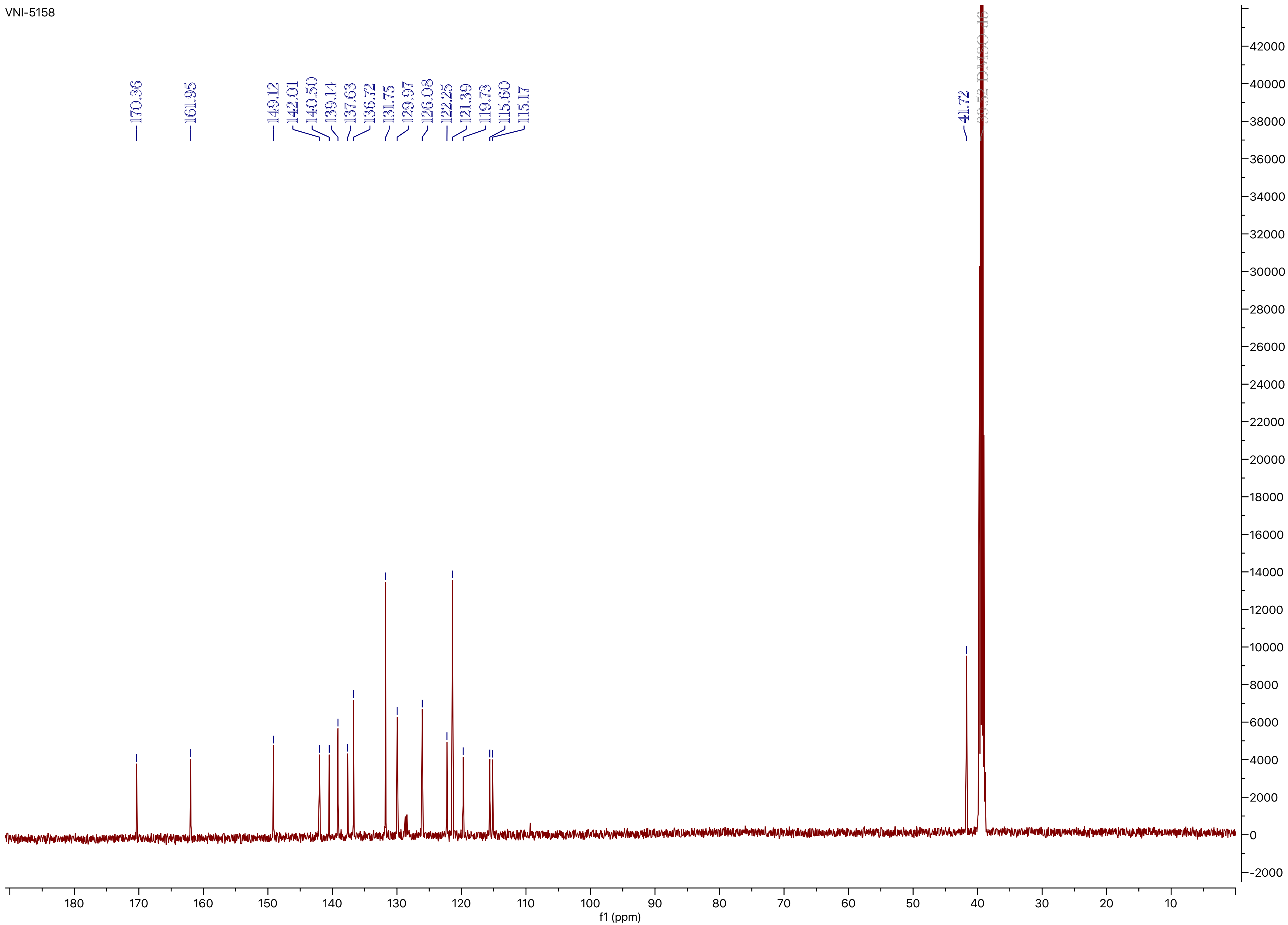

VNI-5158
170.36
161.95
149.12
142.01
140.50
139.14
137.63
136.72
131.75
129.97
126.08
122.25
121.39
119.73
115.60
115.17
41.72
39.52 DMSO-d6
f1 (ppm)

VNI-5159

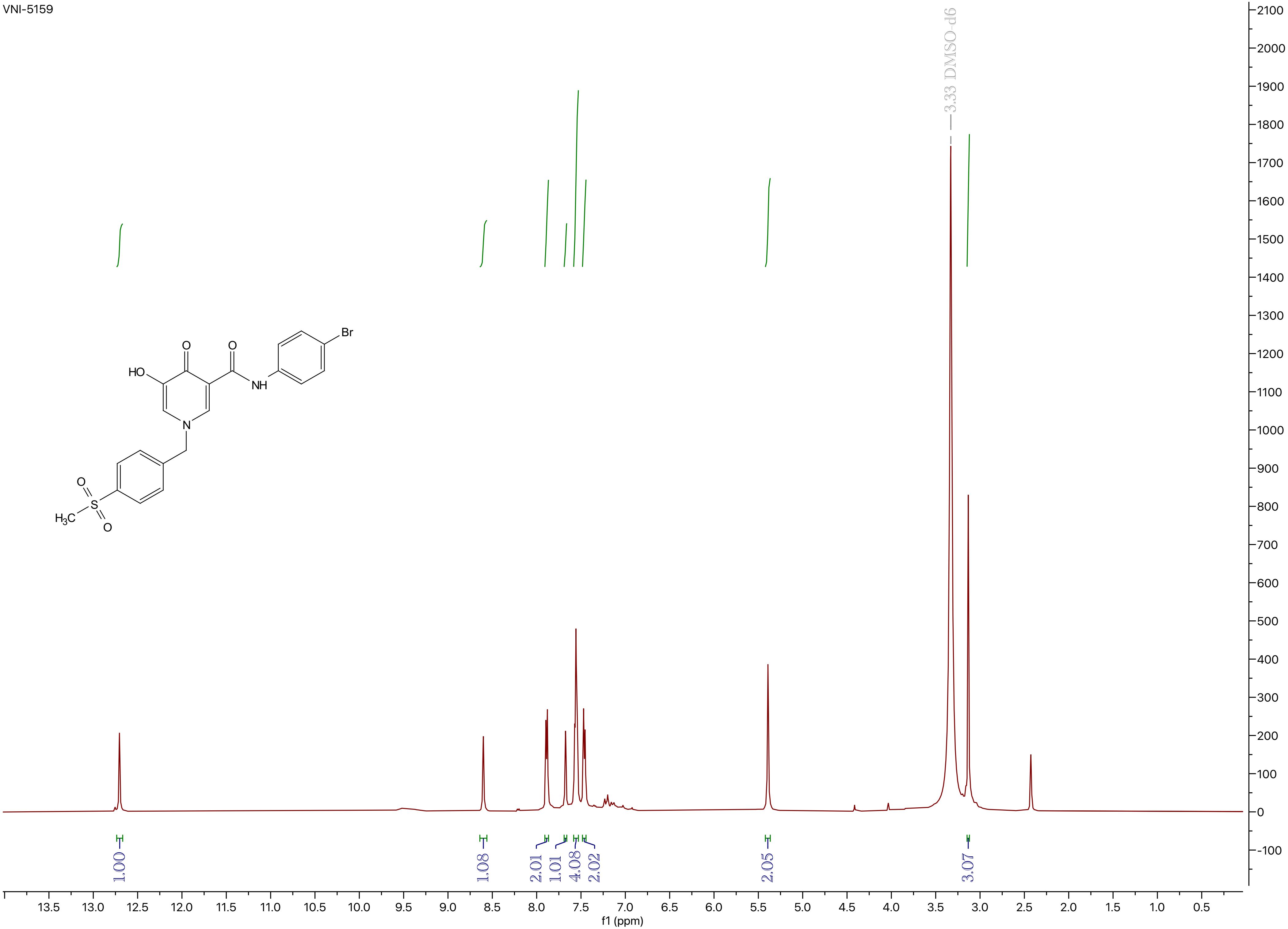

3.33 DMSO-d6
HO
O
O
NH
Br
N
O
S
O
H3C
1.00
1.08
2.01
1.01
4.08
2.02
2.05
3.07
13.5
13.0
12.5
12.0
11.5
11.0
10.5
10.0
9.5
9.0
8.5
8.0
7.5
7.0
6.5
6.0
5.5
5.0
4.5
4.0
3.5
3.0
2.5
2.0
1.5
1.0
0.5
f1 (ppm)
2100
2000
1900
1800
1700
1600
1500
1400
1300
1200
1100
1000
900
800
700
600
500
400
300
200
100
0
-100

VNI-5159

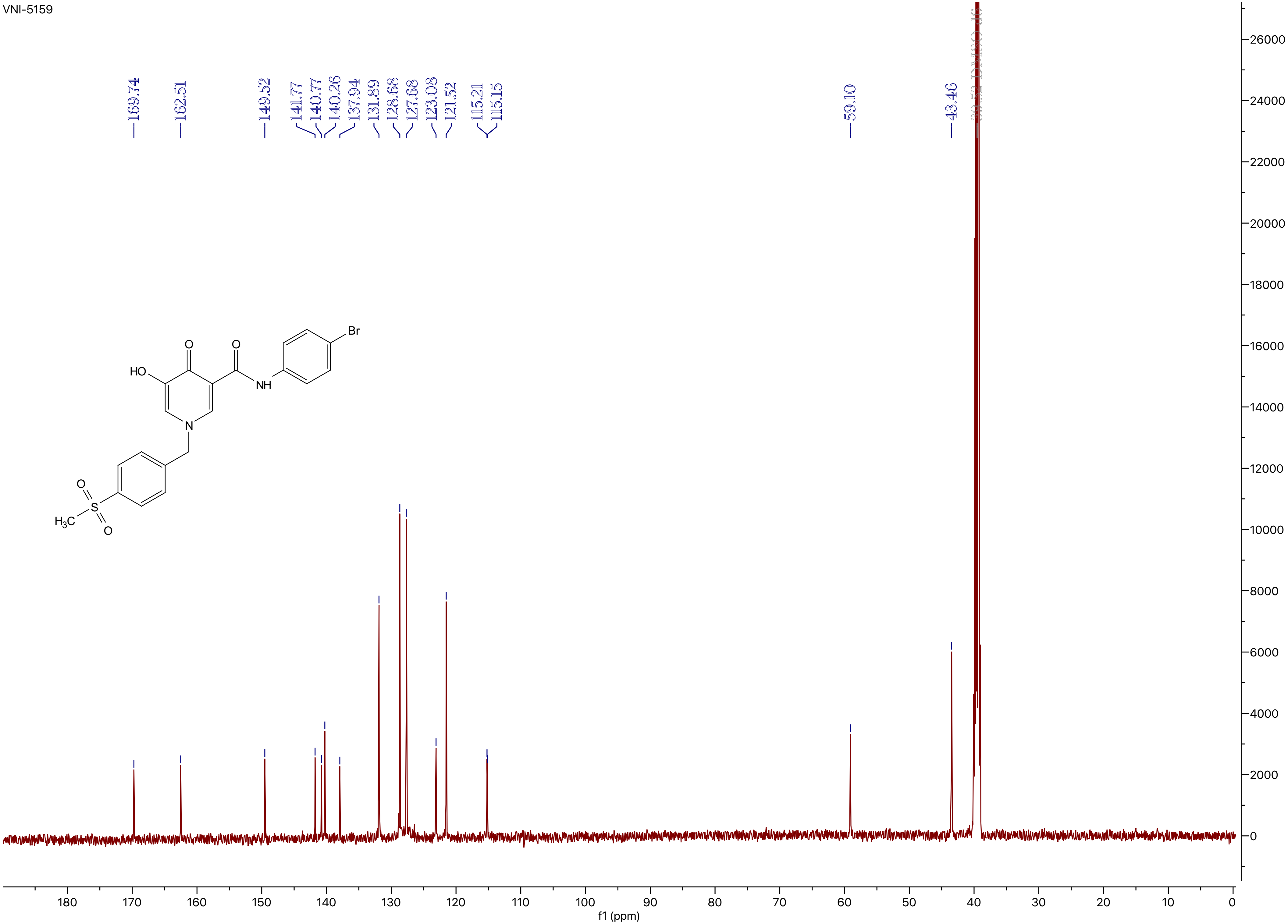

169.74
162.51
149.52
141.77
140.77
140.26
137.94
131.89
128.68
127.68
123.08
121.52
115.21
115.15
59.10
43.46
39.52 DMSO-d6
HO
O
O
NH
Br
N
O
S
O
H3C
f1 (ppm)

VNI-5160

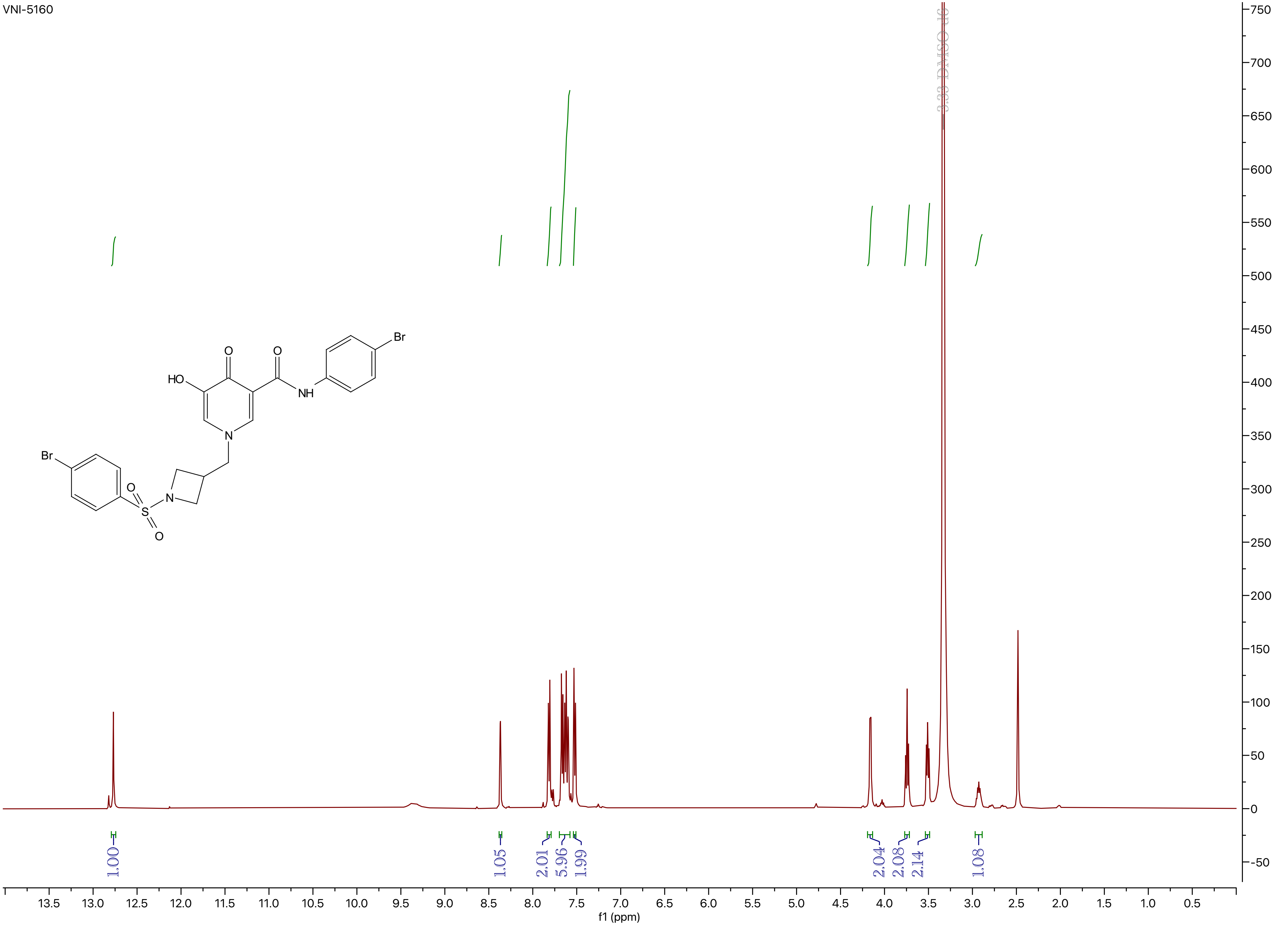

3.33 DMSO-d6
HO
O
O
NH
Br
N
Br
O
S
N
O
1.00
1.05
2.01
5.96
1.99
2.04
2.08
2.14
1.08
13.5 13.0 12.5 12.0 11.5 11.0 10.5 10.0 9.5 9.0 8.5 8.0 7.5 7.0 6.5 6.0 5.5 5.0 4.5 4.0 3.5 3.0 2.5 2.0 1.5 1.0 0.5
f1 (ppm)
750 700 650 600 550 500 450 400 350 300 250 200 150 100 50 0 -50

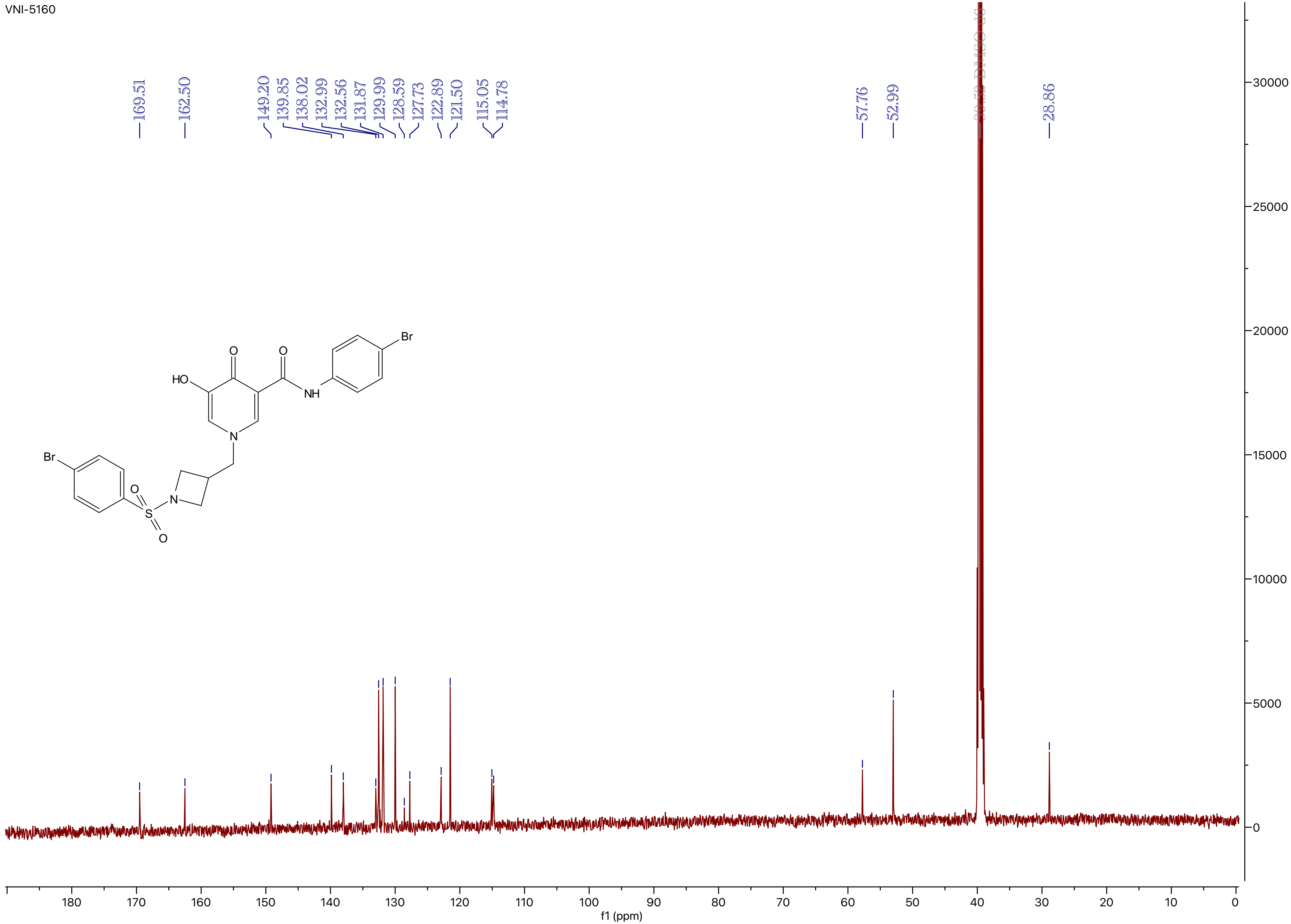
VNI-5160
169.51
162.50
149.20
139.85
138.02
132.99
132.56
131.87
129.99
128.59
127.73
122.89
121.50
115.05
114.78
57.76
52.99
39.52 DMSO-d6
28.86
Br
O
O
HO
NH
N
Br
O
S
N
O
30000
25000
20000
15000
10000
5000
0
180
170
160
150
140
130
120
110
100
90
80
70
60
50
40
30
20
10
0
f1 (ppm)

VNI-5161

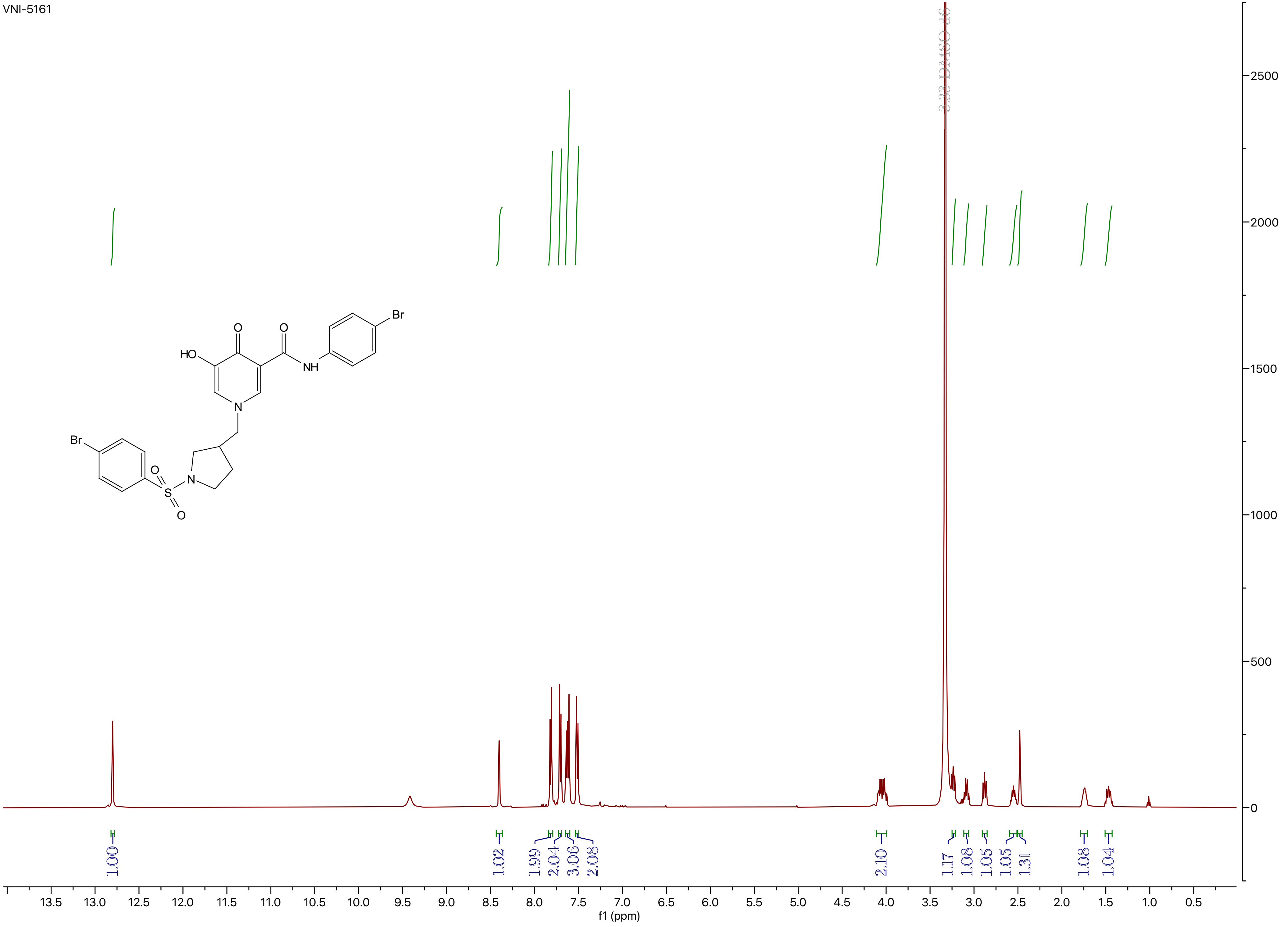
3.33 DMSO-d6
HO
O
O
NH
Br
N
Br
O
S
O
N
1.00
1.02
1.99
2.04
3.06
2.08
2.10
1.17
1.08
1.05
1.05
1.31
1.08
1.04
2500
2000
1500
1000
500
0
13.5 13.0 12.5 12.0 11.5 11.0 10.5 10.0 9.5 9.0 8.5 8.0 7.5 7.0 6.5 6.0 5.5 5.0 4.5 4.0 3.5 3.0 2.5 2.0 1.5 1.0 0.5
f1 (ppm)

VNI-5161

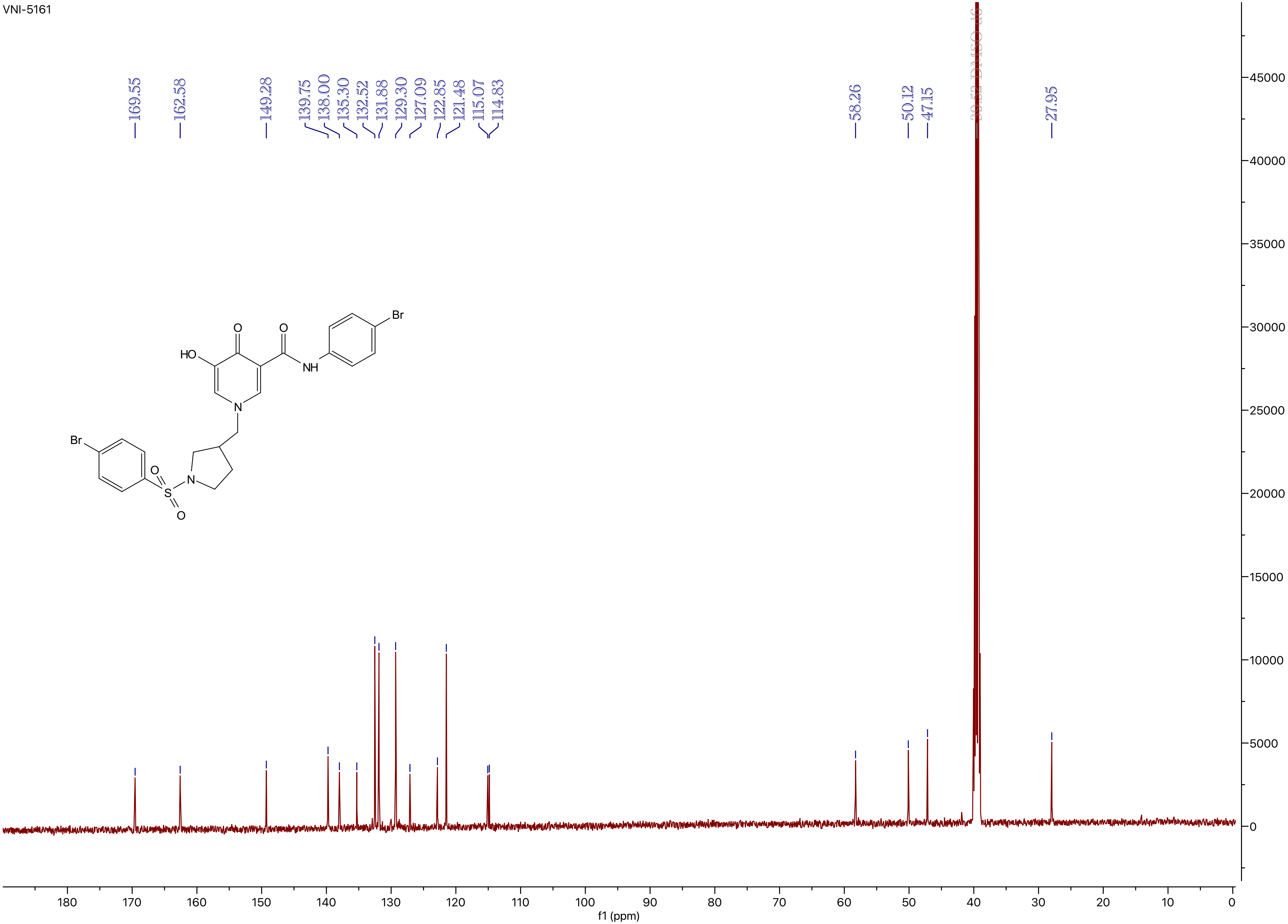
169.55
162.58
149.28
139.75
138.00
135.30
132.52
131.88
129.30
127.09
122.85
121.48
115.07
114.83
58.26
50.12
47.15
39.52 DMSO-d6
27.95
HO
O
O
NH
Br
N
Br
O
S
O
N
f1 (ppm)
45000
40000
35000
30000
25000
20000
15000
10000
5000
0
180
170
160
150
140
130
120
110
100
90
80
70
60
50
40
30
20
10
0

VNI-5162

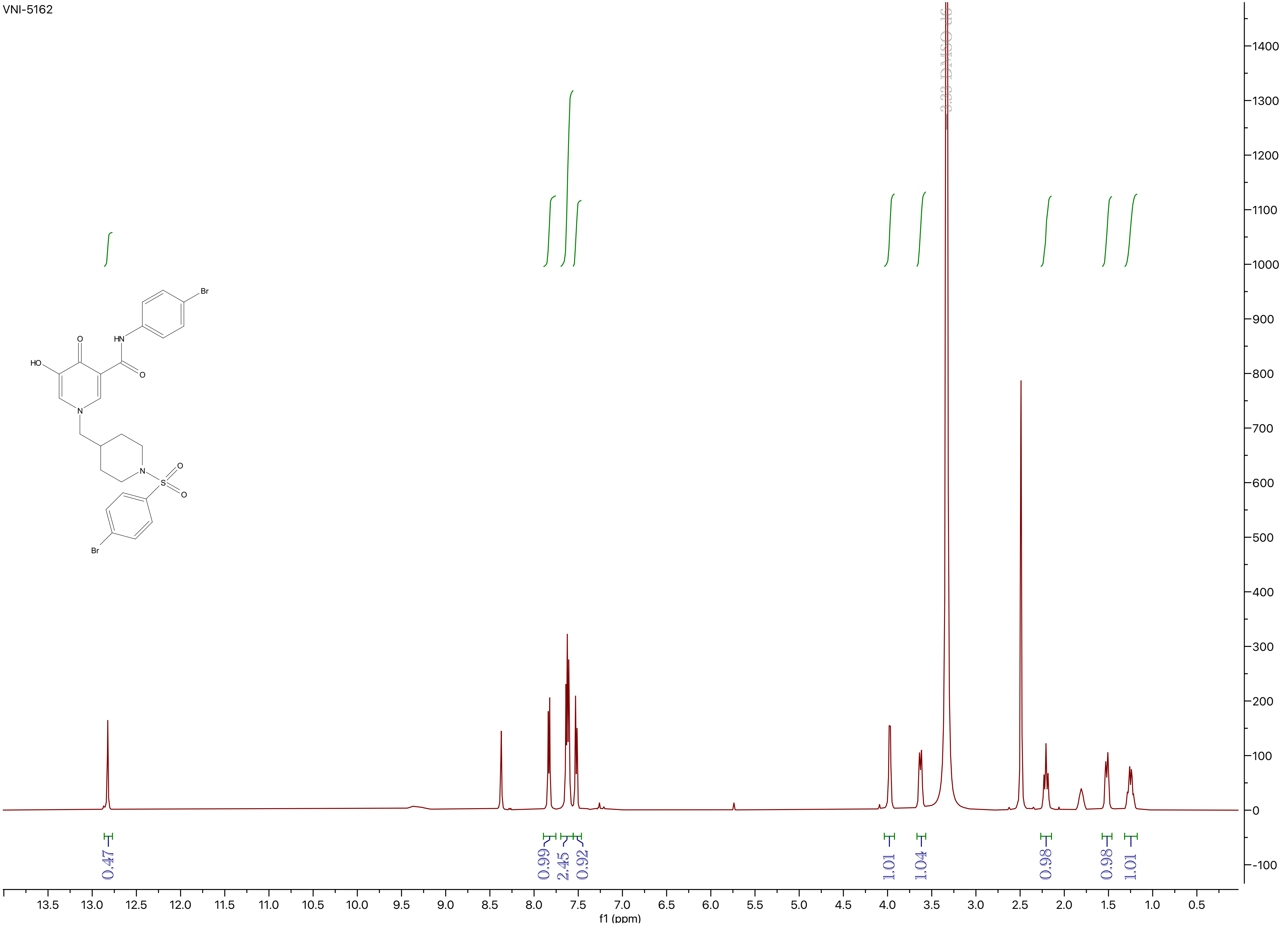

VNI-5162

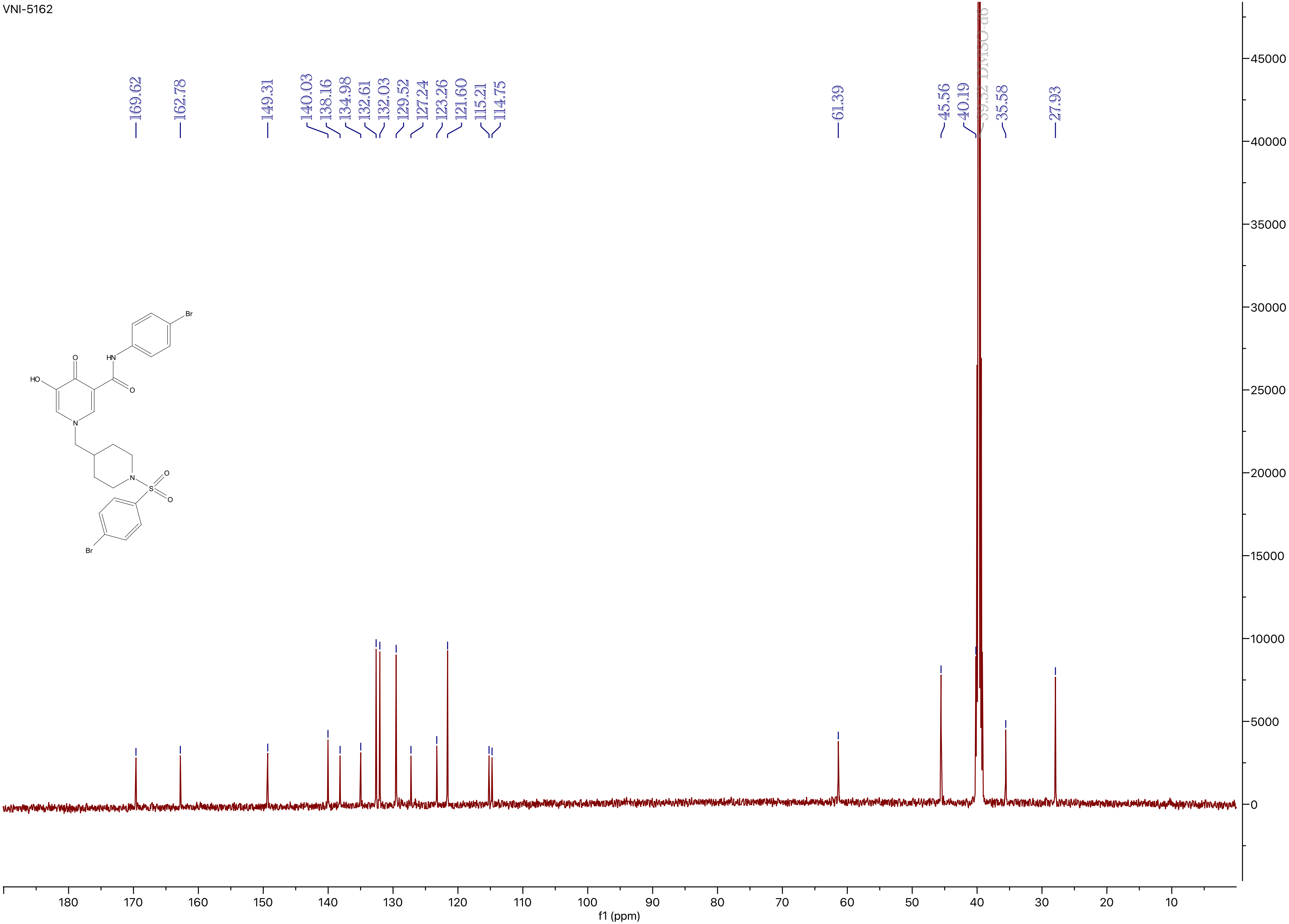

169.62
162.78
149.31
140.03
138.16
134.98
132.61
132.03
129.52
127.24
123.26
121.60
115.21
114.75
61.39
45.56
40.19
39.52 DMSO-d6
35.58
27.93
Br
HN
O
HO
O
N
N
S
O
O
Br
45000
40000
35000
30000
25000
20000
15000
10000
5000
0
180
170
160
150
140
130
120
110
100
90
80
70
60
50
40
30
20
10
f1 (ppm)

VNI-5163

^{1}H NMR (500 MHz, DMSO-d_6) δ 2.410 (s, 1H).

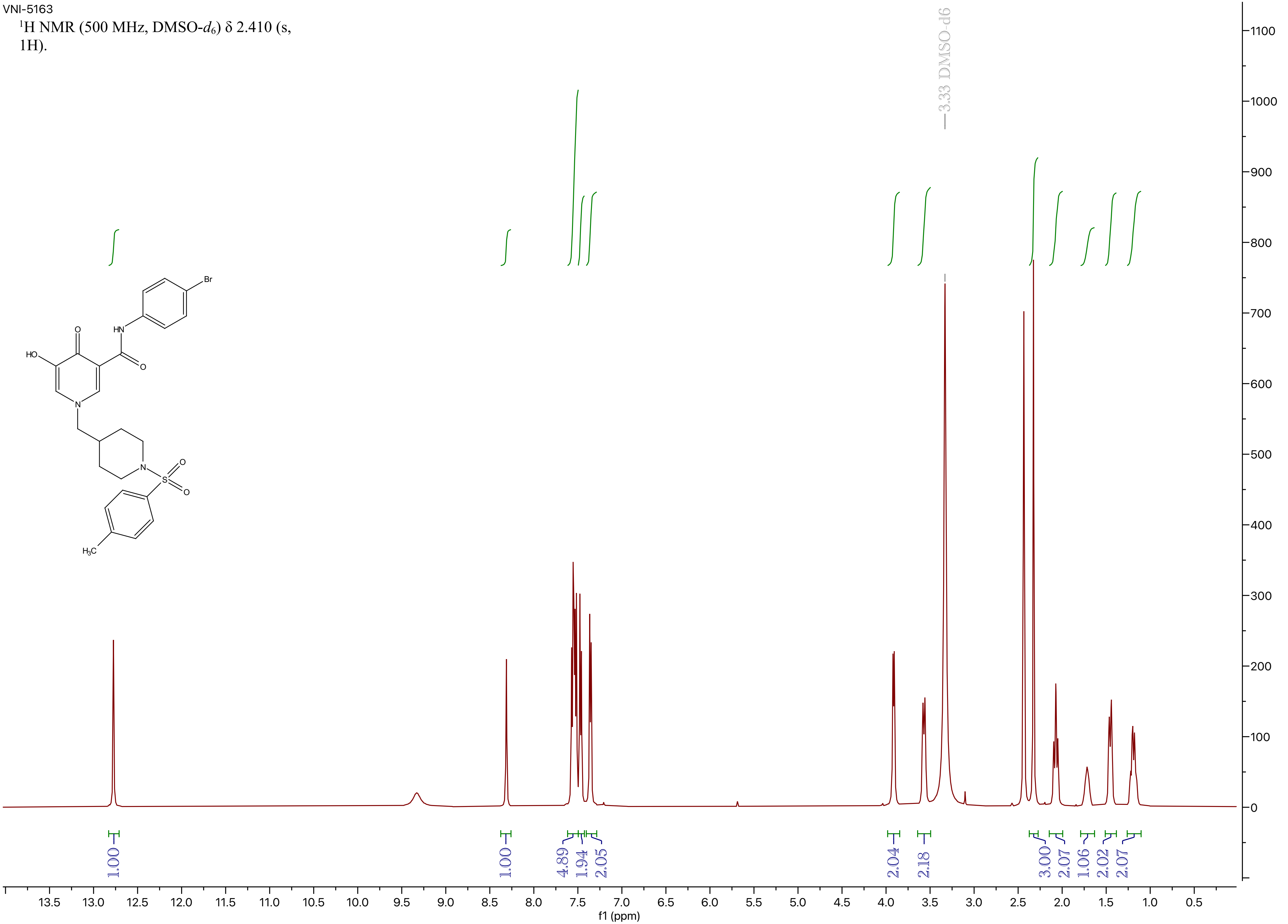

VNI-5163

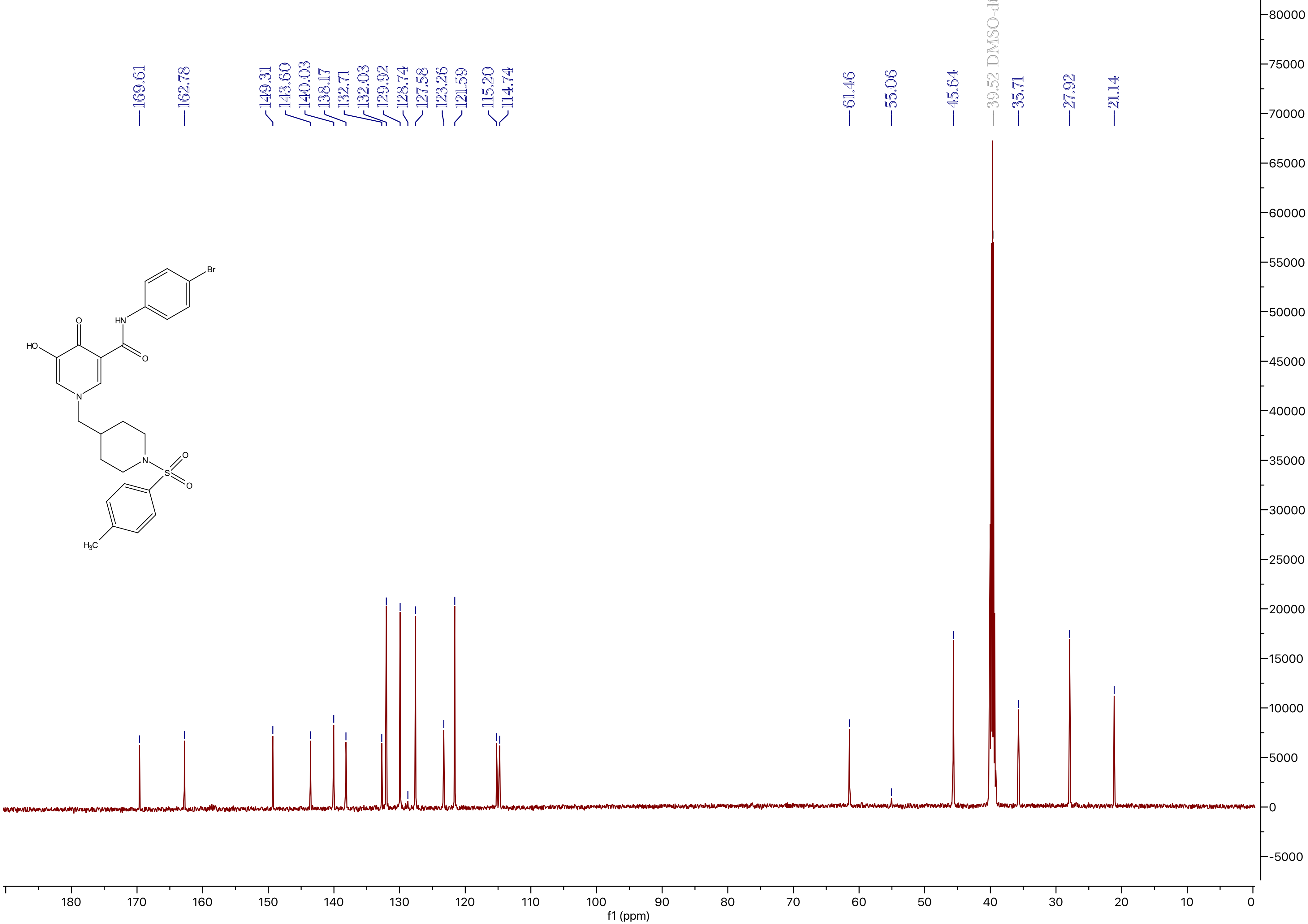

169.61
162.78
149.31
143.60
140.03
138.17
132.71
132.03
129.92
128.74
127.58
123.26
121.59
115.20
114.74
61.46
55.06
45.64
39.52 DMSO-d6
35.71
27.92
21.14
Br
HN
O
HO
O
N
N
S
O
O
H3C
80000
75000
70000
65000
60000
55000
50000
45000
40000
35000
30000
25000
20000
15000
10000
5000
0
-5000
180
170
160
150
140
130
120
110
100
90
80
70
60
50
40
30
20
10
0
f1 (ppm)

VNI-5164

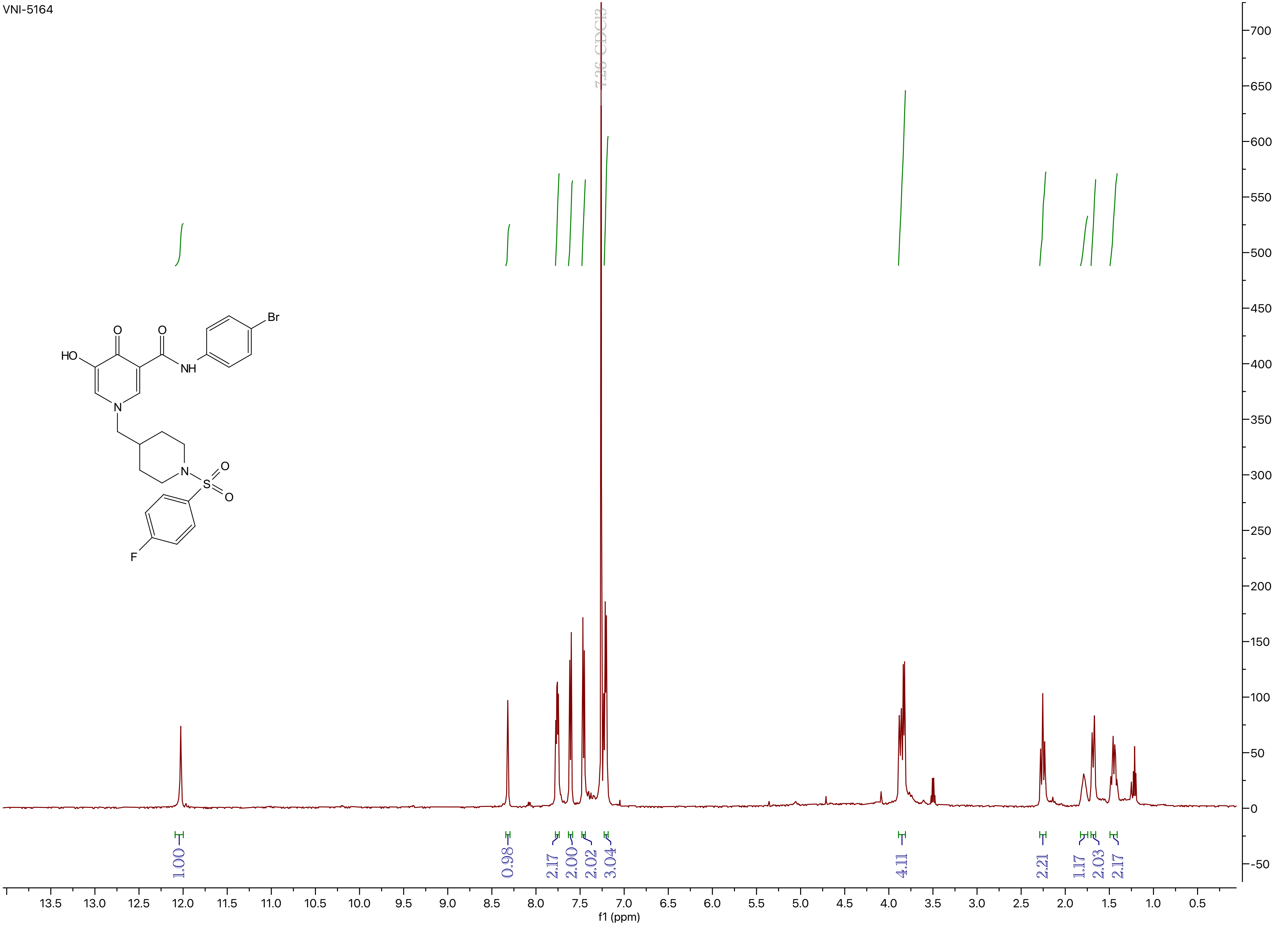

7.26 CDCl3
HO
O
O
NH
Br
N
N
S
O
O
F
1.00
0.98
2.17
2.00
2.02
3.04
4.11
2.21
1.17
2.03
2.17
13.5
13.0
12.5
12.0
11.5
11.0
10.5
10.0
9.5
9.0
8.5
8.0
7.5
7.0
6.5
6.0
5.5
5.0
4.5
4.0
3.5
3.0
2.5
2.0
1.5
1.0
0.5
f1 (ppm)
700
650
600
550
500
450
400
350
300
250
200
150
100
50
0
-50

VNI-5164

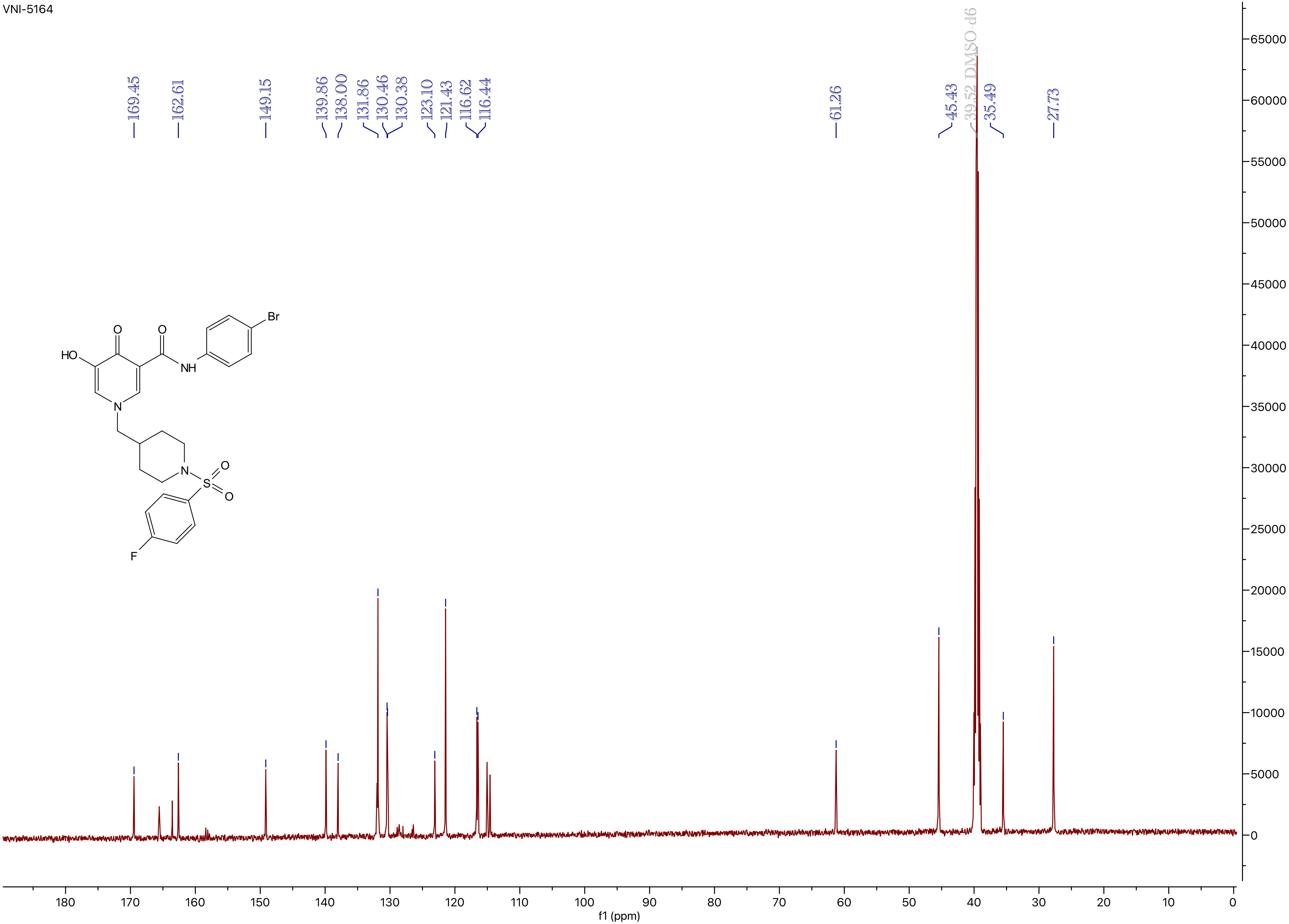
169.45
162.61
149.15
139.86
138.00
131.86
130.46
130.38
123.10
121.43
116.62
116.44
61.26
45.43
39.52 DMSO-d6
35.49
27.73
HO
O
O
NH
Br
N
N
S
O
O
F
65000
60000
55000
50000
45000
40000
35000
30000
25000
20000
15000
10000
5000
0
180
170
160
150
140
130
120
110
100
90
80
70
60
50
40
30
20
10
0
f1 (ppm)

VNI-5165

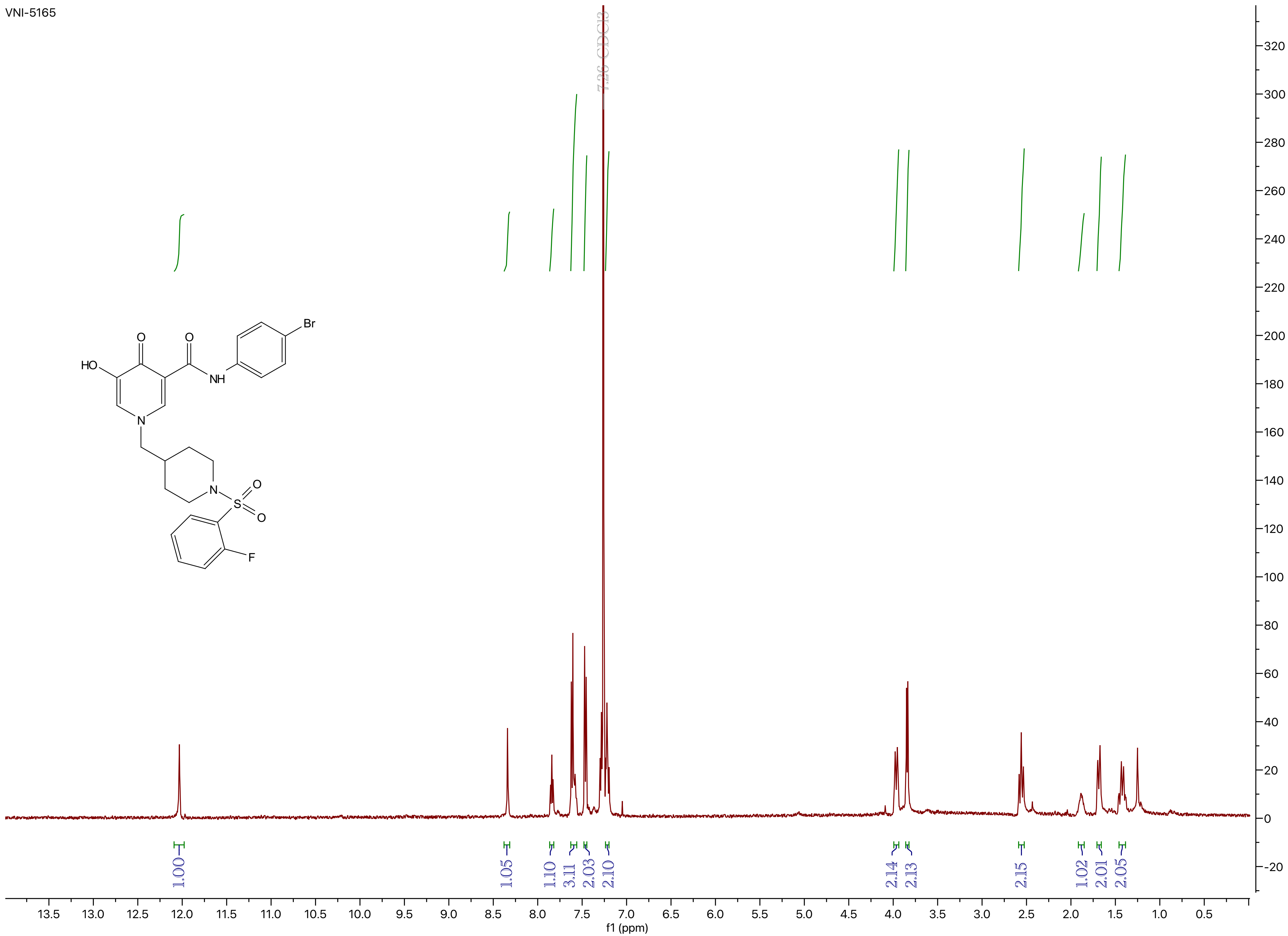

7.26 CDCl3
Br
O
O
HO
NH
N
N
S
O
O
F
1.00
1.05
1.10
3.11
2.03
2.10
2.14
2.13
2.15
1.02
2.01
2.05
13.5 13.0 12.5 12.0 11.5 11.0 10.5 10.0 9.5 9.0 8.5 8.0 7.5 7.0 6.5 6.0 5.5 5.0 4.5 4.0 3.5 3.0 2.5 2.0 1.5 1.0 0.5
f1 (ppm)
320 300 280 260 240 220 200 180 160 140 120 100 80 60 40 20 0 -20

VNI-5165

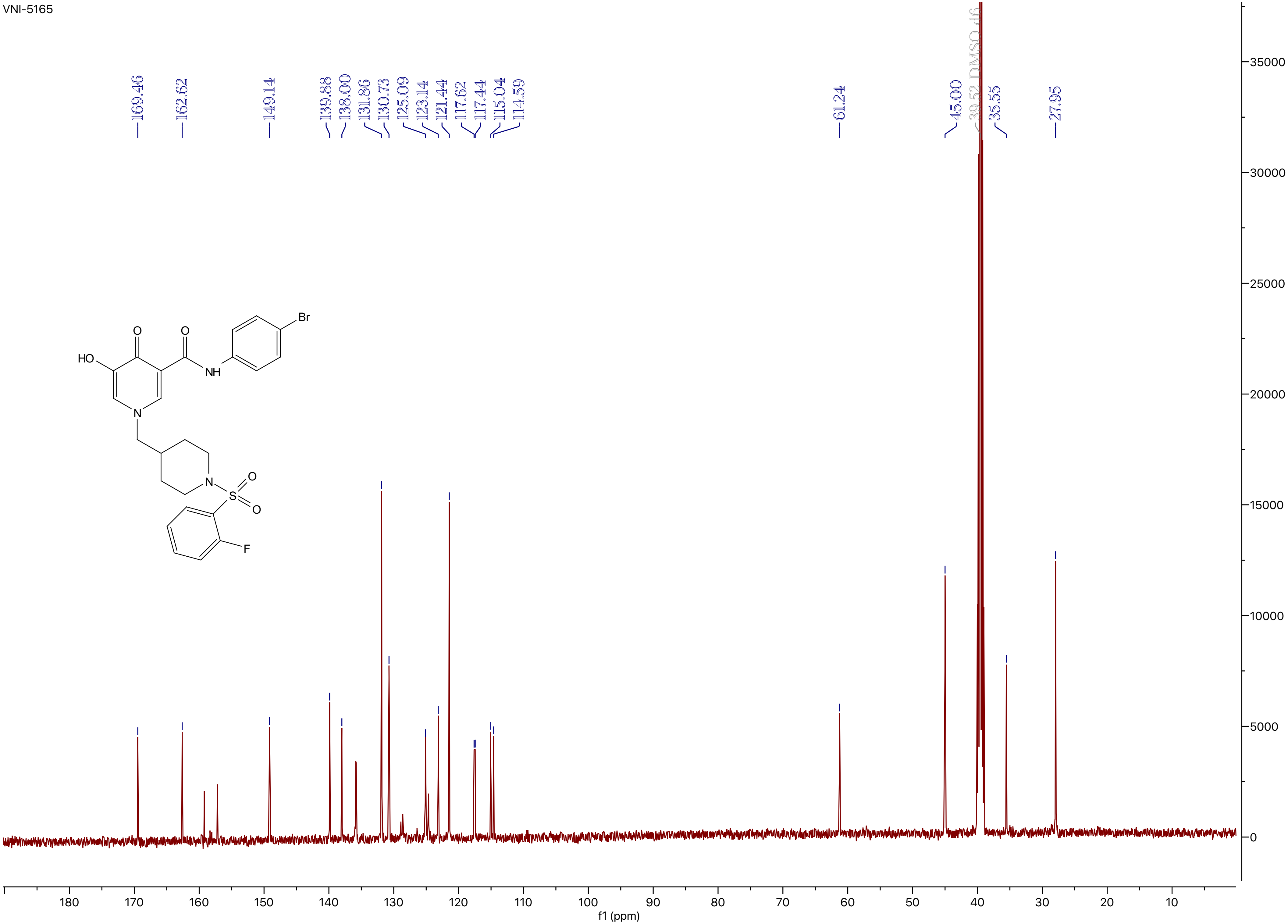

169.46
162.62
149.14
139.88
138.00
131.86
130.73
125.09
123.14
121.44
117.62
117.44
115.04
114.59
61.24
45.00
39.52 DMSO-d6
35.55
27.95
35000
30000
25000
20000
15000
10000
5000
0
180
170
160
150
140
130
120
110
100
90
80
70
60
50
40
30
20
10
f1 (ppm)
HO
O
O
NH
Br
N
N
S
O
O
F

VNI-5166

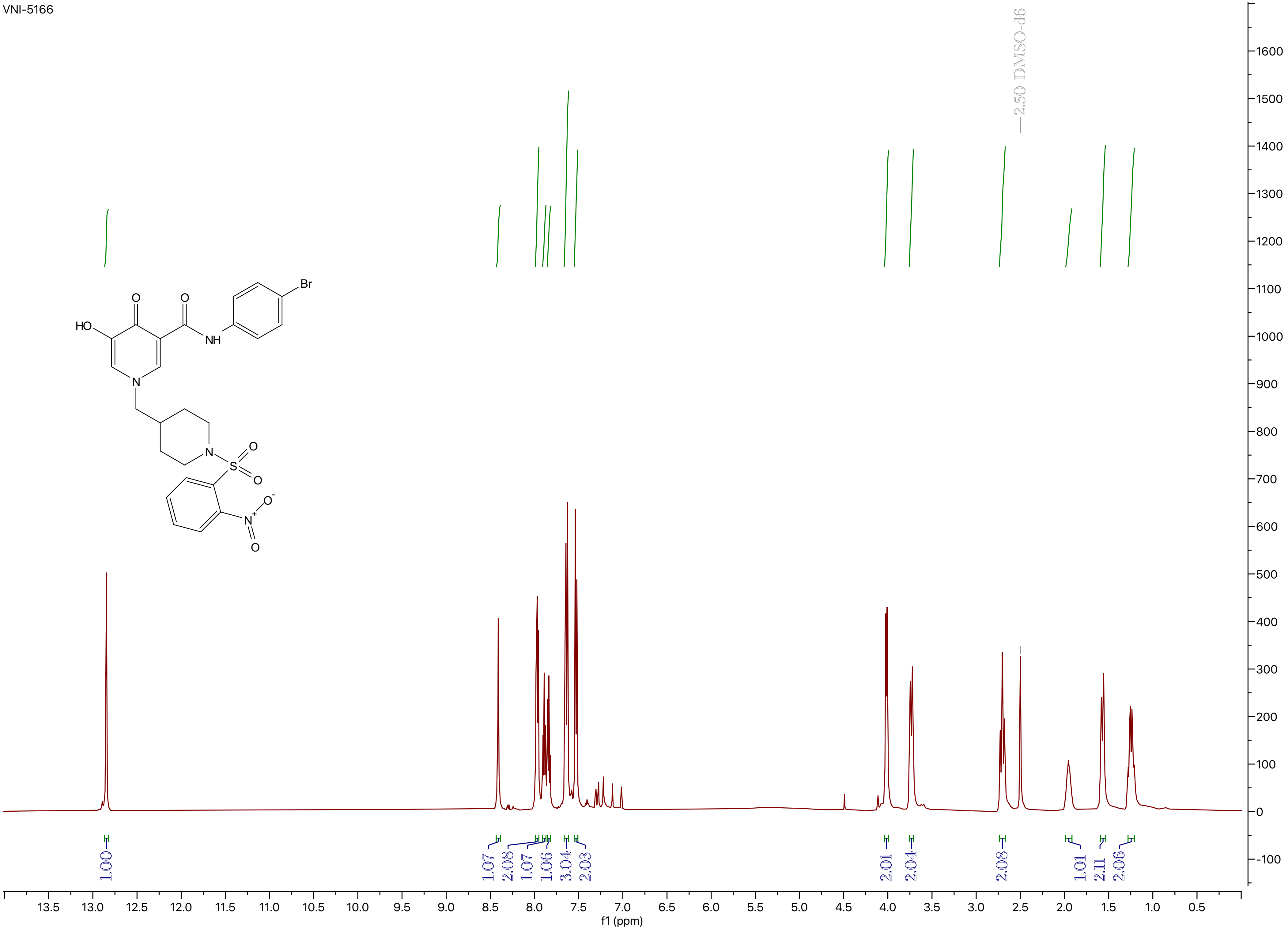

—2.50 DMSO-d6
HO
O
O
NH
Br
N
N
S
O
O
N+
O-
O
1.00
1.07
2.08
1.07
1.06
3.04
2.03
2.01
2.04
2.08
1.01
2.11
2.06
13.5 13.0 12.5 12.0 11.5 11.0 10.5 10.0 9.5 9.0 8.5 8.0 7.5 7.0 6.5 6.0 5.5 5.0 4.5 4.0 3.5 3.0 2.5 2.0 1.5 1.0 0.5
f1 (ppm)
1600 1500 1400 1300 1200 1100 1000 900 800 700 600 500 400 300 200 100 0 -100

VNI-5166

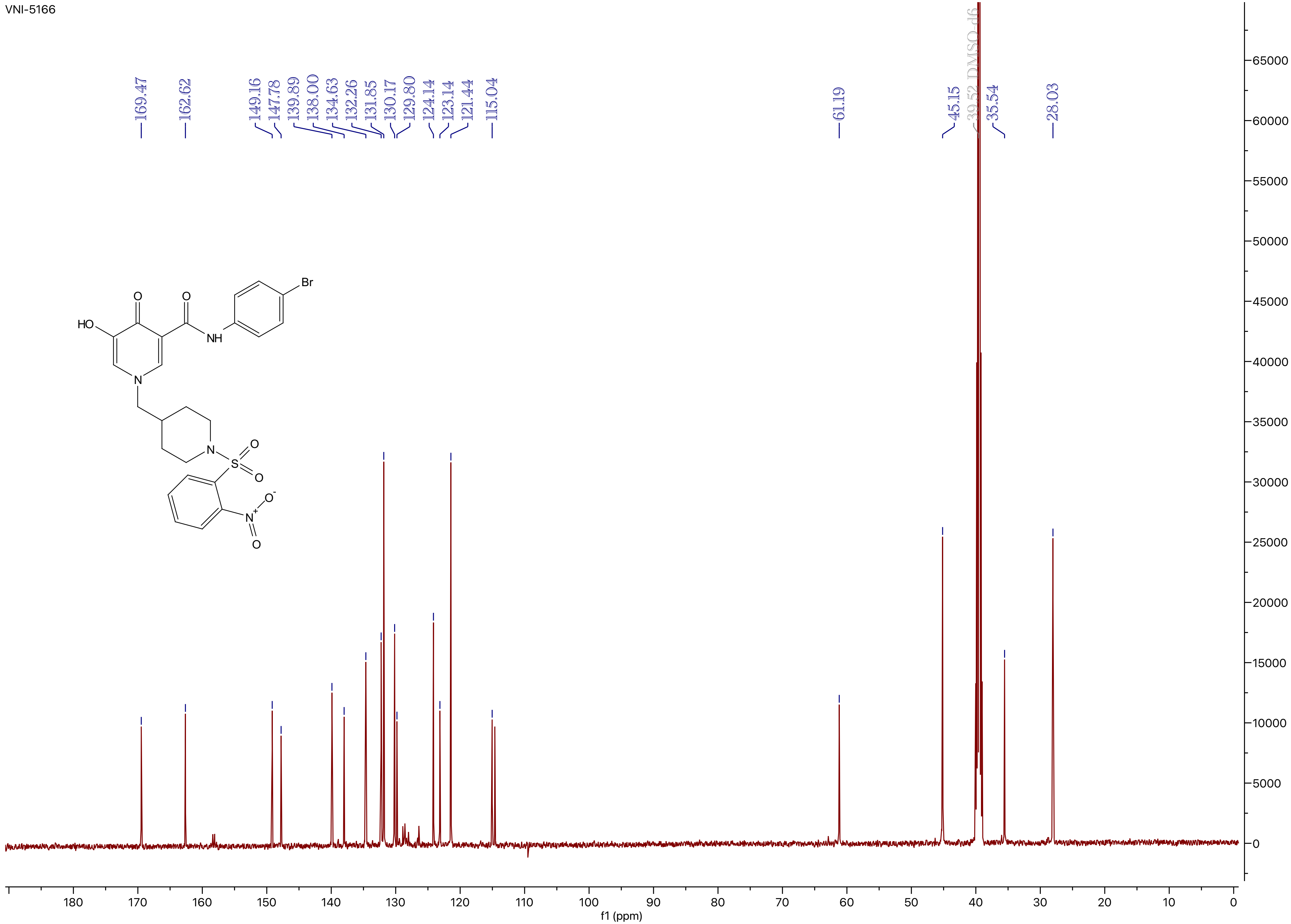

169.47
162.62
149.16
147.78
139.89
138.00
134.63
132.26
131.85
130.17
129.80
124.14
123.14
121.44
115.04
61.19
45.15
39.52 DMSO-d6
35.54
28.03
HO
O
O
NH
Br
N
N
S
O
O
N
O
O
f1 (ppm)

VNI-5167

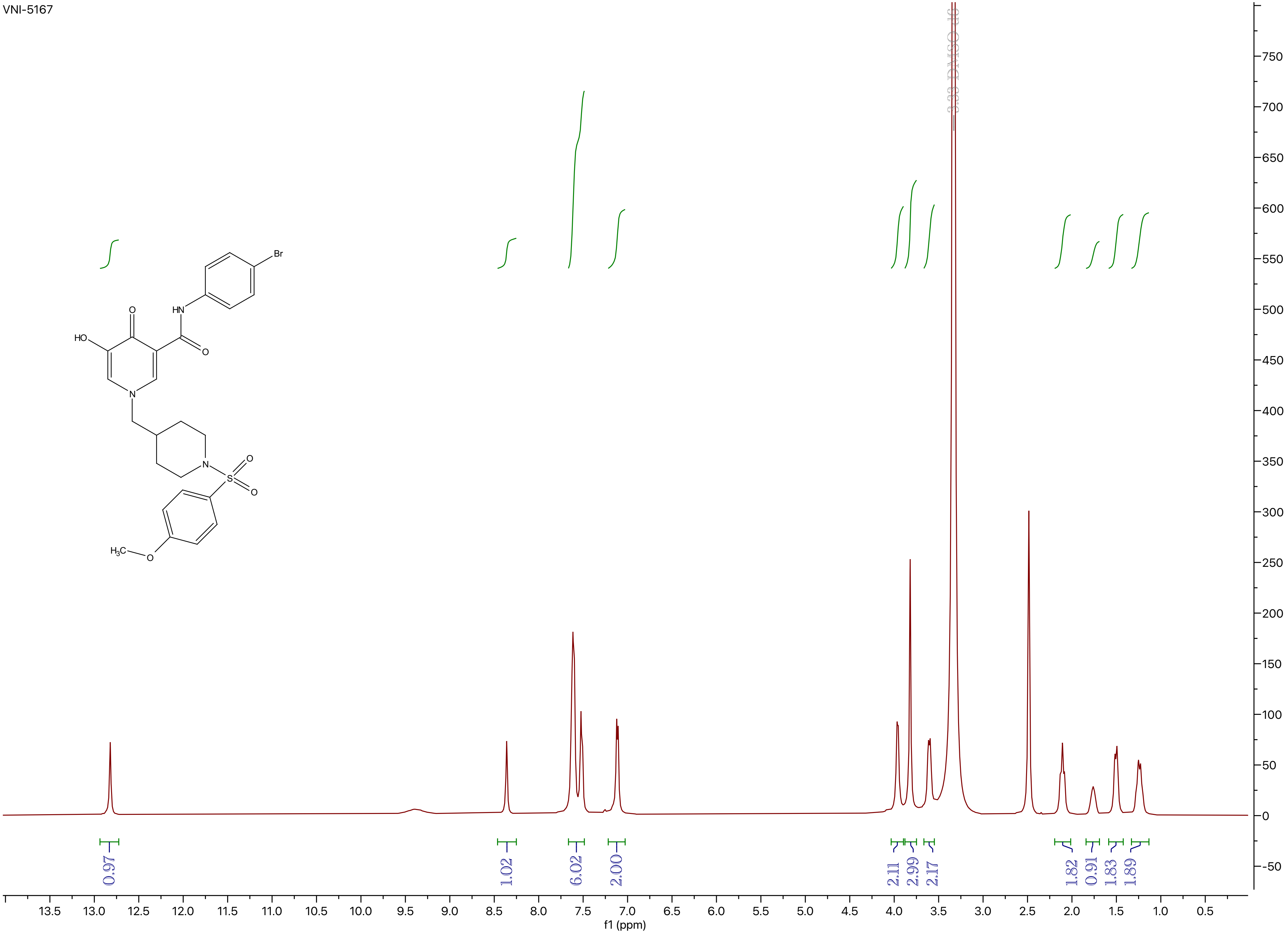

Br
HN
O
HO
O
N
N
S
O
O
H3C
O
3.33 DMSO-d6
0.97
1.02
6.02
2.00
2.11
2.99
2.17
1.82
0.91
1.83
1.89
13.5 13.0 12.5 12.0 11.5 11.0 10.5 10.0 9.5 9.0 8.5 8.0 7.5 7.0 6.5 6.0 5.5 5.0 4.5 4.0 3.5 3.0 2.5 2.0 1.5 1.0 0.5
f1 (ppm)
750 700 650 600 550 500 450 400 350 300 250 200 150 100 50 0 -50

VNI-5167

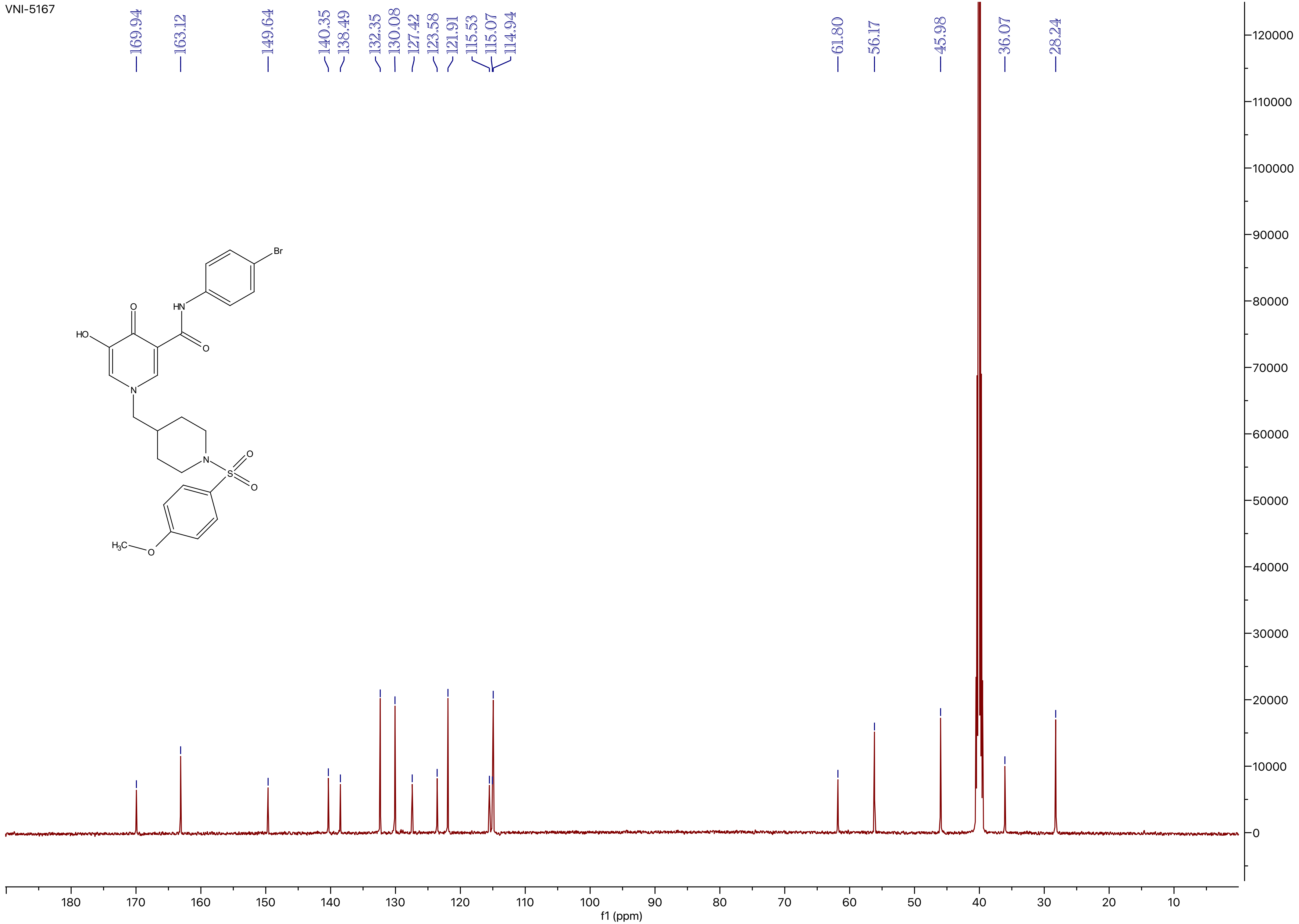
169.94
163.12
149.64
140.35
138.49
132.35
130.08
127.42
123.58
121.91
115.53
115.07
114.94
61.80
56.17
45.98
36.07
28.24
Br
HN
O
HO
O
N
N
S
O
O
H3C
O
120000
110000
100000
90000
80000
70000
60000
50000
40000
30000
20000
10000
0
180
170
160
150
140
130
120
110
100
90
80
70
60
50
40
30
20
10
f1 (ppm)

VNI-5168

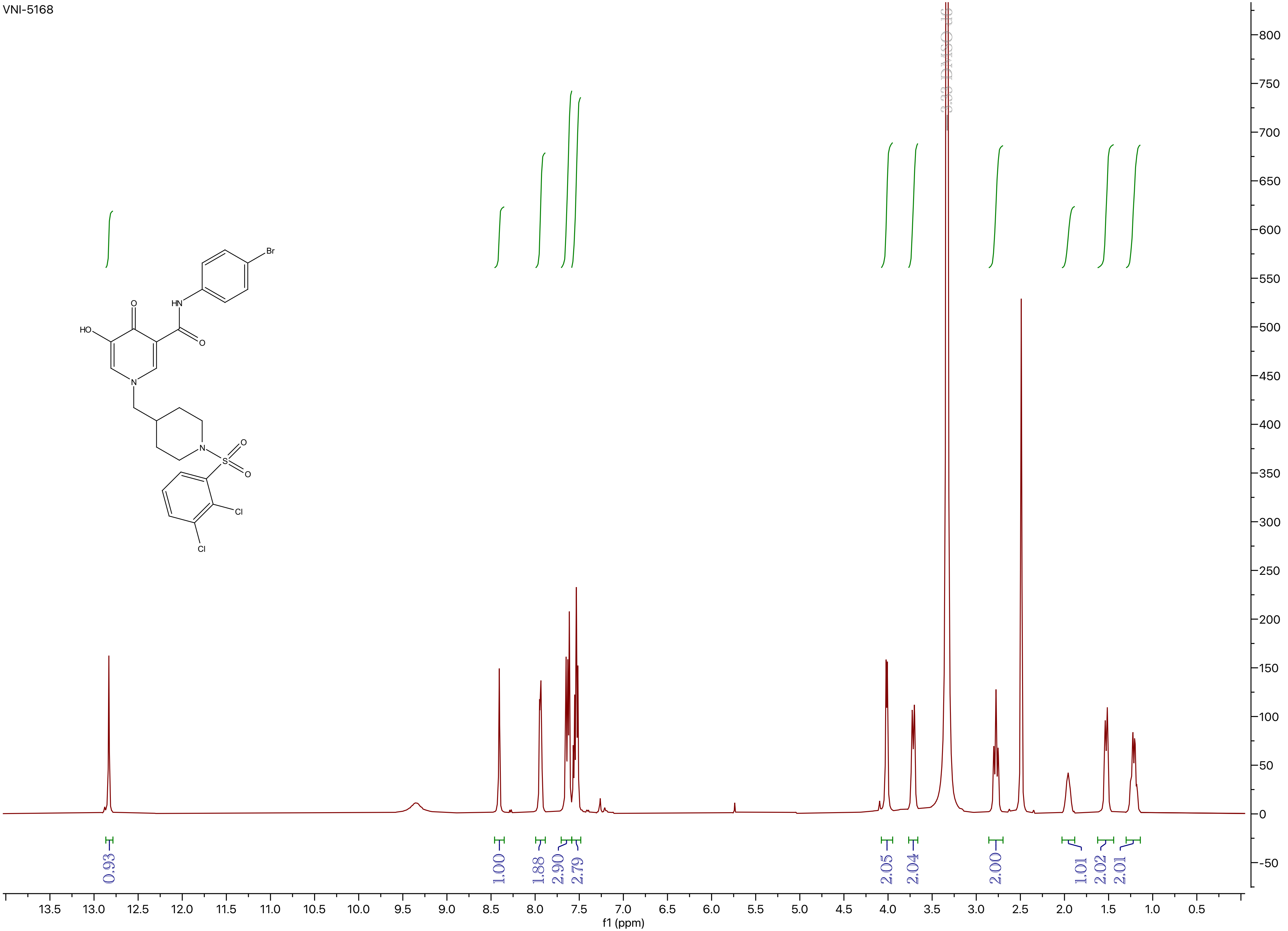

Br
HN
O
HO
O
N
N
S
O
O
Cl
Cl
3.33 DMSO-d6
0.93
1.00
1.88
2.90
2.79
2.05
2.04
2.00
1.01
2.02
2.01
f1 (ppm)

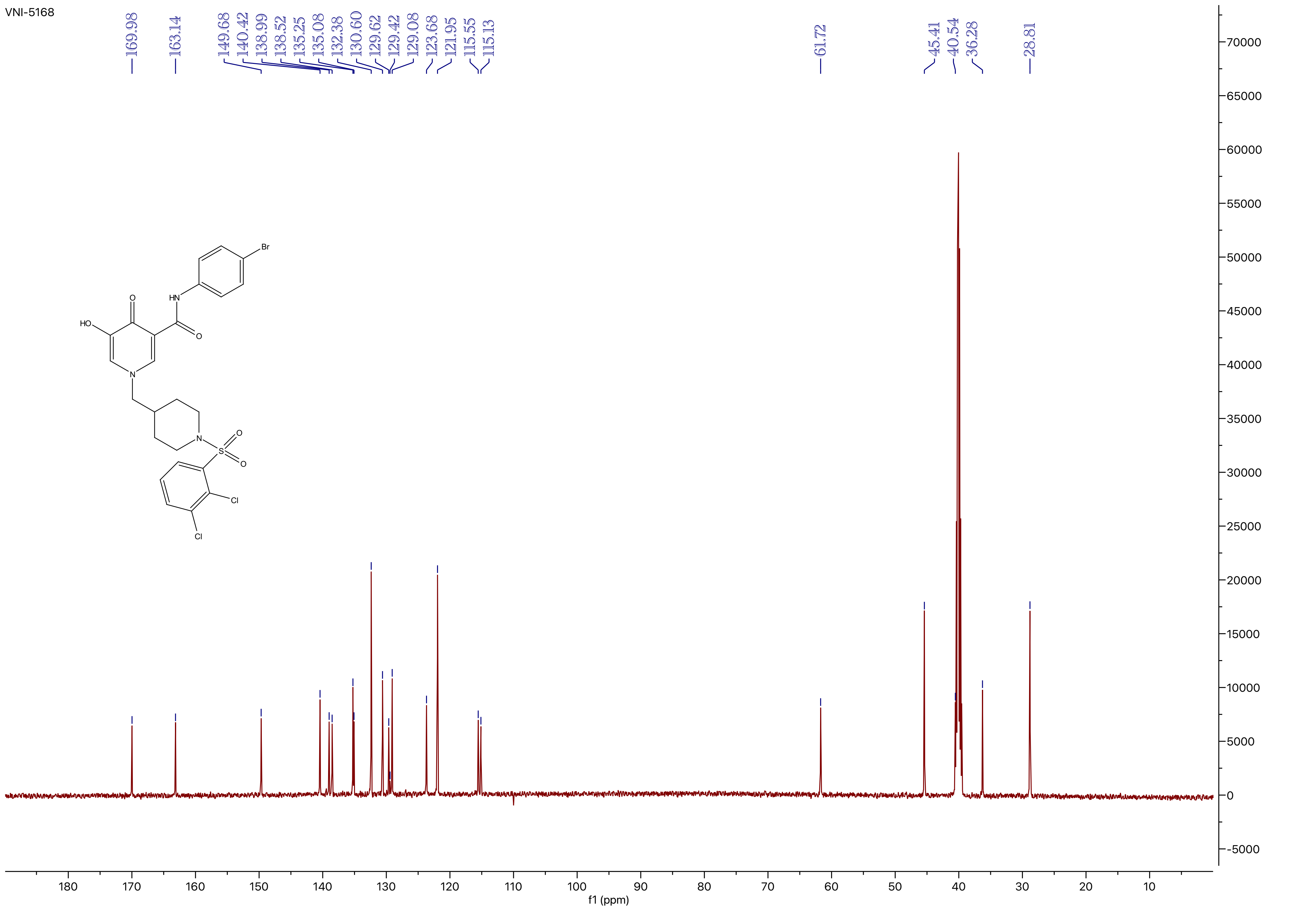
VNI-5168
169.98
163.14
149.68
140.42
138.99
138.52
135.25
135.08
132.38
130.60
129.62
129.42
129.08
123.68
121.95
115.55
115.13
61.72
45.41
40.54
36.28
28.81
Br
HN
O
HO
O
N
N
S
O
O
Cl
Cl
70000
65000
60000
55000
50000
45000
40000
35000
30000
25000
20000
15000
10000
5000
0
-5000
180
170
160
150
140
130
120
110
100
90
80
70
60
50
40
30
20
10
f1 (ppm)

VNI-5170

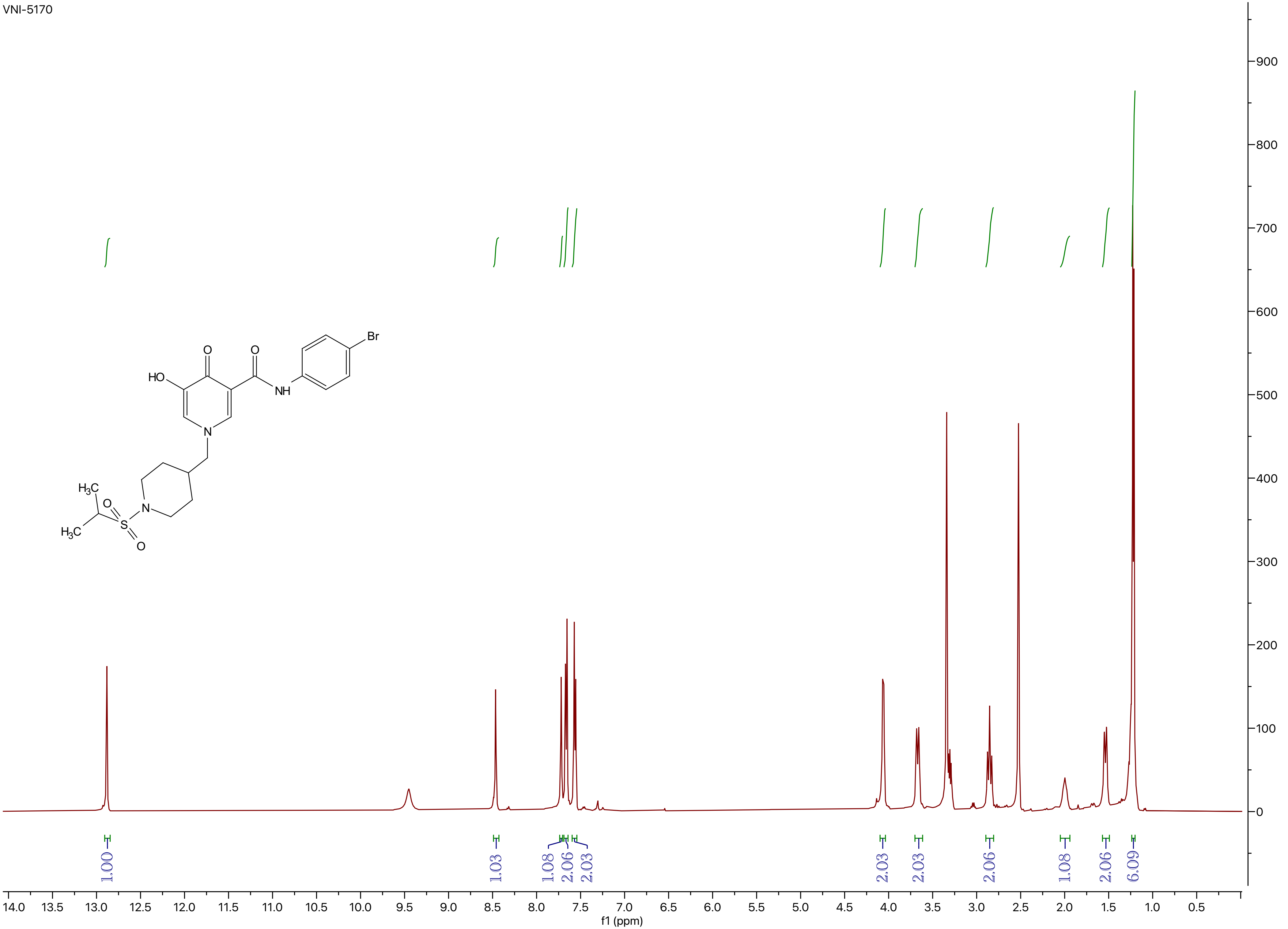

HO
O
O
NH
Br
N
H_3C
H_3C
O
S
O
N
900
800
700
600
500
400
300
200
100
0
1.00
1.03
1.08
2.06
2.03
2.03
2.03
2.06
1.08
2.06
6.09
14.0
13.5
13.0
12.5
12.0
11.5
11.0
10.5
10.0
9.5
9.0
8.5
8.0
7.5
7.0
6.5
6.0
5.5
5.0
4.5
4.0
3.5
3.0
2.5
2.0
1.5
1.0
0.5
f1 (ppm)

VNI-5170

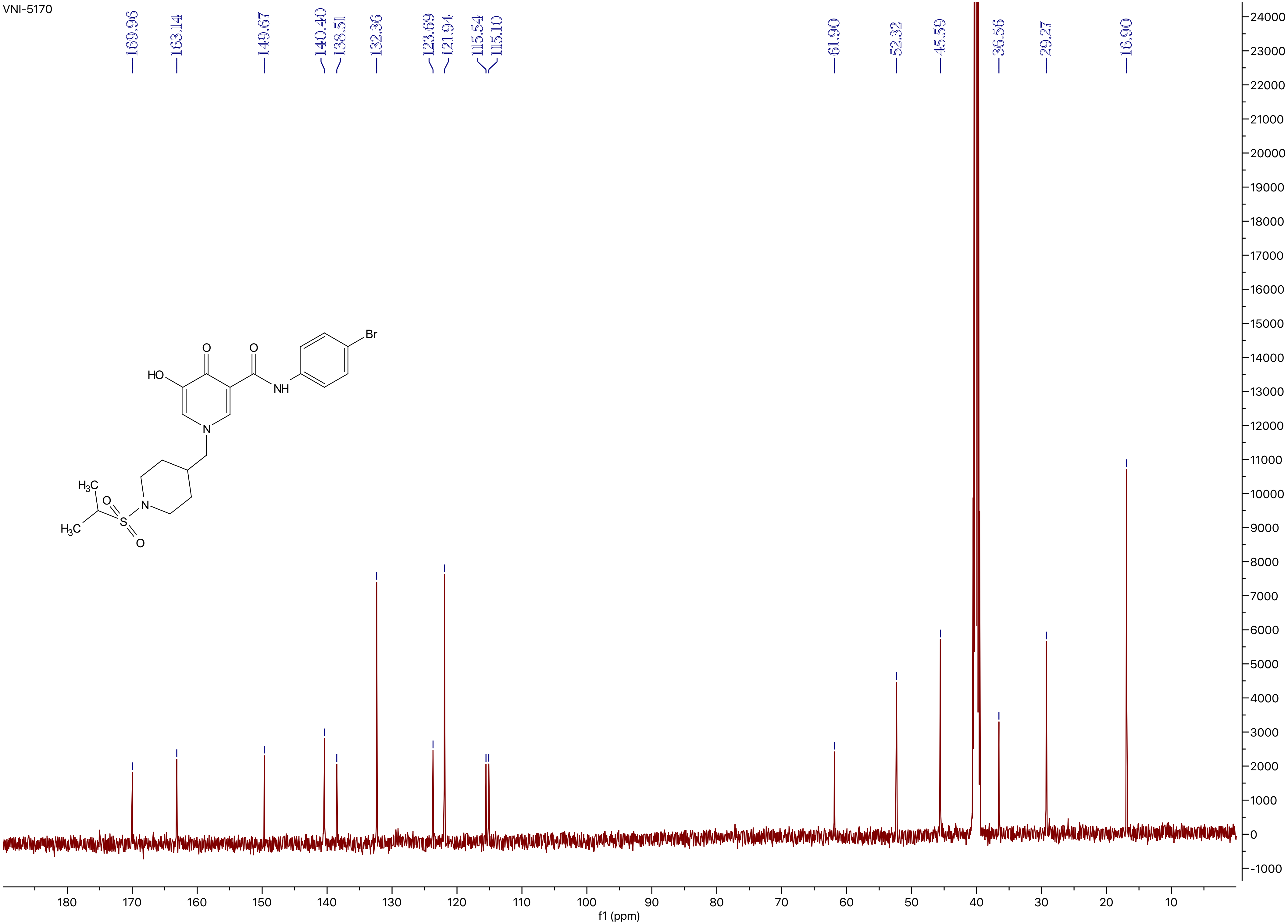
169.96
163.14
149.67
140.40
138.51
132.36
123.69
121.94
115.54
115.10
61.90
52.32
45.59
36.56
29.27
16.90
Br
O
O
HO
NH
N
H_3C
O
S
N
H_3C
O
24000
23000
22000
21000
20000
19000
18000
17000
16000
15000
14000
13000
12000
11000
10000
9000
8000
7000
6000
5000
4000
3000
2000
1000
0
-1000
180
170
160
150
140
130
120
110
100
90
80
70
60
50
40
30
20
10
f1 (ppm)

VNI-5171

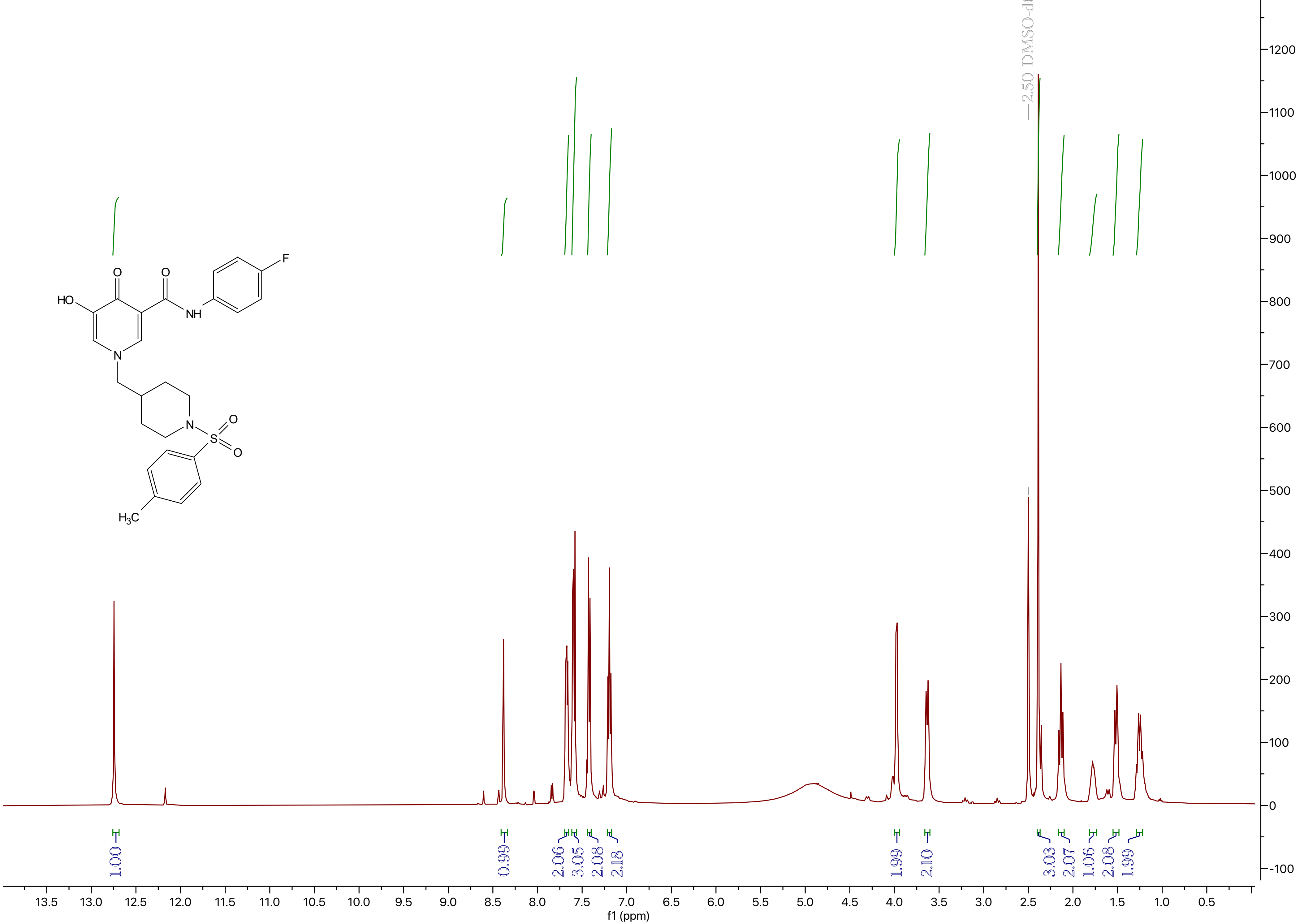

2.50 DMSO-d6
HO
O
O
NH
F
N
N
S
O
O
H3C
1.00
0.99
2.06
3.05
2.08
2.18
1.99
2.10
3.03
2.07
1.06
2.08
1.99
13.5 13.0 12.5 12.0 11.5 11.0 10.5 10.0 9.5 9.0 8.5 8.0 7.5 7.0 6.5 6.0 5.5 5.0 4.5 4.0 3.5 3.0 2.5 2.0 1.5 1.0 0.5
f1 (ppm)
1200 1100 1000 900 800 700 600 500 400 300 200 100 0 -100

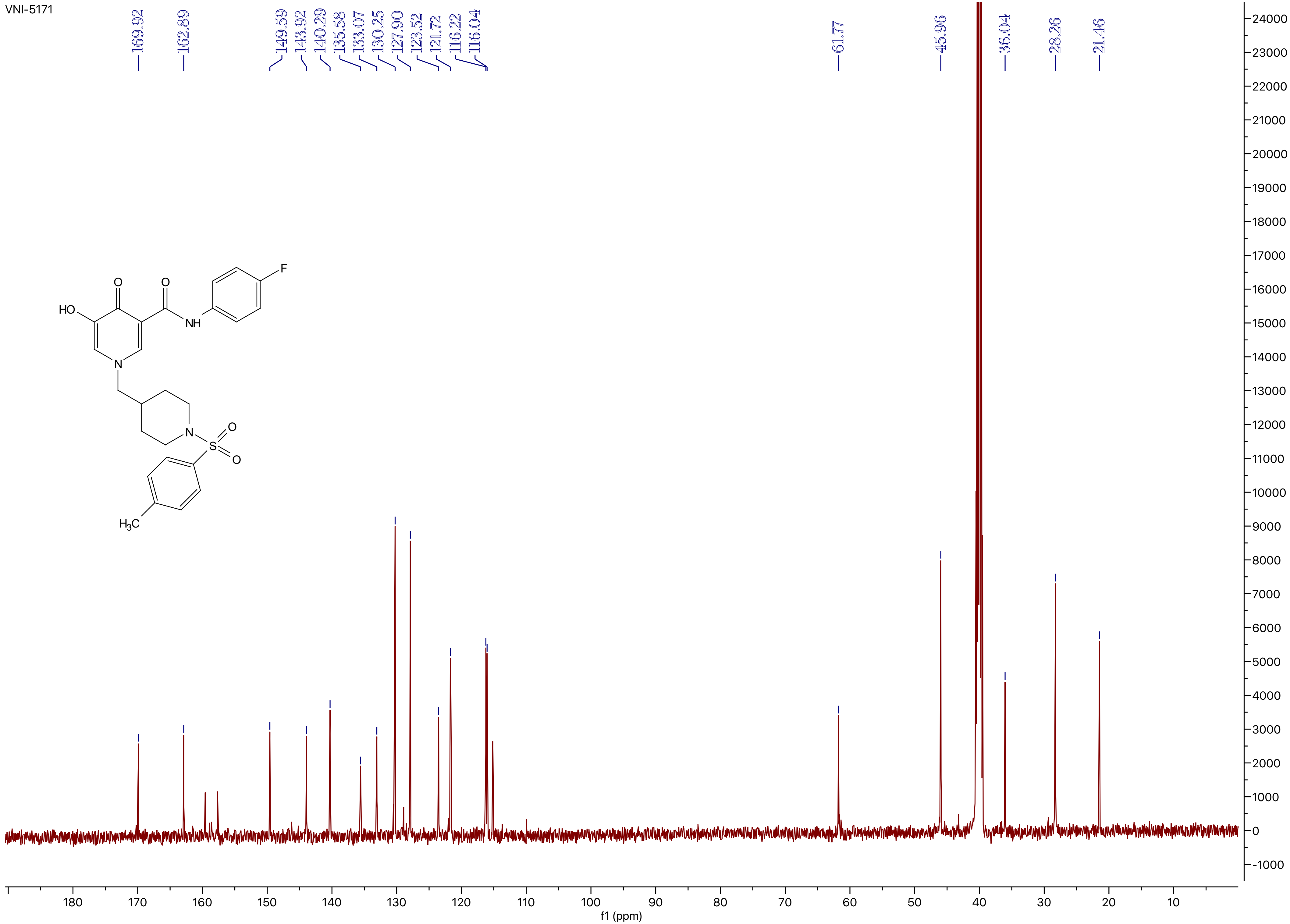

VNI-5171
169.92
162.89
149.59
143.92
140.29
135.58
133.07
130.25
127.90
123.52
121.72
116.22
116.04
61.77
45.96
36.04
28.26
21.46
HO
O
O
NH
F
N
N
S
O
O
H_3C
f1 (ppm)

VNI-5172

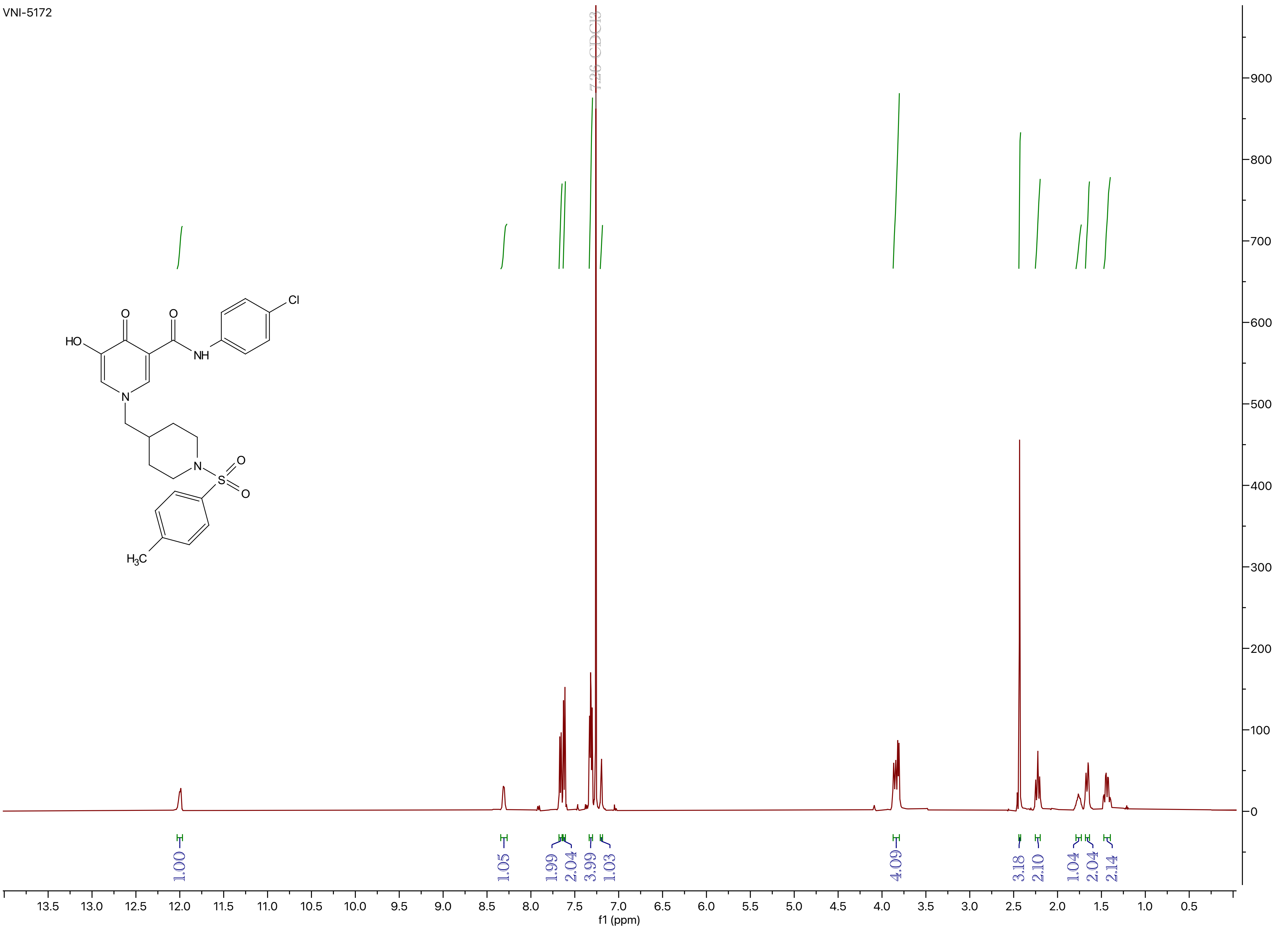

7.26 CDCl3
HO
O
O
NH
Cl
N
N
S
O
O
H3C
1.00
1.05
1.99
2.04
3.99
1.03
4.09
3.18
2.10
1.04
2.04
2.14
13.5 13.0 12.5 12.0 11.5 11.0 10.5 10.0 9.5 9.0 8.5 8.0 7.5 7.0 6.5 6.0 5.5 5.0 4.5 4.0 3.5 3.0 2.5 2.0 1.5 1.0 0.5
f1 (ppm)
900 800 700 600 500 400 300 200 100 0

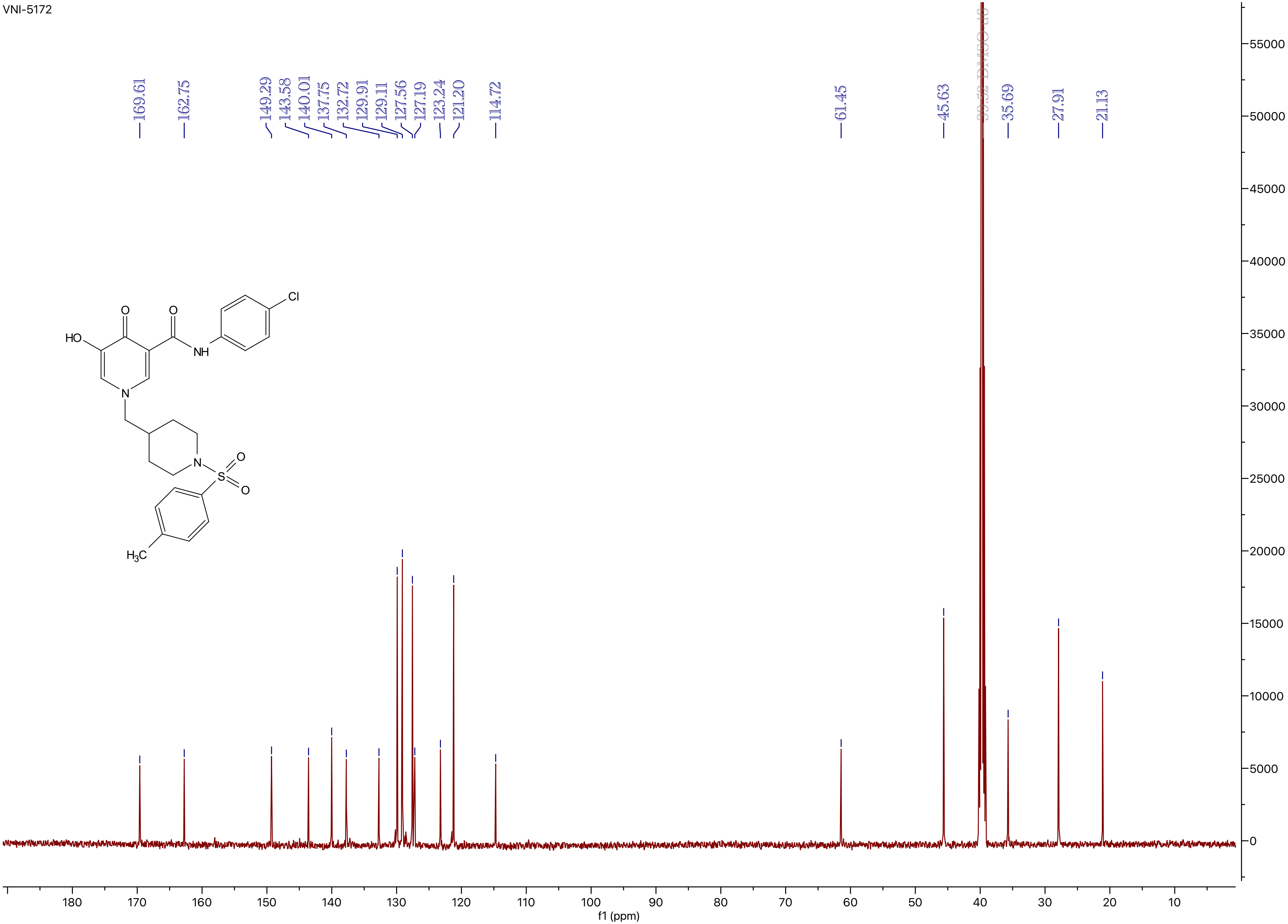
VNI-5172
169.61
162.75
149.29
143.58
140.01
137.75
132.72
129.91
129.11
127.56
127.19
123.24
121.20
114.72
61.45
45.63
35.69
27.91
21.13
HO
O
O
NH
Cl
N
N
S
O
O
H3C
55000
50000
45000
40000
35000
30000
25000
20000
15000
10000
5000
0
180
170
160
150
140
130
120
110
100
90
80
70
60
50
40
30
20
10
f1 (ppm)
